# Supplementary material for: Repeatability and genetic advances in early maturing maize hybrid trials conducted under Striga-infested and non-infested conditions
Source: PLoS One. 2025 Apr 3;20(4):e0319353. doi: 10.1371/journal.pone.0319353 (PMC11967925; doi:10.1371/journal.pone.0319353)
Supplement: S1 Files — (DOCX) [file pone.0319353.s001.docx]

**Table S1**. Mean grain yield and other Striga adaptive traits, year of first testing in the regional trial of the multiple stress tolerant hybrids under STRINF condition in Nigeria between 2010 –2021

| **Serial**  **number** | **Year of**  **first testing** | **Code** | **PEDIGREE** | **YLD** | **DA** | **DS** | **ASI** | **EASP** | **EPP** | **ESP8** | **ESP10** | **SDR8** | **SDR10** |
| --- | --- | --- | --- | --- | --- | --- | --- | --- | --- | --- | --- | --- | --- |
| 1 | 2010 | MST1 | (TZEI2xTZEI108)xTZEI63 | 2283.6 | 55.4 | 56.9 | 2.2 | 5.2 | 0.7 | 27.8 | 34.6 | 5.2 | 5.6 |
| 2 | 2010 | MST2 | (TZEI59xTZEI108)xTZEI63 | 2011.2 | 55.3 | 57.1 | 3.0 | 5.4 | 0.7 | 44.6 | 43.3 | 5.2 | 5.6 |
| 3 | 2010 | MST3 | (TZEI63xTZEI108)x(TZEI59xTZEI87) | 2047.1 | 54.7 | 56.5 | 2.4 | 5.2 | 0.7 | 30.9 | 27.2 | 5.1 | 5.8 |
| 4 | 2010 | MST4 | (TZEI63xTZEI59)x(TZEI108xTZEI87) | 2014.3 | 55.0 | 56.6 | 2.5 | 5.4 | 0.7 | 30.5 | 32.5 | 5.2 | 6.1 |
| 5 | 2010 | MST5 | TZEWPopDTSTRC4xTZEI22 | 2448.1 | 55.7 | 57.4 | 2.6 | 5.2 | 0.7 | 47.7 | 52.1 | 4.8 | 5.5 |
| 6 | 2010 | MST6 | TZEWPopDTSTRC4xTZEI7 | 2703.9 | 56.7 | 57.7 | 1.6 | 4.7 | 0.7 | 30.1 | 36.8 | 4.7 | 5.6 |
| 7 | 2010 | MST7 | TZEI5xTZEI98 | 2866.2 | 57.0 | 59.4 | 3.3 | 4.7 | 0.8 | 21.5 | 29.1 | 5.3 | 5.5 |
| 8 | 2010 | MST8 | TZEI1xTZEI5 | 1968.0 | 57.0 | 58.8 | 3.6 | 5.7 | 0.7 | 41.1 | 51.3 | 5.2 | 6.1 |
| 9 | 2010 | MST9 | TZEI26xTZEI5 | 1727.6 | 56.2 | 57.6 | 2.6 | 5.5 | 0.5 | 35.2 | 36.6 | 6.1 | 7.1 |
| 10 | 2010 | MST10 | (TZEI135xTZEI157)x(TZEI17xTZEI16) | 2413.5 | 54.5 | 56.7 | 2.6 | 5.1 | 0.7 | 9.4 | 24.2 | 4.8 | 6.0 |
| 11 | 2010 | MST11 | (TZEI135xTZEI157)xTZEI17 | 2361.8 | 56.3 | 57.7 | 2.2 | 5.0 | 0.8 | -2.2 | 21.6 | 4.3 | 5.2 |
| 12 | 2010 | MST12 | (TZEI135xTZEI16)x(TZEI129xTZEI17) | 2441.1 | 55.2 | 56.8 | 0.6 | 5.1 | 0.7 | 28.7 | 25.5 | 4.6 | 5.7 |
| 13 | 2010 | MST13 | (TZEI17xTZEI16)xTZEI157 | 2567.7 | 55.4 | 57.5 | 4.2 | 5.3 | 0.8 | 35.5 | 33.8 | 4.8 | 5.6 |
| 14 | 2010 | MST14 | TZEI11xTZEI25 | 2891.5 | 54.0 | 55.5 | 1.9 | 4.7 | 0.9 | 28.5 | 28.2 | 3.6 | 4.6 |
| 15 | 2010 | MST15 | TZEI136xTZEI11 | 3415.8 | 56.7 | 58.3 | 1.9 | 4.6 | 1.0 | -4.4 | 13.8 | 3.5 | 4.2 |
| 16 | 2010 | MST16 | TZEI136xTZEI14 | 3590.9 | 55.5 | 56.9 | 0.6 | 4.6 | 0.9 | 13.5 | 19.2 | 4.0 | 4.5 |
| 17 | 2010 | MST17 | TZEI14xTZEI25 | 3059.5 | 55.0 | 56.3 | 0.6 | 4.5 | 0.8 | 23.3 | 26.9 | 3.9 | 4.7 |
| 18 | 2010 | MST18 | TZEI16xTZEI8 | 2980.9 | 54.1 | 56.1 | 1.6 | 4.5 | 0.9 | 12.4 | 19.2 | 3.8 | 4.4 |
| 19 | 2010 | MST19 | TZEI17xTZEI135 | 2424.8 | 55.7 | 58.4 | 3.6 | 5.2 | 0.7 | 11.3 | 16.5 | 4.7 | 5.6 |
| 20 | 2010 | MST20 | TZEI24xTZEI17 | 3498.7 | 55.4 | 56.5 | -0.8 | 4.5 | 0.9 | 10.6 | 19.1 | 3.8 | 4.4 |
| 21 | 2010 | MST21 | TZEI8xTZEI17 | 2405.2 | 55.4 | 57.2 | 1.2 | 5.4 | 0.9 | 25.1 | 29.2 | 4.9 | 5.4 |
| 22 | 2010 | MST22 | TZEI9xTZEI16 | 2562.4 | 55.0 | 56.6 | 4.2 | 5.3 | 0.7 | 14.4 | 34.5 | 4.3 | 5.5 |
| 23 | 2010 | MST23 | TZEYPopDTSTRC4xTZEI11 | 3165.7 | 56.3 | 57.5 | 1.9 | 4.7 | 0.9 | 20.7 | 41.8 | 4.0 | 4.5 |
| 24 | 2010 | MST24 | TZEYPopDTSTRC4xTZEI17 | 2980.5 | 55.5 | 58.0 | 0.9 | 4.7 | 0.9 | 26.2 | 32.2 | 4.1 | 5.1 |
| 25 | 2010 | MST25 | (TZEI2XTZEI63)(TZEI108XTZEI87) | 2472.3 | 53.8 | 55.6 | 2.5 | 5.1 | 0.8 | 28.8 | 26.0 | 5.1 | 5.8 |
| 26 | 2010 | MST26 | (TZEI2XTZEI63)(TZEI59XTZEI87) | 2103.5 | 55.2 | 56.8 | 2.4 | 5.0 | 0.7 | 39.5 | 39.1 | 5.7 | 6.5 |
| 27 | 2010 | MST27 | (TZEI63XTZEI108)(TZEI59XTZEI87) | 3065.1 | 54.3 | 55.8 | 2.3 | 4.1 | 0.7 | 29.4 | 28.6 | 5.2 | 6.0 |
| 28 | 2010 | MST28 | (TZEI63XTZEI87)(TZEI59XTZE108) | 2079.8 | 54.7 | 55.8 | 2.1 | 5.1 | 0.7 | 44.3 | 48.4 | 5.5 | 6.7 |
| 29 | 2010 | MST29 | TZEI31xTZEI18 | 2280.2 | 53.8 | 55.6 | 2.4 | 5.3 | 0.6 | 29.6 | 31.4 | 5.2 | 5.8 |
| 30 | 2011 | MST30 | TZEI4xTZEI7 | 2926.7 | 56.7 | 58.3 | 2.4 | 5.2 | 0.8 | 25.0 | 29.8 | 4.5 | 5.1 |
| 31 | 2011 | MST31 | TZEI3xTZEI4 | 2474.1 | 57.1 | 58.5 | 1.2 | 5.1 | 0.8 | 23.0 | 21.9 | 4.8 | 5.8 |
| 32 | 2011 | MST32 | TZEI3xTZEI26 | 2481.7 | 56.0 | 57.8 | 3.2 | 5.0 | 0.8 | 35.2 | 28.0 | 5.2 | 5.7 |
| 33 | 2011 | MST33 | TZEI7xTZEI26 | 2030.5 | 55.0 | 57.6 | 3.2 | 5.8 | 0.7 | 39.8 | 41.9 | 5.5 | 6.1 |
| 34 | 2011 | MST34 | TZEI1xTZEI3 | 2561.0 | 57.1 | 59.6 | 2.8 | 4.7 | 0.7 | 17.0 | 20.8 | 4.9 | 5.3 |
| 35 | 2011 | MST35 | (TZEI63XTZEI59)xTZEI87 | 1176.4 | 54.7 | 57.1 | 3.4 | 5.8 | 0.5 | 43.1 | 44.1 | 6.4 | 6.8 |
| 36 | 2011 | MST36 | (TZEI63XTZEI87)XTZEI59 | 1993.9 | 55.2 | 58.3 | 4.1 | 5.4 | 0.8 | 38.4 | 37.4 | 5.4 | 6.4 |
| 37 | 2011 | MST37 | TZEI59xTZEI63 | 1678.7 | 55.1 | 57.9 | 2.4 | 5.0 | 0.6 | 35.6 | 37.8 | 6.5 | 6.8 |
| 38 | 2011 | MST38 | (TZEI63XTZEI108)xTZEI87 | 1122.4 | 54.3 | 56.4 | 3.7 | 6.3 | 0.6 | 38.3 | 45.7 | 6.5 | 7.1 |
| 39 | 2012 | MST39 | (TZEI63XTZEI87)x(TZEI59XTZE108) | 1611.4 | 53.3 | 56.0 | 2.7 | 5.7 | 0.7 | 39.8 | 41.0 | 5.9 | 6.6 |
| 40 | 2012 | MST40 | (TZEI2xTZEI63)x(TZEI108xTZEI87) | 2689.4 | 55.0 | 56.2 | 2.1 | 5.3 | 0.8 | 23.6 | 25.0 | 5.2 | 5.6 |
| 41 | 2012 | MST41 | (TZEI2XTZEI63)x(TZEI59XTZEI87) | 1091.1 | 54.1 | 56.5 | 3.1 | 6.0 | 0.5 | 49.0 | 53.6 | 7.3 | 7.6 |
| 42 | 2012 | MST42 | TZEWPopDTSTRC4xTZEI19 | 2534.8 | 56.0 | 58.4 | 3.2 | 4.9 | 0.7 | 30.6 | 39.1 | 4.5 | 4.7 |
| 43 | 2012 | MST43 | TZEWPopDTSTRxTZEI5 | 1497.8 | 58.5 | 62.4 | 2.4 | 5.5 | 0.6 | 26.9 | 33.2 | 5.7 | 6.2 |
| 44 | 2012 | MST44 | TZEI188xTZEI98 | 2598.7 | 55.8 | 58.8 | 2.5 | 4.8 | 0.7 | 22.5 | 27.8 | 4.5 | 5.3 |
| 45 | 2012 | MST45 | TZEI5xTZEI60 | 2802.5 | 58.1 | 60.3 | 2.9 | 4.6 | 0.7 | 30.1 | 29.1 | 4.7 | 5.5 |
| 46 | 2012 | MST46 | TZEI83xTZEI60 | 2542.6 | 57.5 | 58.5 | 1.8 | 5.0 | 0.7 | 29.6 | 32.4 | 4.7 | 4.9 |
| 47 | 2013 | MST47 | TZEI2xTZEI87 | 3375.2 | 56.7 | 57.5 | 0.2 | 4.7 | 0.9 | 10.2 | 18.2 | 3.9 | 4.7 |
| 48 | 2013 | MST48 | (TZEI63xTZEI87)x(TZEI59xTZEI108) | 1944.4 | 54.3 | 55.3 | 1.9 | 5.3 | 0.7 | 37.9 | 34.4 | 5.3 | 6.0 |
| 49 | 2013 | MST49 | TZE-WPopDTSTRC4xTZEI19 | 2267.0 | 55.0 | 57.5 | 3.4 | 5.2 | 0.6 | 47.7 | 51.0 | 4.9 | 5.8 |
| 50 | 2013 | MST50 | TZEI31xTZEI63 | 1183.5 | 52.5 | 55.5 | 2.8 | 6.2 | 0.6 | 30.9 | 37.4 | 6.7 | 7.2 |
| 51 | 2013 | MST51 | ENT12xTZEI48 | 3098.8 | 57.5 | 58.5 | 1.9 | 4.8 | 0.8 | 28.0 | 34.2 | 3.8 | 4.6 |
| 52 | 2013 | MST52 | ENT10xTZEI82 | 2491.2 | 56.6 | 58.6 | 2.6 | 5.0 | 0.8 | 32.4 | 29.2 | 4.9 | 5.5 |
| 53 | 2013 | MST53 | ENT3xTZEI65 | 2346.7 | 56.3 | 57.4 | 2.3 | 4.9 | 0.7 | 44.7 | 40.3 | 4.3 | 5.1 |
| 54 | 2013 | MST54 | ENT7xTZEI60 | 2663.3 | 56.0 | 57.5 | 2.4 | 5.0 | 0.8 | 32.4 | 32.3 | 3.9 | 4.9 |
| 55 | 2013 | MST55 | DTE-WSTRSYNC1XENT12 | 3288.2 | 56.1 | 57.4 | 2.4 | 5.2 | 0.7 | 29.6 | 30.0 | 4.2 | 4.9 |
| 56 | 2013 | MST56 | TZE-WPopDTC2STRXENT20 | 2117.6 | 56.8 | 58.4 | 2.4 | 5.4 | 0.7 | 20.2 | 28.2 | 3.8 | 4.7 |
| 57 | 2013 | MST57 | TZEI32xTZEI56 | 3312.4 | 53.4 | 55.0 | 2.5 | 4.7 | 0.8 | 25.5 | 28.5 | 3.8 | 4.6 |
| 58 | 2013 | MST58 | ENT12xTZEI89 | 3390.2 | 55.9 | 57.2 | 2.2 | 4.4 | 0.9 | 20.1 | 29.1 | 4.6 | 5.4 |
| 59 | 2013 | MST59 | TZEI63XTZEI48 | 2682.5 | 55.8 | 55.8 | 1.0 | 4.6 | 0.8 | 48.0 | 48.6 | 4.3 | 5.2 |
| 60 | 2013 | MST60 | ENT11XTZEI22 | 3624.1 | 57.5 | 57.9 | 1.4 | 4.6 | 0.8 | 39.6 | 32.9 | 4.1 | 4.3 |
| 61 | 2013 | MST61 | TZE-WPopDTSTRC4xTZEI4 | 2707.9 | 55.2 | 56.9 | 2.4 | 5.4 | 0.7 | 25.6 | 30.0 | 4.0 | 4.6 |
| 62 | 2013 | MST62 | TZE-WPopDTSTRC4xTZEI22 | 2246.9 | 56.0 | 58.0 | 2.8 | 5.7 | 0.7 | 46.3 | 49.8 | 4.6 | 5.5 |
| 63 | 2013 | MST63 | TZE-WPopDTSTRC4xTZEI7 | 3319.7 | 55.7 | 56.9 | 2.1 | 5.1 | 0.8 | 26.8 | 32.4 | 4.2 | 5.1 |
| 64 | 2014 | MST64 | TZEI23xTZEI13 | 3443.7 | 54.7 | 56.4 | -0.1 | 4.5 | 1.0 | 7.9 | 20.3 | 3.7 | 4.5 |
| 65 | 2014 | MST65 | TZEI23xTZEI16 | 3095.6 | 55.4 | 56.8 | -0.8 | 4.5 | 1.0 | 20.2 | 27.9 | 3.6 | 4.5 |
| 66 | 2014 | MST66 | TZEI24xTZEI11 | 3550.7 | 54.5 | 55.7 | -0.4 | 4.6 | 0.9 | 8.1 | 12.9 | 3.5 | 4.1 |
| 67 | 2014 | MST67 | TZE-WPopDTSTRC4xTZEI5 | 2876.1 | 55.9 | 57.4 | 2.9 | 4.5 | 0.8 | 14.4 | 24.0 | 4.8 | 5.0 |
| 68 | 2014 | MST68 | TZE-WPopDTC2STRXENT16 | 2759.7 | 56.0 | 58.5 | 3.0 | 4.9 | 0.8 | 43.7 | 51.3 | 4.0 | 5.0 |
| 69 | 2014 | MST69 | EVDT-W99STR | 1963.4 | 56.2 | 57.9 | 2.7 | 5.7 | 0.6 | 49.2 | 47.5 | 6.1 | 6.6 |
| 70 | 2014 | MST70 | 2009TZEWDTSTR | 2392.5 | 56.3 | 58.6 | 3.2 | 5.3 | 0.7 | 39.3 | 52.7 | 4.7 | 5.6 |
| 71 | 2014 | MST71 | ENT11xTZEI4 | 3444.8 | 56.2 | 57.3 | 2.1 | 5.0 | 0.8 | 20.2 | 32.2 | 4.0 | 4.7 |
| 72 | 2015 | MST72 | TZdEI173xTZdEI378 | 3764.1 | 54.4 | 54.8 | 1.0 | 4.1 | 0.8 | 26.3 | 31.5 | 4.1 | 4.3 |
| 73 | 2015 | MST73 | ENT11xTZEI65 | 4255.6 | 54.7 | 55.3 | 1.3 | 4.2 | 0.9 | 24.6 | 31.2 | 3.5 | 4.4 |
| 74 | 2015 | MST74 | ENT11xTZEI19 | 3541.7 | 56.0 | 58.1 | 2.8 | 4.6 | 0.8 | 26.7 | 33.1 | 4.2 | 4.5 |
| 75 | 2015 | MST75 | TZEI1xTZEI19 | 2150.6 | 57.7 | 59.6 | 3.1 | 5.4 | 0.6 | 32.7 | 28.3 | 4.9 | 5.8 |
| 76 | 2015 | MST76 | TZE-WPopDTC5STRC5xTZEI1 | 3543.5 | 55.9 | 57.0 | 1.9 | 4.6 | 0.7 | 22.5 | 31.5 | 4.3 | 4.8 |
| 77 | 2015 | MST77 | TZE-WPopDTC5STRC5xTZEI3B | 3618.0 | 55.6 | 56.3 | 1.5 | 4.6 | 0.8 | 19.1 | 28.8 | 4.2 | 4.9 |
| 78 | 2015 | MST78 | TZE-WPopDTC5STRC5xTZEI68 | 2706.4 | 55.3 | 56.2 | 1.9 | 5.1 | 0.8 | 25.1 | 29.1 | 4.3 | 4.7 |
| 79 | 2015 | MST79 | TZE-WPopDTC5STRC5xTZEI80 | 3197.1 | 54.8 | 54.5 | 0.5 | 4.9 | 0.8 | 30.7 | 32.6 | 4.2 | 5.0 |
| 80 | 2015 | MST80 | TZE-WPopDTC5STRC5xTZEI31 | 2433.2 | 57.0 | 57.6 | 1.5 | 5.0 | 0.8 | 30.6 | 34.2 | 4.6 | 5.5 |
| 81 | 2015 | MST81 | TZE-WPopDTC5STRC5xTZEI56 | 3124.7 | 53.7 | 54.4 | 1.4 | 4.8 | 0.7 | 24.6 | 28.4 | 4.5 | 5.0 |
| 82 | 2015 | MST82 | TZE-WPopDTC5STRC5xTZEI18 | 2064.9 | 56.4 | 58.4 | 2.8 | 5.1 | 0.7 | 35.5 | 35.4 | 4.6 | 5.4 |
| 83 | 2015 | MST83 | TZE-WPopDTC5STRC5xTZEI19 | 1546.2 | 57.4 | 60.1 | 3.6 | 5.8 | 0.7 | 26.5 | 27.9 | 5.0 | 5.8 |
| 84 | 2015 | MST84 | TZE-WPopDTC5STRC5xTZEI7 | 2825.8 | 57.1 | 58.3 | 2.1 | 5.1 | 0.8 | 24.2 | 32.9 | 4.1 | 4.8 |
| 85 | 2015 | MST85 | TZE-WPopDTC5STRC5xTZEI63 | 2556.9 | 55.0 | 55.8 | 1.8 | 4.8 | 0.7 | 24.2 | 28.8 | 4.4 | 5.5 |
| 86 | 2015 | MST86 | TZE-WPopDTC5STRC5xENT11 | 4050.2 | 56.1 | 57.1 | 1.6 | 4.3 | 0.8 | 37.1 | 44.7 | 3.7 | 4.5 |
| 87 | 2015 | MST87 | TZE-WPopDTC5STRC5xENT12 | 3835.6 | 57.1 | 58.2 | 1.7 | 5.0 | 0.8 | 28.7 | 28.9 | 3.9 | 4.2 |
| 88 | 2016 | MST88 | TZEI86xTZEI60 | 2831.8 | 58.1 | 59.4 | 2.1 | 5.0 | 0.7 | 24.0 | 28.5 | 4.7 | 5.0 |
| 89 | 2016 | MST89 | TZEI56xTZEI32 | 2122.7 | 54.5 | 56.4 | 2.6 | 5.3 | 0.7 | 29.4 | 36.8 | 4.2 | 5.3 |
| 90 | 2016 | MST90 | TZEI48xTZEI63 | 1921.4 | 56.3 | 57.3 | 1.9 | 4.9 | 0.8 | 39.0 | 44.2 | 4.5 | 5.5 |
| 91 | 2016 | MST91 | TZdEI315xTZdEI124 | 2169.7 | 55.3 | 55.9 | 1.3 | 5.0 | 0.8 | 30.9 | 30.5 | 4.4 | 4.9 |
| 92 | 2016 | MST92 | TZdEI268xTZdEI131 | 3231.4 | 54.2 | 55.6 | 2.2 | 4.6 | 0.9 | 42.2 | 38.4 | 4.1 | 4.6 |
| 93 | 2016 | MST93 | TZdEI257xTZEI18 | 2926.7 | 57.8 | 59.1 | 2.0 | 5.2 | 0.8 | 23.0 | 23.4 | 4.1 | 4.4 |
| 94 | 2016 | MST94 | TZdEI98xTZdEI280 | 3984.9 | 54.0 | 54.5 | 1.5 | 4.1 | 0.8 | 26.6 | 37.0 | 3.6 | 4.3 |
| 95 | 2016 | MST95 | TZdEI268xTZEI105 | 3057.9 | 55.9 | 56.1 | 1.3 | 4.4 | 0.8 | 33.2 | 38.1 | 3.8 | 4.4 |
| 96 | 2016 | MST96 | TZEI281xTZEI18 | 2278.2 | 56.3 | 57.6 | 2.3 | 5.1 | 0.8 | 22.5 | 29.9 | 4.7 | 5.5 |
| 97 | 2016 | MST97 | TZEI242xTZEI31 | 1603.9 | 57.2 | 60.0 | 2.7 | 5.8 | 0.6 | 27.6 | 31.6 | 5.1 | 6.1 |
| 98 | 2016 | MST98 | TZEI240xTZEI31 | 1740.6 | 56.9 | 58.3 | 2.3 | 5.8 | 0.8 | 28.8 | 29.7 | 5.3 | 6.2 |
| 99 | 2016 | MST99 | TZEI281xTZEI31 | 2091.3 | 59.0 | 60.1 | 2.1 | 5.2 | 0.7 | 27.0 | 34.9 | 5.1 | 5.6 |
| 100 | 2016 | MST100 | ENT10xTZEI18 | 1356.7 | 60.0 | 60.5 | 1.2 | 5.8 | 0.7 | 23.0 | 37.9 | 4.2 | 5.5 |
| 101 | 2016 | MST101 | ENT10xTZEI7 | 2641.5 | 56.1 | 57.9 | 2.5 | 4.6 | 0.8 | 25.7 | 27.0 | 3.7 | 4.8 |
| 102 | 2017 | MST102 | TZEI326xTZdEI425 | 3184.2 | 57.7 | 58.3 | 1.7 | 4.9 | 0.8 | 21.2 | 30.7 | 3.8 | 4.5 |
| 103 | 2017 | MST103 | TZdEI352xTZEI383 | 3475.3 | 57.0 | 57.1 | 1.3 | 4.3 | 0.9 | 6.4 | 2.0 | 3.3 | 3.8 |
| 104 | 2017 | MST104 | TZdEI352xTZEI355 | 3731.2 | 56.0 | 55.8 | 0.8 | 3.7 | 0.9 | 21.8 | 18.6 | 3.2 | 3.8 |
| 105 | 2017 | MST105 | TZEI296xTZdEI352 | 3705.4 | 57.3 | 57.5 | 1.4 | 4.0 | 0.9 | 7.2 | 4.4 | 3.2 | 3.9 |
| 106 | 2017 | MST106 | TZEI7xTZdEI352 | 3419.7 | 55.3 | 56.7 | 2.7 | 4.2 | 0.9 | 28.3 | 22.9 | 3.3 | 4.1 |
| 107 | 2017 | MST107 | TZdEI120xTZEI383 | 3703.0 | 52.6 | 52.9 | 1.6 | 4.0 | 0.9 | 9.4 | 3.7 | 3.3 | 4.6 |
| 108 | 2017 | MST108 | TZdEI173xTZdEI352 | 3211.7 | 56.9 | 56.6 | 0.6 | 4.4 | 1.0 | 21.6 | 18.9 | 3.2 | 3.9 |
| 109 | 2017 | MST109 | TZdEI479xTZdEI260 | 3279.7 | 54.4 | 54.3 | 1.1 | 4.9 | 0.9 | 8.9 | 10.8 | 4.5 | 5.8 |
| 110 | 2017 | MST110 | TZdEI98xTZdEI492 | 3302.6 | 57.2 | 58.2 | 2.4 | 4.5 | 0.9 | 41.0 | 37.6 | 4.1 | 4.7 |
| 111 | 2018 | MST111 | DTESTR-WSynPopC4xTZdEI173 | 3430.1 | 56.1 | 57.1 | 2.2 | 4.7 | 0.8 | 34.0 | 31.8 | 4.0 | 4.7 |
| 112 | 2018 | MST112 | DTESTR-WSynPopC4xTZdEI492 | 3182.3 | 56.6 | 58.4 | 2.4 | 4.6 | 0.8 | 35.8 | 45.8 | 3.9 | 4.7 |
| 113 | 2018 | MST113 | DTESTR-WSynPopC4xTZdEI98 | 2631.8 | 55.2 | 55.5 | 1.8 | 5.0 | 0.7 | 40.5 | 38.6 | 4.4 | 5.7 |
| 114 | 2018 | MST114 | TZE-WPopDTC5STRC5xTZdEI120 | 3031.6 | 54.4 | 56.2 | 2.4 | 4.4 | 0.8 | 26.1 | 27.3 | 3.6 | 4.9 |
| 115 | 2018 | MST115 | TZEI1207xTZdEI352 | 3088.2 | 56.8 | 57.4 | 2.0 | 3.7 | 0.9 | 22.4 | 28.2 | 3.2 | 4.1 |
| 116 | 2018 | MST116 | TZdEI173xTZdEI280 | 4287.0 | 54.6 | 54.8 | 1.8 | 3.5 | 1.0 | 22.9 | 28.7 | 2.8 | 3.5 |
| 117 | 2018 | MST117 | TZdEI71xTZdEI268 | 3177.5 | 54.7 | 55.9 | 2.3 | 4.5 | 0.8 | 26.7 | 34.0 | 3.5 | 4.6 |
| 118 | 2018 | MST118 | TZdEI260xTZdEI396 | 3735.4 | 56.1 | 56.0 | 2.2 | 4.9 | 0.9 | 25.4 | 25.6 | 3.6 | 5.0 |
| 119 | 2018 | MST119 | TZdEI173xTZdEI492 | 3733.4 | 56.0 | 57.1 | 2.5 | 4.2 | 0.9 | 25.3 | 28.2 | 3.2 | 3.8 |
| 120 | 2018 | MST120 | TZEI1348xTZdEI352 | 3200.0 | 57.7 | 59.6 | 2.9 | 4.4 | 0.9 | 19.0 | 21.9 | 3.3 | 3.8 |
| 121 | 2018 | MST121 | TZEI1238xTZdEI352 | 3157.9 | 57.9 | 59.7 | 2.6 | 4.1 | 0.8 | 26.0 | 29.7 | 3.3 | 3.9 |
| 122 | 2018 | MST122 | TZEI1344xTZdEI352 | 3072.1 | 56.6 | 57.3 | 2.3 | 4.1 | 0.9 | 20.0 | 22.6 | 3.6 | 4.3 |
| 123 | 2018 | MST123 | TZEI1305xTZdEI352 | 3037.6 | 57.9 | 59.0 | 2.2 | 3.9 | 0.9 | 22.7 | 26.7 | 3.4 | 4.0 |
| 124 | 2018 | MST124 | TZEI1323xTZEI18 | 3123.0 | 56.7 | 57.6 | 2.2 | 4.9 | 0.8 | 30.9 | 28.5 | 4.0 | 4.9 |
| 125 | 2018 | MST125 | TZEI1252xTZEI7 | 3448.5 | 54.8 | 55.5 | 2.0 | 4.6 | 0.9 | 27.9 | 26.9 | 3.3 | 4.5 |
| 126 | 2018 | MST126 | TZdEI268xTZEI31 | 3147.6 | 55.5 | 56.9 | 2.6 | 5.2 | 0.9 | 42.6 | 34.7 | 4.1 | 5.2 |
| 127 | 2018 | MST127 | TZEI361xTZEI7 | 2942.2 | 55.8 | 57.3 | 2.4 | 4.6 | 0.8 | 25.6 | 27.3 | 3.6 | 4.7 |
| 128 | 2018 | MST128 | TZdEI100xTZdEI352 | 3021.4 | 56.7 | 57.8 | 2.3 | 4.1 | 0.9 | 22.2 | 25.9 | 3.4 | 3.9 |
| 129 | 2018 | MST129 | TZE-WPopDTC5STRC5xTZEI296 | 2860.1 | 57.3 | 58.8 | 2.4 | 4.7 | 0.8 | 27.4 | 31.2 | 3.8 | 4.7 |
| 130 | 2018 | MST130 | TZE-WPopDTC5STRC5xTZEI383 | 3149.7 | 56.5 | 57.7 | 2.0 | 3.4 | 0.8 | 26.3 | 30.4 | 4.1 | 4.5 |
| 131 | 2019 | MST131 | TZEI375xTZdEI352 | 3041.0 | 58.4 | 59.7 | 2.3 | 4.5 | 0.8 | 35.0 | 36.2 | 3.5 | 3.9 |
| 132 | 2019 | MST132 | TZdEI441xTZEI18 | 2773.5 | 58.4 | 60.2 | 2.6 | 4.7 | 0.8 | 31.3 | 34.5 | 4.0 | 5.0 |
| 133 | 2019 | MST133 | TZdEI260xTZdEI352 | 2737.8 | 57.5 | 58.0 | 1.9 | 4.8 | 0.8 | 23.3 | 25.6 | 3.8 | 4.4 |
| 134 | 2019 | MST134 | TZEI387xTZdEI352 | 3156.1 | 57.7 | 58.8 | 2.2 | 4.1 | 0.9 | 24.0 | 24.3 | 3.1 | 3.7 |
| 135 | 2019 | MST135 | TZEI361xTZEI19 | 2730.4 | 56.0 | 58.3 | 3.2 | 5.5 | 0.8 | 30.2 | 37.0 | 4.4 | 5.4 |
| 136 | 2019 | MST136 | TZdEI260xTZEI7 | 3810.1 | 54.6 | 55.5 | 2.0 | 4.8 | 0.9 | 34.2 | 35.1 | 3.7 | 4.8 |
| 137 | 2019 | MST137 | TZEI385xTZdEI352 | 2817.7 | 57.4 | 58.1 | 2.3 | 4.0 | 0.9 | 22.0 | 25.9 | 3.3 | 3.7 |
| 138 | 2019 | MST138 | TZEI968xTZdEI352 | 3413.3 | 57.7 | 58.6 | 2.6 | 4.8 | 0.8 | 44.7 | 36.9 | 3.9 | 5.0 |
| 139 | 2019 | MST139 | TZdEI485xTZdEI352 | 3194.8 | 56.5 | 58.4 | 2.8 | 4.1 | 0.8 | 23.1 | 24.2 | 2.8 | 3.8 |
| 140 | 2019 | MST140 | (TZdEI352xTZdEI260)xTZdEI551 | 3502.0 | 56.2 | 57.0 | 2.3 | 4.2 | 0.9 | 15.9 | 21.0 | 3.1 | 3.7 |
| 141 | 2019 | MST141 | (TZdEI352xTZdEI260)xTZEI296 | 3306.8 | 55.8 | 57.0 | 2.6 | 4.5 | 0.9 | 25.3 | 27.1 | 3.9 | 4.9 |
| 142 | 2020 | MST142 | (TZdEI352xTZdEI260)xTZdEI441 | 2759.9 | 57.5 | 58.5 | 2.7 | 4.8 | 0.9 | 19.6 | 23.8 | 3.6 | 4.7 |
| 143 | 2020 | MST143 | TZEI1348xTZEI18 | 3243.8 | 56.7 | 58.6 | 2.3 | 4.3 | 0.9 | 29.7 | 36.1 | 3.2 | 4.5 |
| 144 | 2020 | MST144 | TZEI1426xTZdEI352 | 3016.4 | 58.2 | 59.5 | 2.4 | 4.2 | 0.9 | 24.0 | 24.8 | 4.0 | 4.5 |
| 145 | 2020 | MST145 | TZEI75xTZEI32 | 3237.0 | 56.6 | 57.3 | 2.3 | 5.0 | 0.8 | 32.6 | 37.4 | 4.0 | 4.6 |
| 146 | 2020 | MST146 | TZEI1493xTZdEI352 | 2940.5 | 57.1 | 58.1 | 2.3 | 4.6 | 0.9 | 27.4 | 33.3 | 3.6 | 4.5 |
| 147 | 2020 | MST147 | TZEI1361xTZdEI100 | 2900.0 | 55.8 | 57.5 | 3.2 | 4.9 | 0.8 | 22.4 | 24.8 | 3.9 | 5.1 |
| 148 | 2020 | MST148 | TZEI771xTZdEI352 | 2920.2 | 59.6 | 59.9 | 2.6 | 4.6 | 0.8 | 28.0 | 26.4 | 3.9 | 4.5 |
| 149 | 2020 | MST149 | TZEI5xTZEI75 | 2050.3 | 57.3 | 58.6 | 2.6 | 5.4 | 0.6 | 45.0 | 45.7 | 5.2 | 6.6 |
| 150 | 2020 | MST150 | TZEI1378xTZdEI352 | 3663.3 | 55.2 | 56.9 | 2.4 | 4.2 | 0.9 | 31.3 | 37.4 | 3.2 | 3.9 |
| 151 | 2020 | MST151 | TZEI32xTZEI5 | 2093.0 | 56.3 | 57.9 | 3.7 | 5.4 | 0.7 | 42.6 | 37.0 | 5.3 | 6.4 |
| 152 | 2020 | MST152 | TZEI60xTZEI89 | 2281.2 | 59.2 | 60.3 | 2.7 | 5.5 | 0.7 | 27.6 | 26.8 | 4.4 | 5.2 |
| 153 | 2020 | MST153 | TZEI1379xTZdEI352 | 3114.2 | 56.5 | 58.9 | 3.1 | 4.5 | 0.8 | 25.1 | 32.5 | 3.6 | 4.5 |
| 154 | 2020 | MST154 | TZEI1419xTZEI7 | 2048.2 | 57.5 | 58.6 | 2.8 | 5.3 | 0.7 | 31.5 | 50.3 | 4.8 | 6.0 |
| 155 | 2021 | MST155 | TZEI1xTZEI75 | 2938.7 | 56.0 | 57.5 | 2.6 | 4.7 | 0.9 | 30.7 | 33.3 | 3.8 | 4.8 |
| 156 | 2021 | MST156 | TZEI1241xTZdEI352 | 3433.0 | 58.9 | 59.7 | 2.7 | 3.9 | 0.8 | 16.7 | 18.2 | 3.7 | 3.4 |
| 157 | 2021 | MST157 | TZEI1429xTZdEI100 | 2775.0 | 55.7 | 56.7 | 2.3 | 4.8 | 0.9 | 38.3 | 37.3 | 3.7 | 5.1 |
| 158 | 2021 | MST158 | TZEI1386xTZdEI100 | 3053.5 | 54.2 | 55.0 | 2.1 | 4.6 | 0.8 | 30.9 | 31.8 | 3.9 | 5.0 |
| 159 | 2021 | MST159 | TZEI5xTZEI40 | 2488.8 | 56.9 | 58.7 | 2.6 | 5.2 | 0.7 | 46.5 | 47.0 | 4.4 | 5.5 |
| 160 | 2012 | LOC1 | Ife hybrid6 | 2644.0 | 55.0 | 57.4 | 2.6 | 5.5 | 0.8 | 30.6 | 27.7 | 4.1 | 4.9 |
| 161 | 2011 | LOC2 | Suhudoo | 1913.9 | 56.3 | 58.7 | 2.6 | 5.4 | 0.7 | 35.1 | 40.8 | 5.4 | 6.1 |
| 162 | 2014 | LOC3 | SAMMAZ47 | 2382.6 | 54.4 | 56.2 | 2.6 | 5.3 | 0.8 | 27.3 | 28.1 | 4.5 | 5.4 |
| 163 | 2010 | LOC4 | SAMMAZ27 | 2346.7 | 56.3 | 57.4 | 2.3 | 4.9 | 0.7 | 44.7 | 40.3 | 4.3 | 5.1 |
| 164 | 2013 | LOC5 | SAMMAZ46 | 2187.0 | 56.0 | 59.9 | 2.2 | 4.9 | 0.7 | 26.3 | 30.0 | 5.5 | 5.9 |
| 165 |  | SCO1 | SC419 | 2964.6 | 58.2 | 59.6 | 2.5 | 5.6 | 0.7 | 32.3 | 33.5 | 4.5 | 5.1 |
| 166 |  | SCO2 | 11C82 | 2774.9 | 56.0 | 57.0 | 2.0 | 4.9 | 0.7 | 33.9 | 43.3 | 5.2 | 5.5 |
| 167 |  | SCO3 | 06C4095 | 2756.6 | 58.7 | 59.7 | 1.9 | 5.6 | 0.7 | 33.6 | 29.3 | 5.3 | 5.5 |
| 168 |  | SCO4 | 10C8449 | 2743.0 | 57.4 | 59.2 | 2.7 | 5.2 | 0.6 | 33.2 | 32.5 | 4.4 | 5.6 |
| 169 |  | SCO5 | SC403 | 2682.8 | 56.2 | 58.7 | 3.5 | 5.5 | 0.7 | 23.1 | 25.2 | 4.3 | 5.2 |
| 170 |  | SCO6 | 10C9811 | 2473.7 | 56.2 | 58.8 | 3.4 | 5.4 | 0.7 | 38.2 | 44.0 | 4.6 | 5.5 |
| 171 |  | SCO7 | 10C4108 | 2178.8 | 58.2 | 63.4 | 6.2 | 5.0 | 0.3 | 56.8 | 90.9 | 4.7 | 5.4 |
| 172 |  | SCO8 | SC555 | 2091.1 | 59.5 | 61.2 | 3.2 | 6.2 | 0.6 | 48.2 | 44.0 | 4.1 | 5.3 |
| 173 |  | SCO9 | 10C8447 | 2079.1 | 57.9 | 59.9 | 2.7 | 5.5 | 0.6 | 42.4 | 40.9 | 4.8 | 6.0 |
| 174 |  | SCO10 | SC535 | 1980.2 | 58.4 | 60.6 | 3.2 | 5.9 | 0.5 | 38.0 | 46.3 | 5.8 | 6.4 |
| 175 |  | SCO11 | SC533 | 1850.7 | 58.5 | 61.0 | 3.2 | 6.2 | 0.6 | 25.3 | 31.3 | 5.3 | 6.1 |
| 176 |  | SCO12 | SC529 | 1114.6 | 58.4 | 60.9 | 3.5 | 5.6 | 0.7 | 27.6 | 33.3 | 4.0 | 4.6 |
| 177 |  | SCO13 | 07C3174 | 964.7 | 58.5 | 63.1 | 5.4 | 6.7 | 0.4 | 33.0 | 46.3 | 5.9 | 6.3 |

Table S2: Description of test locations used for evaluation of early maize hybrids under STRINF condition in Nigeria, 2010 –2021

| Location | Abuja | Mokwa |
| --- | --- | --- |
| Agro-ecology | Southern Guinea Savanna | Southern Guinea Savanna |
| Latitude | 9°40'N | 9°18’N |
| Longitude | 7° 29'E | 5°4’E |
| Altitude (m) ASL | 360 | 457 |
| Soil type | Lixisol | Lixisol |
| Average annual rainfall (mm) | 1389 | 1100 |
| Average temperature (°C) | 25.7 | 27.5 |
