# Supplementary material for: Repeatability and genetic advances in early maturing maize hybrid trials conducted under Striga-infested and non-infested conditions
Source: PLoS One. 2025 Apr 3;20(4):e0319353. doi: 10.1371/journal.pone.0319353 (PMC11967925; doi:10.1371/journal.pone.0319353)
Supplement: Attachment — Submitted filename: PONE-D-24-47860_R1_reviewer.pdf [file pone.0319353.s004.pdf]

# PLOS ONE

# REPEATABILITY AND GENETIC ADVANCEMENT IN EARLY MATURING MAIZE HYBRID TRIALS CONDUCTED UNDER STRIGA-INFESTED AND NON-INFESTED CONDITIONS

--Manuscript Draft--

| | |
|---|---|
| **Manuscript Number:** | PONE-D-24-47860R1 |
| **Article Type:** | Research Article |
| **Full Title:** | REPEATABILITY AND GENETIC ADVANCEMENT IN EARLY MATURING MAIZE HYBRID TRIALS CONDUCTED UNDER STRIGA-INFESTED AND NON-INFESTED CONDITIONS |
| **Short Title:** | GENETIC ADVANCEMENT IN EARLY MATURING MAIZE |
| **Corresponding Author:** | Idris Ishola Adejumobi, Ph.D.<br>International Institute of Tropical Agriculture<br>Ibadan, Oyo NIGERIA |
| **Keywords:** | selection; genetic gains; Striga resistance; grain yield; cereal crops |
| **Abstract:** | In sub-Saharan Africa (SSA), maize (Zea mays L.) is both a cash crop and an important staple crop. However, Strigahermonthica infection constrains its production and productivity. A total of 159 hybrids from 21 international trials were evaluated under Striga-infested (STRINF) and Striga non-infested (STRNON) conditions at Mokwa and Abuja, Nigeria, from 2010 to 2021. The data were used to (i) determine the genetic enhancements in grain yield and Striga adaptive traits and (ii) assess the repeatability of the trials in identification of promising hybrids. Significant annual genetic gains in grain yield of 3.40% and 3.71% with increases of 76.87 and 127.02 kg ha−1 yr−1 were recorded under STRINF and STRNON conditions, respectively. The genetic gains in grain yield were associated with 3.04 and 2.25 % decreases in Striga damage ratings at 8 and 10 weeks after planting (WAP), respectively, and 1.27 % in the number of emerged Striga plants at 10 WAP. The results indicated that ears per plant and flowering dates had the highest repeatability estimates while the number of emerged Striga plants at 8 and 10 WAP recorded the lowest repeatability. Generally, substantial progress has been achieved with a good level of repeatability estimates for the early maturing maize hybrid trials evaluated under STRINF and STRNON conditions. Those results have demonstrated that the breeding strategies adopted during the 12-year breeding period have been effective, and that the potential of the trials in the exploration of the genetic potential of the hybrids for commercialization in the SSA for food security and poverty alleviation. |
| **Order of Authors:** | Adamu Masari Abubakar, MSc |
| | Idris Ishola Adejumobi, Ph.D. |
| | Kayode Rapheal Fowobaje, MSc |
| | Ibnou Dieng, Ph.D |
| | Zakariya Saminu, Ph.D |
| | Simon Imoro, MSc |
| | Baffour Badu-Apraku, PhD |
| **Response to Reviewers:** | REBUTTAL TO REVIEWERS' COMMENTS ON THE MANUSCRIPT TITLED " REPEATABILITY AND GENETIC ADVANCES IN EARLY MATURING MAIZE HYBRID TRIALS CONDUCTED UNDER STRIGA-INFESTED AND NON-INFESTED CONDITIONS"<br>Response to Editor (Journal requirements)<br>1.Please ensure that your manuscript meets PLOS ONE's style requirements, including those for file naming.<br><br>Response: The manuscript has been modified to meet PLOS ONE's style requirements. |

*Powered by Editorial Manager® and ProduXion Manager® from Aries Systems Corporation*

2.Please note that funding information should not appear in any section or other areas of your manuscript. We will only publish funding information present in the Funding Statement section of the online submission form. Please remove any funding-related text from the manuscript.

Response: The funding-related text has been removed from the manuscript

3.We note that the grant information you provided in the 'Funding Information' and 'Financial Disclosure' sections do not match. When you resubmit, please ensure that you provide the correct grant numbers for the awards you received for your study in the 'Funding Information' section.

Response: The funding information will be corrected upon resubmission of the revised manuscript.

4.Thank you for stating the following financial disclosure: "This research was supported by the Bill & Melinda Gates Foundation under the Accelerated Genetic Gains (AGG) and Stress Tolerant Maize for Africa (STMA) Projects."
Please state what role the funders took in the study. If the funders had no role, please state: "The funders had no role in study design, data collection and analysis, decision to publish, or preparation of the manuscript." If this statement is not correct you must amend it as needed. Please include this amended Role of Funder statement in your cover letter; we will change the online submission form on your behalf.

Response: The funder was the main donor of the AGG and STMA projects and also the payer of the article processing charge for the manuscript.

5.Thank you for uploading your study's underlying data set. Unfortunately, the repository you have noted in your Data Availability statement does not qualify as an acceptable data repository according to PLOS's standards. At this time, please upload the minimal data set necessary to replicate your study's findings to a stable, public repository (such as figshare or Dryad) and provide us with the relevant URLs, DOIs, or accession numbers that may be used to access these data. For a list of recommended repositories and additional information on PLOS standards for data deposition, please see https://journals.plos.org/plosone/s/recommended-repositories

Response: The acceptable data repository according to PLOS's standards with DOIs have now been provided in the data availability statement of the manuscript

6.When completing the data availability statement of the submission form, you indicated that you will make your data available on acceptance. We strongly recommend all authors decide on a data sharing plan before acceptance, as the process can be lengthy and hold up publication timelines. Please note that, though access restrictions are acceptable now, your entire data will need to be made freely accessible if your manuscript is accepted for publication. This policy applies to all data except where public deposition would breach compliance with the protocol approved by your research ethics board. If you are unable to adhere to our open data policy, please kindly revise your statement to explain your reasoning and we will seek the editor's input on an exemption. Please be assured that, once you have provided your new statement, the assessment of your exemption will not hold up the peer review process.

Response: The data have now been made available.

7.We note you have included a table to which you do not refer in the text of your manuscript. Please ensure that you refer to Table 1 in your text; if accepted, production will need this reference to link the reader to the Table.

Response: We have made appropriate reference to Table 1 in the revised version of the manuscript

8.Please include captions for your Supporting Information files at the end of your manuscript, and update any in-text citations to match accordingly. Please see our Supporting Information guidelines for more information:

http://journals.plos.org/plosone/s/supporting-information.

Response: Captions for Supporting Information files have now been provided at the end of the manuscript

9.Please review your reference list to ensure that it is complete and correct. If you have cited papers that have been retracted, please include the rationale for doing so in the manuscript text, or remove these references and replace them with relevant current references. Any changes to the reference list should be mentioned in the rebuttal letter that accompanies your revised manuscript. If you need to cite a retracted article, indicate the article's retracted status in the References list and also include a citation and full reference for the retraction notice.

Response: The list of references has been reviewed to ensure that it is complete and correct. We observed that a reference was not cited appropriately which was taken care of. In addition, duplicate references have also been resolved in the revised version of the manuscript.

Responses to Reviewers' Comments
Reviewer #1: The quality of the manuscript is technically sound, and data addressed all objectives. However, some concerns are:

1. Results should be reported in the past tense.

Response: The results section of the manuscript has been reported in the past tense as requested.

2. Some minor grammatical errors need correction; a few have already been pointed out.

Response: We have revised the manuscript for grammatical modifications and sentence chronology to improve readability in the revised version of the manuscript as requested.

3. Figures addressing Objective 2 are missing.

Response: We apologize for this however all figures relevant to the work were submitted separately to the PLOS ONE webpage. We hope the editor can resolve this issue in the subsequent interactive review.

4. Please refer to Table 3 and recast your results on repeatability values for the traits.

Response: As requested, we have recast the results relating to Table 3 to ensure accurate interpretation for readers.

5. Indicate in the Materials and Methods section the method used for assessing the significance of values.

Response: The method used for assessing the significance of the values have been included in the Materials and Methods section as requested in the revised manuscript.

6. Title can be rephrased as "Repeatability and genetic gain"

Response: We appreciate your suggestion to improve the quality of the manuscript. The co-authors have several similar publications with titles including the word "genetic gains". We would like to use the word "genetic advances" as a replacement for "genetic advancement" to provide a sense of uniqueness to the manuscript title.

7. Restructure your conclusions to address the objectives.
Response: We have restructured the conclusion section to focus on the objectives in the revised manuscript as requested.
Reviewer #2: The manuscript is well written except for few MINOR REVISIONS to be made before acceptance:

Powered by Editorial Manager® and ProduXion Manager® from Aries Systems Corporation

| | |
|---|---|
| | (1) The 'Introduction' section is too long and need to be shortened with good flow of the required information<br>Response: Thank you. We have revised the Introduction as requested.<br><br>(2) Correct as 'Materials and Methods'<br>Response: Material and Method were corrected as 'Materials and Methods' as requested.<br><br>(3) Present the coordinates of the test locations<br>Response: Coordinates of the test locations have been referenced in the revised manuscript as requested.<br><br>(4) Present the agro-climatic pattern of the different locations and years<br>Response: Thank you for your comment. The agro-climatic patterns of the two locations have been included as a supplementary file to be submitted alongside the revised manuscript.<br><br>(5) The discussion to be presented with suitable sub-headings for easy understanding to the readers<br>Response: Discussion has now been presented with suitable sub-headings as requested. |
| **Additional Information:** | |
| **Question** | **Response** |
| **Financial Disclosure**<br><br>Enter a financial disclosure statement that describes the sources of funding for the work included in this submission. Review the submission guidelines for detailed requirements. View published research articles from *PLOS ONE* for specific examples.<br><br>This statement is required for submission and **will appear in the published article** if the submission is accepted. Please make sure it is accurate.<br><br>**Funded studies**<br>Enter a statement with the following details:<br>• Initials of the authors who received each award<br>• Grant numbers awarded to each author<br>• The full name of each funder<br>• URL of each funder website<br>• Did the sponsors or funders play any role in the study design, data collection and analysis, decision to publish, or preparation of the manuscript? | Yes |

| | |
|---|---|
| Did you receive funding for this work? | |
| Please add funding details.<br>as follow-up to "**Financial Disclosure**<br><br>Enter a financial disclosure statement that describes the sources of funding for the work included in this submission. Review the submission guidelines for detailed requirements. View published research articles from *PLOS ONE* for specific examples.<br><br>This statement is required for submission and **will appear in the published article** if the submission is accepted. Please make sure it is accurate.<br><br>**Funded studies**<br>Enter a statement with the following details:<br>• Initials of the authors who received each award<br>• Grant numbers awarded to each author<br>• The full name of each funder<br>• URL of each funder website<br>• Did the sponsors or funders play any role in the study design, data collection and analysis, decision to publish, or preparation of the manuscript?<br><br>Did you receive funding for this work?" | This research was supported by the Bill & Melinda Gates Foundation under the Accelerated Genetic Gains (AGG) and Stress Tolerant Maize for Africa (STMA) Projects. The funder was the main donor of the AGG and STMA projects and also the payer of the article processing charge for the manuscript. |
| Please select the country of your main research funder (please select carefully as in some cases this is used in fee calculation).<br>as follow-up to "**Financial Disclosure**<br><br>Enter a financial disclosure statement that describes the sources of funding for the work included in this submission. Review the submission guidelines for detailed requirements. View published research articles from *PLOS ONE* for specific examples.<br><br>This statement is required for submission and **will appear in the published article** if the submission is accepted. Please make | UNITED STATES - US |

| | |
|---|---|
| sure it is accurate.<br><br>**Funded studies**<br>Enter a statement with the following details:<br>• Initials of the authors who received each award<br>• Grant numbers awarded to each author<br>• The full name of each funder<br>• URL of each funder website<br>• Did the sponsors or funders play any role in the study design, data collection and analysis, decision to publish, or preparation of the manuscript?<br><br>Did you receive funding for this work?" | |
| **Competing Interests**<br><br>Use the instructions below to enter a competing interest statement for this submission. On behalf of all authors, disclose any competing interests that could be perceived to bias this work—acknowledging all financial support and any other relevant financial or non-financial competing interests.<br><br>This statement is required for submission and **will appear in the published article** if the submission is accepted. Please make sure it is accurate and that any funding sources listed in your Funding Information later in the submission form are also declared in your Financial Disclosure statement.<br><br>View published research articles from *PLOS ONE* for specific examples. | The authors have declared that no competing interests exist. |

| | |
|---|---|
| **NO authors have competing interests**<br><br>Enter: *The authors have declared that no competing interests exist.*<br><br>**Authors with competing interests**<br><br>Enter competing interest details beginning with this statement:<br><br>*I have read the journal's policy and the authors of this manuscript have the following competing interests: [insert competing interests here]*<br><br>* typeset | |
| **Ethics Statement**<br><br>Enter an ethics statement for this submission. This statement is required if the study involved:<br><br>• Human participants<br>• Human specimens or tissue<br>• Vertebrate animals or cephalopods<br>• Vertebrate embryos or tissues<br>• Field research<br><br>Write "N/A" if the submission does not require an ethics statement.<br><br>General guidance is provided below. Consult the submission guidelines for detailed instructions. **Make sure that all information entered here is included in the Methods section of the manuscript.** | NA |

| | |
|---|---|
| **Format for specific study types**<br><br>**Human Subject Research (involving human participants and/or tissue)**<br>• Give the name of the institutional review board or ethics committee that approved the study<br>• Include the approval number and/or a statement indicating approval of this research<br>• Indicate the form of consent obtained (written/oral) or the reason that consent was not obtained (e.g. the data were analyzed anonymously)<br><br>**Animal Research (involving vertebrate animals, embryos or tissues)**<br>• Provide the name of the Institutional Animal Care and Use Committee (IACUC) or other relevant ethics board that reviewed the study protocol, and indicate whether they approved this research or granted a formal waiver of ethical approval<br>• Include an approval number if one was obtained<br>• If the study involved *non-human primates*, add *additional details* about animal welfare and steps taken to ameliorate suffering<br>• If anesthesia, euthanasia, or any kind of animal sacrifice is part of the study, include briefly which substances and/or methods were applied<br><br>**Field Research**<br><br>Include the following details if this study involves the collection of plant, animal, or other materials from a natural setting:<br>• Field permit number<br>• Name of the institution or relevant body that granted permission | |
| **Data Availability**<br><br>Authors are required to make all data underlying the findings described fully available, without restriction, and from the time of publication. PLOS allows rare exceptions to address legal and ethical concerns. See the PLOS Data Policy and FAQ for detailed information. | Yes - all data are fully available without restriction |

| | |
|---|---|
| A Data Availability Statement describing where the data can be found is required at submission. Your answers to this question constitute the Data Availability Statement and **will be published in the article**, if accepted.<br><br>**Important:** Stating 'data available on request from the author' is not sufficient. If your data are only available upon request, select 'No' for the first question and explain your exceptional situation in the text box.<br><br>Do the authors confirm that all data underlying the findings described in their manuscript are fully available without restriction? | |
| **Describe where the data may be found in full sentences. If you are copying our sample text, replace any instances of XXX with the appropriate details.**<br><br>• If the data are **held or will be held in a public repository**, include URLs, accession numbers or DOIs. If this information will only be available after acceptance, indicate this by ticking the box below. For example: *All XXX files are available from the XXX database (accession number(s) XXX, XXX.).*<br>• If the data are all contained **within the manuscript and/or Supporting Information files**, enter the following: *All relevant data are within the manuscript and its Supporting Information files.*<br>• If neither of these applies but you are able to provide **details of access elsewhere**, with or without limitations, please do so. For example:<br><br>*Data cannot be shared publicly because of [XXX]. Data are available from the XXX Institutional Data Access / Ethics Committee (contact via XXX) for researchers who meet the criteria for access to confidential data.*<br><br>*The data underlying the results presented in the study are available from (include the name of the third party* | The raw data used/analyzed in this manuscript were available in IITA CKAN repository (https://doi.org/10.25502/8a4f-h291/d) |

| | |
|---|---|
| *and contact information or URL).*<br>• This text is appropriate if the data are owned by a third party and authors do not have permission to share the data.<br><br>* typeset | |
| Additional data availability information: | Tick here if the URLs/accession numbers/DOIs will be available only after acceptance of the manuscript for publication so that we can ensure their inclusion before publication. |

Powered by Editorial Manager® and ProduXion Manager® from Aries Systems Corporation

Manuscript Click here to access/download;Manuscript;Manuscript.docx

# Repeatability and genetic advances in early maturing maize hybrid trials conducted under *Striga*-infested and non-infested conditions

Adamu Masari Abubakar[1,2], Idris Ishola Adejumobi[1*], Kayode Rapheal Fowobaje[1], Ibnou Dieng[1], Saminu Zakariya[2], Simon Imoro[1] and Baffour Badu-Apraku[1]

[1]International Institute of Tropical Agriculture (IITA), PMB 5320, Oyo Road, Ibadan, Oyo State, Nigeria; a.abubakar@cgiar.org (A.A.M.); i.adejumobi@cgiar.org (I.I.A.); k.fowobaje@cgiar.org (K.R.F.); i.dieng@cgiar.org (I.D.); s.imoro@cgiar.org (S.I); b.badu-apraku@cgiar.org (B.B.A.)

[2]Department of Plant Science, Institute for Agricultural Research, Ahmadu Bello University, P.M.B. 104, Zaria, Kaduna State, Nigeria. zacqkz@gmail.com (S.Z.)

* Corresponding author

E-mail: i.adejumobi@cgiar.org (I.I.A.)

## Abstract

In sub-Saharan Africa (SSA), maize (*Zea mays* L.) is both a cash crop and an important staple crop. However, *Striga hermonthica* infection constrains its production and productivity. A total of 159 hybrids from 21 international trials were evaluated under *Striga*-infested (STRINF) and *Striga* non-infested (STRNON) conditions at Mokwa and Abuja, Nigeria, from 2010 to 2021. The data were used to (i) determine the genetic

enhancements in grain yield and *Striga* adaptive traits and (ii) assess the repeatability of the trials in identification of promising hybrids. Significant annual genetic gains in grain yield of 3.40% and 3.71% with increases of 76.87 and 127.02 kg $ha^{-1}$ $yr^{-1}$ were recorded under STRINF and STRNON conditions, respectively. The genetic gains in grain yield were associated with 3.04 and 2.25 % decreases in *Striga* damage ratings at 8 and 10 weeks after planting (WAP), respectively, and 1.27 % in the number of emerged *Striga* plants at 10 WAP. The results indicated that ears per plant and flowering dates had the highest repeatability estimates while the number of emerged *Striga* plants at 8 and 10 WAP recorded the lowest repeatability. Generally, substantial progress has been achieved with a good level of repeatability estimates for the early maturing maize hybrid trials evaluated under STRINF and STRNON conditions.Those results have demonstrated that the breeding strategies adopted during the 12-year breeding period have been effective, and that the potential of the trials in the exploration of the genetic potential of the hybrids for commercialization in the SSA for food security and poverty alleviation.

**Keywords**: selection, genetic gains, *Striga* resistance, grain yield, cereal crops.

# Introduction

Maize plays an important role as a staple food in the diets of millions of people in Africa, especially in ensuring food security. Maize production and productivity are faced by a myriad constraints of which *Striga hermonthica* (purple witchweed), an obligate hemi-parasitic plant is one of the major factors causing substantial loss to grain yield on farmers' fields in sub-Saharan Africa (SSA) [1] . Even though *S. hermonthica* parasitizes the root systems of several cereals, maize is the most preferred among staple cereal crops. Every

cropping season, soil-borne *Striga* seeds germinate and attach to maize plant root systems, causing plant toxicity and yield reduction by siphoning water and nutrients from the host maize plant, resulting in the death of the host plant in extreme cases. The outcome of this association is a significant reduction in the obtainable grain yield. *Striga* parasitism is more common in the Savanna of SSA resulting in food insecurity and rural poverty. The lifespan of *Striga* depends on its ability to siphon nutrients and water from host plants to promote its growth and development. *Striga* species have 5-7 days to establish themselves on a suitable host, otherwise, if the stored resource in the seeds are exhausted, the radicles will wither [2] *Striga* is a major threat cereals crops. It causes yield losses of $7 to $10 billion worth and, could result in up to 100% losses in maize production [3–6]

Breeding for *Striga* resistance has proven to be the most cost-effective, feasible, and long-term strategy for control by farmers with limited resources. The scientists of the International Institute of Tropical Maize Improvement Program (IITA-MIP) initiated *Striga* resistance research on maize in 1982 with most of the germplasm originating from the U.S. Corn Belt. Resistant inbreds and hybrids of IITA were first identified in 1983 from field testing at Mokwa in Nigeria [7]. Since then, breeders of the Maize Improvement Program (MIP) have been working steadily to develop maize varieties/hybrids with elevated levels of *Striga* tolerance to support smallholder farmers in the targeted production environments. In this regard, IITA-MIP in collaboration with the National Agricultural Research Scientist (NARS) through deliberate teamwork, has developed and released several high-yielding multiple stress-tolerant (MST) (i.e., resistant to *Striga* and tolerant to drought and low N) hybrids and open-pollinated varieties (OPVs), many of which are suitable for several agro-ecologies in SSA. For the past two decades, the IITA-MIP has been developing and deploying *Striga*-tolerant hybrids through regional trials to NARS in West

and Central Africa (WCA) for evaluation and commercialization in the sub-region. The hybrids in the regional trials are selected based on successful performance in at least three successive trial evaluations. The regional trials allow the extensive testing of promising maize hybrids across a set of target environments. It offers the National Maize Programs of WCA the opportunity to select hybrids/OPVs with superior performance for further testing and release directly as a replacement for old and less-performing varieties or as source materials for their breeding programs. In addition to being dispatched to the NARS partners annually, IITA-MIP participates annually in the evaluation of these trials across multiple test environments. The *Striga* trials are also conducted annually at Mokwa and Abuja under *Striga*-infested (STRINF) and non-infested (STRNON) conditions.

Assessment of the breeding program through timely estimation of the genetic gains from selection is important to determine the progress that has been made and the effectiveness of the breeding approach used over the years. To demonstrate gains in grain yield and other agronomic attributes from selection, several studies comparing the performance of maize cultivars developed over time and evaluated in field trials conducted in common sets of environments were documented [8 –14]. These studies have been conducted by choosing genotypes representative of the breeding eras or cycles and evaluating them simultaneously in common sets of environments over the years. A few of the previous studies published by Menkir and Meseka [13] indicated a yield gain of 3.2% with a mean increase of 93.7 kg $ha^{-1}$ $yr^{-1}$ under Striga-infested environments in a set of 32 late/intermediate maturing maize hybrids developed over three breeding periods. Apraku *et al*. [15] evaluated 54 hybrids developed during three breeding periods of genetic enhancement. The authors reported annual genetic gains from selection in grain yield of 84.72 kg $ha^{-1}$ $yr^{-1}$ (4.05%) and 61 kg $ha^{-1}$ $yr^{-1}$ (1.56%), under multiple-stress and non-stress

environments, respectively. These studies necessitate a substantial resource investment. This includes time, difficulty in producing, packaging, and dispatching the seeds for the trials, logistical difficulties when reassembling historical genotypes. Additionally, genotypes developed years ago may perform differently in most recent years due to climate change resulting in new genotypes being favored. Furthermore, these studies do not facilitate monitoring of the effectiveness of breeding strategies to allow the exploration of the weaknesses to make the necessary adjustments. However, the effects of different trial management techniques and varying climatic conditions on genetic trends are usually eliminated to provide the most unbiased estimates of genetic gain [16].

In estimating genetic gains from selection over several breeding periods, numerous methodologies have been documented. Crespo-Herrera *et al*. [17] used the factor analytic model approach to estimate genetic gains in wheat. This approach regresses the differences in mean grain yield of the highest-yielding varieties on the mean yield of checks. The authors reported an annual genetic gain of 1.67% relative to the commercial check and 0.53% relative to the local check. Additionally, Menkir *et al*. [18]used the mixed model analysis to conduct genetic gain in late/intermediate maturity maize . The authors reported yield gain of 86.60 kg $ha^{-1}$ $yr^{-1}$ under *Striga* infestation and 102.44 kg $ha^{-1}$ $yr^{-1}$ under *Striga*-free condition. Breeding trials continuously consider the dynamics of adding new genotypes and eliminating the inferior ones. As a result, many authors [18 – 21] recommended the use of mixed linear model analysis to reliably estimate genetic gains for unbalanced data from multi-environment trials. Recently, The Consortium of International Agricultural Research Centers (CGIAR) has updated its recommendations, emphasizing the use of historical data sets to analyze genetic gain. The strategy is crucial to enable

regular monitoring of the progress of a breeding program and the efficacy of breeding methodologies employed for the development of superior genotypes.

The repeatability of the trial observed over the years may differ from that of other years and/or all years combined. The observed discrepancy could result from differences in climate conditions, trial management, or random chance due to the additive effect of the experimental errors. Timely assessment of the efficiency of the methodologies employed in a breeding program through the estimation of the repeatability and the estimates of the realized genetic gains over time in a breeding program is important. It allows the evaluation of the progress, the approach, and the effectiveness of breeding methodologies to be employed for planning new strategies or adopting the most appropriate method in the breeding program. Thus, the objectives of the present study were to; (i) determine the genetic improvement in grain yield and *Striga* adaptive traits and (ii) assess the repeatability of the trials in identification of the promising hybrids under both STRINF and STRNON conditions.

# Materials and Methods

## Development of the genetic materials

The early and extra-early maize breeding program of IITA started the inbred development in 1994. The program aimed at the development of *Striga* resistant inbred lines, hybrids, and OPVs. The early maturing populations TZE-W Pop DT STR C0, TZE-Y Pop DT STR C0, TZE Comp 5-Y C6, and TZE-W Pop × 1368 STR were initially used as the sources for *Striga* resistance and drought tolerance. These populations were valuable sources of genes for *Striga* resistance and drought tolerance and have been used to develop

several outstanding inbreds, hybrids, and OPVs in the early and extra-early breeding program. However, the levels of resistance to *Striga* in these populations were not as high as desired. In 2007, a program was initiated to increase the frequency of favorable alleles for *Striga* resistance and drought tolerance by using the $S_1$ family recurrent selection breeding scheme to introgressed genes from the wild maize relative *Zea diploperennis*. This resulted in the development of new generations of outstanding populations that combined improved levels of resistance to *Striga* and tolerance to drought and low soil nitrogen. The approaches that have been utilized in the development of the hybrids used in the present study have been described in detail by [22]. In summary, the germplasm sources were used to generate the $S_1$ lines which were advanced to the $S_4$ stages of inbreeding through repeated selfing and selection. The lines were evaluated under artificial *Striga* infestation and induced moisture stress after each inbreeding cycle. At the $S_4$ stage, 250–300 selected lines were crossed to broad-based testers to estimate the general combining abilities of the lines. Based on the performance in the test-crosses, 90–100 $S_4$ lines were advanced to the $S_6$ stage of inbreeding. The $S_6$ lines were used in development of the hybrids. The sets of hybrids developed, were first evaluated in progeny trials from where successful candidates were advanced to the second testing stage of the progeny trial and then moved to preliminary trials. Based on the genotypic performance, successful candidates were advanced to the regional trials and also evaluated and the data were used for this study.

A total of 159, early maturing white MST hybrids (MST1–159) developed by IITA-MIP, 5 farmer-preferred hybrids marketed conventional *Striga*-resistant hybrids/cultivars (LOC 1–5), and 13 hybrids (SCO 1–13) contributed by multi-national seed companies and commercialized in many African countries were used in the present study. The detailed

descriptions and the year of first testing of the hybrids in the regional trial are presented in Supplementary Table S1.

# Management of Field Trials

In the present study, we used historical data from trials conducted under STRINF and STRNON conditions at Abuja and Mokwa in Nigeria, from 2010 to 2021 growing seasons. The characteristics of the two test environments were presented in Supplementary Table S2. The alpha lattice design was employed for the trials containing more than twenty entries while RCBD was adopted for the trials that had not more than twenty entries. Each trial consisted of three replications with a plot comprising 2 rows, each 4 m long. The spacing between rows was 75 cm while plants within a row were 4 cm apart. Three seeds were placed into each hole and later thinned to two plants per hill resulting in a population density of 66,666 plants $ha^{-1}$. The hybrids were planted in two *Striga*-infested rows and two non-infested rows within each strip, planted exactly opposite each other and separated by a 1.5-meter alley. The artificial *Striga* infestation was achieved by putting about 5000 germinable *Striga* seeds per hill followed by planting maize seeds into the same holes as described in detail by Badu-Apraku and Fakorede [22]. To subject the maize plants to stress and encourage the production of strigolactones that promote good germination of *Striga* seeds and attachment of *Striga* plants to the roots of maize plants in *Striga*-infested plots, fertilizer application was delayed till about 30 days after planting [23]. The compound fertilizer NPK 15:15:15 was applied at the rate of 30 kg $ha^{-1}$ N, P, and K at 30 and 45 days after planting (DAP). Weeds other than *Striga* were removed mannually throughout the growing seasons.

## Traits measured

Data were collected on 10 *Striga*-adaptive traits including grain yield (YLD), days to 50% anthesis (DA), days to 50% silking (DS) anthesis-silking interval (ASI), ears per plant (EPP), ear aspect (EASP), emerged *Striga* plant at 8 WAP (ESP8), emerged *Striga* plant at 10 WAP (ESP10), *Striga* damage ratings at 8 WAP (SDR8) and *Striga* damage ratings at 10 WAP (SDR10). The detail descriptions of the traits studied were presented in Table 1.

**Table 1: Descriptions of the measured traits of early white maize hybrids evaluated in the regional trials under STRINF and STRNON conditionts at Abuja and Mokwa, 2010–2021.**

| Trait | Stage | Unit | Description |
|---|---|---|---|
| Grain yield (YLD) | Harvest | Kg/ha | Computed from the weight of shelled grain adjusted to 80% shelling percentage and corrected for 15% moisture content |
| Days to 50% anthesis (DA) | Flowering | Days | Days from planting to 50% pollen shed |
| Days to 50% silking (DS) | Flowering | Days | Days from planting to 50% silk emergence |
| Anthesis-silking interval (ASI) | Flowering | Days | The time interval between 50% anthesis and silking |
| Ears per plant (EPP) | Post-flowering | Numeric | Calculated by dividing the number of harvested ears by the number of plants harvested per plot |

| | | | |
|---|---|---|---|
| Ear aspect (EASP) | Harvest | Scale | Scored on a scale of 1 to 9, with 1 denoting ears that were clean, tidy, uniform, big, and full, and 9 denoting ears with undesirable characteristics |
| Emerged *Striga* plant at 8 and 10 WAP (ESP8 and ESP10) | Post-flowering | Count | The numbers of Striga plants that were counted at 8 and 10 WAP in the Striga-infested plots |
| *Striga* damage ratings at 8 and 10 WAP (SDR8 and SDR10) | Post-flowering | Scale | Scored on per plot basis on a scale of 1 to 9 where 1 = no damage, indicating normal plant growth and high resistance, and 9 = complete collapse or death of the maize plant |

## Statistical analyses

The historical data used in the analysis of the present study came from trials conducted under Striga-infested (STRINF) and *Striga* non-infested (STRNON) conditions. The analysis followed the following steps:

The first step, involved environment-level analyses with the environment is defined as the combination of trial name, location, year, and condition. We used the linear mixed model with the hybrids considered as random effects to estimate the broad-sense heritability a measure of experimental repeatability [24]. Trials with heritability values less than 0.1 were excluded from the estimated genetic gains. After excluding the environments with heritability estimates less than 0.1, another linear mixed model (equation 1) was fitted for the hybrids treated as a fixed effect to estimate the Best Linear

Unbiased Estimates (BLUEs), and weight (inverse of squared standard error) for each hybrid used for the second stage of the analyses as follows:

$$y_{ikj} = \alpha + h_i + \varphi_k + \rho_j + \varepsilon_{ikj} \quad (1)$$

where $y_{ikj}$ represents the yield value of the $i^{th}$ hybrid in the $k^{th}$ block and the $j^{th}$ replication. The overall mean is denoted by $\alpha$, the random effect of the $a^{th}$ hybrid is represented by $h_i$ , $\varphi_k$ is the effect of b$^{th}$ replication, $\rho_j$ is the effect of the kth block, and $\varepsilon_{ikj}$ is the random residual effect attributed to the yield value of the $i^{th}$ hybrid in the $k^{th}$ block and in the $j^{th}$ replication that follows the normal distribution $N(0,\ \sigma_e^2)$.

Secondly, we used the model represented by equation 2 [20] to analyse the BLUE values of grain yield and other traits obtained from equation 1 under STRINF and STRNON conditions as proposed by Mackay *et al*. [20]

$$Y_{ijklm} = \mu + \zeta_i + \eta_m + \lambda_{jlm} + \beta_{kjlm} + (\zeta\eta)_{im} + \xi_{lm} + \varepsilon_{ijklm} \quad (2)$$

Where $Y_{ijklm}$ is the BLUE value of yield of the $i^{th}$ hybrid in the $k^{th}$ block, in the $j^{th}$ replication, in the $l^{th}$ location and $m^{th}$ year. The overall mean is represented by $\mu$. The effect of the $i^{th}$ hybrid is denoted by $\zeta_i$ and the effect of the $m^{th}$ year is represented by $\eta_m$; $\lambda_{jlm}$ represents the effect of the $j^{th}$ replication in the $l^{th}$ location and the $m^{th}$ year. Also, $\beta_{kjlm}$ is the effect of the $k^{th}$ block in $j^{th}$ replication in the $l^{th}$ location and $m^{th}$ year. The interaction effect between the $(im)^{th}$ hybrid and year is represented by $(\zeta\eta)_{im}$, while $\xi_{lm}$ is the effect of location $l$ within year $m$; $\varepsilon_{ijklm}$ is the residual, attributable to the combined effects of within-trial error and hybrid x location within-year interaction. Heterogeneity in the residual variances was assumed. Thus, we fitted a distinct residual variance for each condition for every combination of location and year.

Lastly, we used the weighted regression to examine trends (realized genetic gain) in the data from the second stage of the analyses. The BLUEs of the hybrids and their year of origin in each condition were used as dependent and independent variables in the regression analyses respectively. We determined the percentage of each condition's genetic gain that could be attributed to genetic factors. The process involved calculating the ratio of the second stage's weighted regression slope plus the regression's y-intercept, and the product of the slope and year of the initial testing. The p-value < 0.05 for each trait from the summary of the weighted regression model was considered significant. The Best Linear Unbiased Predictions (BLUPs) yield of the hybrids in each environment from the fir st stage was visualized using the Boxplot. Similarly, the repeatability estimate of each trait over the years by location and condition was presented using a scatter plot. All analyses were performed in R [25] using the ASReml package [26]

# Results

## Genetic gains in grain yield and *Striga* adaptive traits

During the 12 years of development of the early maturing hybrids and genetic improvements, significant annual gains in grain yield of 3.40 and 3.71 % with changes of 76.87 and 127.02 kg $ha^{-1}$ $yr^{-1}$ were recorded under STRINF and STRNON conditions, respectively. The significant increases in grain yield were associated with significant increases of 1.84 and 1.79 % in ears per plant and significant decreases of 1.31 and 1.75 % in the ear aspect under STRINF and STRNON conditions, respectively. Under the STRINF condition, significant genetic gains of -3.04 and -2.25 % in *Striga* damage rating at 8 and 10 WAP, respectively, and -1.27 % in the number of emerged *Striga* plants at 10WAP were

obtained (Table 2). However, there were no significant gains in anthesis silking interval under STRINF environments and days to anthesis and silking under STRINF and STRNON conditions (Table 2). Additionally, the regression line considering the hybrid's first year of evaluation (independent variable) and traits (dependent variable) showed a positive linear relationship for grain yield and EPP and a negative linear relationship for ESP10, SDR10, and EASP. The breeding period under both conditions did not affect the number of days to flowering and ASI across STRINF and STRNON conditions (Fig 1a-1f).

**Table 2: Percent genetic gains in grain yield and other *Striga*-adaptive traits of maize hybrids evaluated under STRINF and STRNON conditions in Nigeria between 2010 – 2021.**

| Trait | Management | % Genetic Gain (yearr$^{-1}$) | slope | slope.sd | intercept |
|---|---|---|---|---|---|
| YLD | STRINF | 3.40** | 76.87 | 13.32 | -152175.53 |
| | STRNON | 3.71** | 127.02 | 21.65 | -252000.76 |
| DA | STRINF | 0.12ns | 0.17 | 0.03 | -283.37 |
| | STRNON | 0.11ns | 0.06 | 0.04 | -63.30 |
| DS | STRINF | 0.10ns | 0.05 | 0.03 | -52.78 |
| | STRNON | 0.11ns | 0.06 | 0.05 | -72.47 |
| ASI | STRINF | -0.60ns | -0.01 | 0.02 | 32.56 |
| | STRNON | -5.62** | -0.13 | 0.02 | 272.06 |
| EASP | STRINF | -1.31** | -0.07 | 0.01 | 145.94 |
| | STRNON | -1.75** | -0.08 | 0.02 | 167.23 |
| EPP | STRINF | 1.84** | 0.01 | 0.00 | -25.00 |
| | STRNON | 1.79** | 0.01 | 0.00 | -26.62 |

| | | | | | |
|---|---|---|---|---|---|
| ESP8 | STRINF | -0.97ns | -0.31 | 0.22 | 653.83 |
| ESP10 | STRINF | -1.27* | -0.45 | 0.20 | 948.38 |
| SDR8 | STRINF | -3.04** | -0.16 | 0.02 | 328.14 |
| SDR10 | STRINF | -2.25** | -0.13 | 0.02 | 274.04 |

Sd:standard deviation; ** : Significant; ns : Not Significant; YLD: grain yield; DA: days to 50% anthesis; DS: days to 50% silking; ASI: anthesis-silking interval; EPP: ears per plant; EASP: ear aspect; ESP8: emerged *Striga* plant at 8 WAP; ESP10: emerged *Striga* plant at 10 WAP; SDR8: *Striga* damage ratings at 8 WAP; SDR10: *Striga* damage ratings at 10 WAP

## Grain yield performance of early maturing maize hybrids evaluated in the regional trials across STRINF and STRNON conditions, 2010 –2021.

In the present study, three types of hybrids were evaluated, MST, SCO and LOC. The LOC cultivars comprised five hybrids, among them three common cultivars were evaluated over the period of 12 years. Additionally, the MST hybrids were evaluated for 2-6 years making the data well connected to allow an estimate of realized genetic gains. We assessed the yield gains under STRINF and STRNON conditions by comparing the MST hybrids' average grain yield performance to the SCO and LOC hybrids (Fig 2). The results showed that, under STRINF, the mean grain yield of MST hybrids ranged from 1091 kg $ha^{-1}$ for hybrid developed in 2012 to 4287 kg $ha^{-1}$ for hybrid developed in 2018. Similarly, the mean grain yield for SCO hybrids varied from 965 kg $ha^{-1}$ to 2964 kg $ha^{-1}$ and LOC hybrids varied from 1914 kg $ha^{-1}$ to 2644 kg $ha^{-1}$ (Supplementary Table S1). On another hand, mean grain yield of the hybrids evaluated under STRNON condition ranged from 2268 kg $ha^{-1}$ to 6861 kg $ha^{-1}$ for the MST hybrids and from 3590 kg $ha^{-1}$ to 6644 kg $ha^{-1}$ for SCO hybrids while 2392 kg $ha^{-1}$ to 4088 kg $ha^{-1}$ were recorded for LOC hybrids.

Generally, the MST hybrids had higher mean grain yields than the SCO and the LOC hybrids under STRINF and STRNON conditions, (Fig 2).

## Repeatability estimates of the STRINF and STRNON trials

The results of the repeatability estimates of the trials conducted in the STRINF and STRNON conditions from 2010 to 2021 are shown in Table 3 and Fig 3a–f. Grain yield had repeatability estimates ranging from 0.00 for a trial conducted under STRINF condition at Mokwa in 2016 to 0.91 for the STRINF trial evaluated at Abuja during the 2017 growing season. Among the trials evaluated in Abuja and Mokwa under STRINF and STRNON conditions, only three trials (about 8 %) had repeatability estimates of less than 30% for grain yield, about 92% of the trials had repeatability greater than 30 % for grain yield (Fig 3). Similarly, days to anthesis had repeatability estimates varying from 0.34 at Mokwa under STRINF condition in 2010 to 0.97 at Abuja in 2017 under STRINF condition. Days to silking had had repeatability estimates varied from 0.59 under STRINF condition at Mokwa in 2010 to 0.95 at Abuja in 2017 under STRINF condition. Anthesis silking interval had repeatability ranging from 0.00 at Mokwa under STRNON condition in 2020 to 0.81 at Abuja under STRINF condition in 2016 while repeatability estimates for ear aspect varied from 0.00 at Mokwa under STRNON condition in 2015 to 0.92 at Mokwa under STRINF condition in 2020 and ears per plant had repeatability varying from 0.00 at Mokwa under STRNON condition in 2015 to 0.84 at Abuja under STRINF condition in 2019 (Table 3, Fig 3). Furthermore, repeatability estimates under SRTINF environments varied from 0.10 in 2010 to 0.82 in 2020 at Abuja for ESP8 and from 0.00 in 2015 to 0.82 in 2020 at Abuja for ESP10 while 0.40 in 2010 at Abuja to 0.93 in 2020 at Mokwa for SDR8 and from 0.52 in 2016 to 0.95 at Mokwa in 2020 for SDR10 (Table 3, Fig 3).

**Table 3. Repeatability estimate of grain yield and other *Striga*-adaptive traits evaluated under STRINF and STRNON conditions at Mokwa and Abuja, 2010 –2021**

| Trait | Repeatability | | Year | Location | Management |
|---|---|---|---|---|---|
| YLD | minimum | 0.00 | 2016 | Mokwa | STRINF |
| | maximum | 0.91 | 2017 | Abuja | STRINF |
| DA | minimum | 0.34 | 2010 | Mokwa | STRINF |
| | maximum | 0.97 | 2017 | Abuja | STRINF |
| DS | minimum | 0.59 | 2010 | Mokwa | STRINF |
| | maximum | 0.95 | 2017 | Abuja | STRINF |
| ASI | minimum | 0.00 | 2020 | Mokwa | STRNON |
| | maximum | 0.81 | 2016 | Abuja | STRINF |
| EASP | minimum | 0.00 | 2015 | Mokwa | STRNON |
| | maximum | 0.92 | 2020 | Mokwa | STRINF |
| EPP | minimum | 0.00 | 2015 | Mokwa | STRNON |
| | maximum | 0.84 | 2019 | Abuja | STRINF |
| ESP8 | minimum | 0.10 | 2010 | Abuja | STRINF |
| | maximum | 0.82 | 2020 | Abuja | STRINF |
| ESP10 | minimum | 0.00 | 2015 | Abuja | STRINF |
| | maximum | 0.82 | 2020 | Abuja | STRINF |
| SDR8 | minimum | 0.40 | 2010 | Abuja | STRINF |
| | maximum | 0.93 | 2020 | Mokwa | STRINF |
| SDR10 | minimum | 0.52 | 2016 | Mokwa | STRINF |
| | maximum | 0.95 | 2020 | Mokwa | STRINF |

YLD: grain yield; DA: days to 50% anthesis; DS: days to 50% silking; ASI: anthesis-silking interval; EPP: ears per plant; EASP: ear aspect; ESP8: emerged *Striga* plant at 8 WAP; ESP10: emerged *Striga* plant at 10 WAP; SDR8: *Striga* damage ratings at 8 WAP; SDR10: *Striga* damage ratings at 10 WAP

# Discussion

In addressing the challenges of chronic food insecurity, climate change, environmental sustainability, and improving the economic well-being of farmers in SSA, we used 12 years of historical data to explore three aspects: i) Accelerated genetic gains in grain yield and *Striga* resistance indicator traits of early maturing tropical maize hybridsto explore the most effective use of the genetic variability of early maturing tropical maize populations of IITA. ii) Compare the *performance* of *Striga*-tolerant hybrids developed in the breeding program of IITA to marketed farmer-preferred cultivars and hybrids developed by multinational seed companies and released in many African countries in an effort to justify the program's resource allocation. iii) Assess the repeatability of the trials in the exploration of the  genotypic potential of the hybrids under STRINF and STRNON conditions in an effort to develop maize hybrids that could withstand the devastating effects of *Striga* infestation in *Striga*-prone environments.

## Realized genetic gains in grain yield and othe *Striga* adaptive traits

Towards the efforts to make appropriate adjustments in the IITA-MIP , there is need for the investigation of the genotypic potential of the program and introgression of genetic variability into the breeding populations, as well as accelerate the rate of genetic advancement of the breeding program. The results of the present studyrevealed that significant progress had been made during the past 12 years of genetic enhancements with

annual gains in grain yield reaching 3.40 % $yr^{-1}$ and 3.71 % $yr^{-1}$ under STRINF and STRNON conditions, respectively. The gains were associated with relative changes of 76.87 kg $ha^{-1}$ $yr^{-1}$ and 127.02 kg $ha^{-1}$ $yr^{-1}$ under STRINF and STRNON conditions, respectively. The progress attained in the present study was considerably higher compared to the annual genetic gains in grain yield of 2.91 % $yr^{-1}$ and 1.33 % $yr^{-1}$ archieved under low and high N environments, respectively, [9], 1.93 % $yr^{-1}$ under drought and well-watered conditions[14], and 0.74 % $yr^{-1}$ and 1.47 % $yr^{-1}$ under drought and full irrigation, respectively [18]. Additionally, Tarekegne *et al.* [27] assessed the genetic gains in grain yield and other traits of the early maturing maize hybrids developed during the period 2000–2018 by International Maize and Wheat Improvement Center (CIMMYT) Southern African breeding program and reported annual yield gains of 2.21%, 2.13%, 1.64% and 1.78%, under low N, managed drought, random stress and optimum conditions. However, Badu-Apraku et al., [1] reported genetic gains of 4.82 % $yr^{-1}$ and 1.24 % $yr^{-1}$ under STRINF and STRNON conditions, respectively in early maturing maize hybrids, while de la Vega *et al*. [19] reported genetic gains of 2.58 % $yr^{-1}$ and 2.30 % $yr^{-1}$ under STRINF and STRNON conditions, respectively, in late-maturing maize hybrids. This implied that under *Striga* infestation, the hybrids responded to selection more favorably than under drought or low N conditions. This could be attributed to the fact that under artificial *Striga* infestated condition the trials were conducted under more controlled environments compared to drought and low N stress conditions, allowing for better accuracy in the selection process. Additionally, the *Striga* resistance may be more oligogenic than polygenic which is more commonly observed under drought and low N tolerance. Since the artificial *Striga* infestation process utilized for the development of inbred lines and the evaluation of hybrids has remained unchanged for the past two

decades, it could have continuously resulted in high levels of precision, which might have major impact on the achievement of a higher rate of genetic gain in grain yield under STRINF. Alternatively, it could be because more traits were studied during the *Striga* infestation trials than during the drought and low N trials, which could have aided breeders in precisely selecting the most promising hybrids. Furthermore, the low yield improvements observed under drought or low N stresses may be related to the adoption of *Striga*-resistant populations enhanced through $S_1$ recurrent selection without the infusion of tropical donor lines possessing superior genes for drought or low N tolerance as parents of the MST hybrids of IITA-MIP[19]. The significant increases in grain yield were associated with significant increases of 1.84 % $yr^{-1}$ and 1.79 % $yr^{-1}$ in ears per plant and significant decreases of 1.31 % $yr^{-1}$ and 1.75 % $yr^{-1}$ in the ear aspect under STRINF and STRNON conditions, respectively. Additionally, the increases in grain yield under STRINF conditions were accompanied by a significant decrease of 3.04 % $yr^{-1}$, 2.25 % $yr^{-1}$, and 1.27 % $yr^{-1}$ in *Striga* damage ratings at 8 and 10 WAP and the number of emerged *Striga* plants at 10 WAP, respectively. Similar results were obtained by Badu-Apraku *et al*. [15] and Menkir *et al*. [18]. Interestingly, the 12 years breeding period did not affect the flowering period of the early-maturing maize hybrids.

# Comparative performance of the hybrids

The success of a breeding program depends on the establishment of the right selection criteria and the capacity to select hybrids superior in performance and/or competitive in grain yields under stressful conditions and across diverse environments. The comparative performance of MST hybrids to LOC and SCO hybrids across both STRINF and STRNON conditions revealed a clear distinction between the hybrids of the

three groups. The MST hybrids performed exceptionally well in both STRINF and STRNON conditions. The result confirmed that MST with improved levels of *Striga* resistance outperformed those of SCO and LOC hybrids in contrasting environments. Our findings suggested that the breeding strategies, including recurrent selection, backcrossing, hybridization, evaluation, and selection, employed to develop the MST hybrids during the 12-year breeding period were highly effective. This implied that MST hybrids possessed favorable genes that made them display better performers than the SCO and LOC hybrids. Therefore, the accumulation of new favorable alleles through rapid breeding cycles was one of the possible scenarios that significantly increases the rate of genetic gains.

## Assesment of repeatability

Evaluating the repeatability of the STRINF and STRNON trials for assessing and exploring the genotypic potential of the hybrids was the focus of the present study. Unreliable or inconsistent repeatability estimates of the trials could have hindered the progress of accelerated genetic gains, as researchers could have wasted precious time and resources building on flawed or irreproducible findings. Reliable methodologies and experimental designs might have contributed to the overall quality of the scientific experiments. Rigorous methods help ensure that the results are a true reflection of the phenomenon under investigation, rather than being influenced by confounding variables or errors. In a nutshell, repeatability was essential in the assessment of the trials because it ensured the accuracy, consistency, trustworthiness, and validity of the trials. When the random error was restricted, the variations observed in the trial resulted solely in significant genotypic differences, environment, and genotype × environment (G×E)

interactions. Nevertheless, the overall observed variation in the experiment was considerably influenced by genotype, environment, and G×E interactions, determining the repeatability of the trial, considering the combined effects of genotype, environment, G×E interactions, and random errors. Therefore, the disparities in the repeatability estimates of the trials conducted over a long period may be mostly due to the experimental procedures used/random error. We estimated the repeatability of the STRINF and STRNON trials conducted between 2010 and 2021 for each of the measured traits.

The highest repeatability was observed for ears per plant and flowering dates (Fig 3), indicating that these traits would not be influenced largely by the environment and the G×E interactions. This suggested that flowering dates and ears per plant could be used to evaluate the effectiveness of the STRINF and STRNON trials in the exploration of the genotypic potential of the hybrids. However, the lowest average repeatability was observed with the emerged *Striga* plant at 8 and 10 WAP. Thus, the emerged *Striga* plant at 8 and 10 WAP should not be used to determine the efficiency of the STRINF and STRNON trials in the exploration of the  genotypic potential of the hybrids. This is not surprising because the maize hybrids had different levels of stimulations by the strigolactones, which have led to uneven germination of S*triga* seeds and their attachments to the host [29] . Additionally, a combination of factors encountered during trial evaluation at the different locations including distinct soil properties, nutrient contents, and pH levels, could have affected the emergence of the *Striga* plant [30]. Generally, a good level of repeatability was observed in the early maturing maize trials conducted under STRINF and STRNON conditions between the 2010 and 2021 growing seasons, indicating the potential of the trials in the identification of promising hybrids for commercialization in the SSA for increased food security and poverty alleviation.

## Conclusions

The present study used 12 years of historical data to analyse the genetic gains in grain yield and associated changes in yield-related traits under STRINF and STRNON conditions and to estimate the repeatability of the of the studied traits. Annual genetic gains of 3.40% $yr^{-1}$ and 3.71 % $yr^{-1}$ were obtained under STRINF and STRNON conditions, respectively. The flowering dates and ears per plant had the highest repeatability estimates. These traits have the lowest multi-dimensionalities, independent of environment influence and as such suitable for assessing repeatability of the trials. The emerged *Striga* plant at 8 and 10 WAP were identified as unsuitable traits for determining the efficiency of the trials particularly in exploring the genotypic potential of the hybrids. Significant genetic gains had been made and a good level of repeatability was observed in the early maturing maize trials. This indicates that the quality of the STRINF and STRNON trials was adequate for identification of outstanding hybrids for commercialization in the SSA to mitigate food insecurity and enhance poverty alleviation.

## List of figures

**Figure1a–f. Regression line of grain yield and other Striga adaptive traits (dependent variable) on year of development (independent variable) of hybrids evaluated in regional trials under STRINF conditions at Abuja and Mokwa, 2010 – 2021.**

**Figure 2. Box plot of the average grain yield of the MST, SCO, and LOC early maturing maize hybrids evaluated in the regional trials under STRINF and STRNON conditions at Abuja and Mokwa, 2010 – 2021.**

**Figure 3a–f. Scattered plot of the repeatability estimates for grain yield and other Striga adaptive traits evaluated under STRINF and STRNON conditions at Abuja and Mokwa 2010 –2021**

# Acknowledgments

The authors are grateful to the IITA-MIP, EBS, and Biometric Unit technical, field, and administrative staff for providing technical, field, and administrative assistance.

# Data availability statement

The raw data analyzed in this manuscript were available in IITA CKAN repository online at: https://doi.org/10.25502/8a4f-h291/d

# Supporting Information

Table S1: Mean grain yield and other Striga adaptive traits, year of first testing in the regional trial of the multiple stress tolerant hybrids under STRINF condition in Nigeria, 2010 –2021

Table S2: Description of test locations used for evaluation of early maize hybrids under STRINF condition in Nigeria, 2010 –2021

# Conflict of interest

The authors declare that there is no conflict of interest.

# References


[1] B. Badu-Apraku, G. B. Adu, A. M. Yacoubou, J. Toyinbo, and S. Adewale, "Gains in genetic enhancement of early maturing maize hybrids developed during three breeding periods under striga-infested and striga-free environments," *Agronomy*, vol. 10, no. 8, 2020, doi: 10.3390/agronomy10081188.

[2] I. M. Mwangangi, L. Büchi, S. M. Haefele, L. Bastiaans, S. Runo, and J. Rodenburg, "Combining host plant defence with targeted nutrition: key to durable control of hemiparasitic Striga in cereals in sub-Saharan Africa?," 2021. doi: 10.1111/nph.17271.

[3] I. C. Akaogu *et al.*, "Inheritance of Striga hermonthica adaptive traits in an early-maturing white maize inbred line containing resistance genes from Zea diploperennis," *Plant Breeding*, vol. 138, no. 5, 2019, doi: 10.1111/pbr.12707.

[4] G. Ejeta, "The striga scourge in africa: A growing pandemic. In: Ejeta G, Gressel J, editors. Integrating New Technologies for Striga Control: Towards Ending the Witch-Hunt," *World Scientific Publishing Company*, 2007.

[5] S. K. Kim, V. O. Adetimirin, C. Thé, and R. Dossou, "Yield losses in maize due to Striga hermonthica in West and Central Africa," *Int J Pest Manag*, vol. 48, no. 3, 2002, doi: 10.1080/09670870110117408.

[6] B. A. Kountche *et al.*, "Suicidal germination as a control strategy for Striga hermonthica (Benth.) in smallholder farms of sub-Saharan Africa," *Plants People Planet*, vol. 1, no. 2, 2019, doi: 10.1002/ppp3.32.

[7] S. K. Kim, IITA, ICRISAT, and IDRC, "Breeding maize for striga tolerance and the development of a field infestation technique," *Combating Striga in Africa*, 1988.

[8] R. O. Abdulmalik *et al.*, "Genetic gains in grain yield of a maize population improved through marker assisted recurrent selection under stress and non-stress conditions in West Africa," *Front Plant Sci*, vol. 8, 2017, doi: 10.3389/fpls.2017.00841.

[9] B. Badu-Apraku, M. A. B. Fakorede, and A. M. Abubakar, "Accelerated Genetic Gains in Early-Maturing Maize Hybrids following Three Periods of Genetic Enhancement for Grain Yield

under Low and High Soil-Nitrogen Environments," *Plants*, vol. 11, no. 9, May 2022, doi: 10.3390/plants11091208.

[10] D. N. Duvick, "Genetic progress in yield of United States maize (Zea mays L.)," 2005.

[11] L. Echarte, L. Nagore, J. Di, M. Cambareri, M. Robles, and A. Della, "Grain Yield Determination and Resource Use Efficiency in Maize Hybrids Released in Different Decades," in *Agricultural Chemistry*, 2013. doi: 10.5772/55287.

[12] B. Masuka *et al.*, "Gains in maize genetic improvement in eastern and southern Africa: I. CIMMYT hybrid breeding pipeline," *Crop Sci*, vol. 57, no. 1, 2017, doi: 10.2135/cropsci2016.05.0343.

[13] A. Menkir and S. Meseka, "Genetic improvement in resistance to striga in tropical maize hybrids," *Crop Sci*, vol. 59, no. 6, 2019, doi: 10.2135/cropsci2018.12.0749.

[14] M. Oyekunle *et al.*, "Gains in grain yield of released maize (Zea mays L.) cultivars under drought and well-watered conditions," *Exp Agric*, vol. 55, no. 6, pp. 934–944, Dec. 2019, doi: 10.1017/S0014479719000048.

[15] B. Badu-Apraku, A. M. Abubakar, G. B. Adu, A.-M. Yacoubou, S. Adewale, and I. I. Adejumobi, "Enhancing Genetic Gains in Grain Yield and Efficiency of Testing Sites of Early-Maturing Maize Hybrids under Contrasting Environments," *Genes (Basel)*, vol. 14, no. 10, p. 1900, Sep. 2023, doi: 10.3390/genes14101900.

[16] B. M. Prasanna *et al.*, "Genetic trends in CIMMYT's tropical maize breeding pipelines," *Sci Rep*, vol. 12, no. 1, Dec. 2022, doi: 10.1038/s41598-022-24536-4.

[17] L. A. Crespo-Herrera *et al.*, "Genetic yield gains in CIMMYT'S international elite spring wheat yield trials by modeling the genotype × environment interaction," *Crop Sci*, vol. 57, no. 2, pp. 789–801, Mar. 2017, doi: 10.2135/cropsci2016.06.0553.

[18] A. Menkir *et al.*, "Estimating genetic gains for tolerance to stress combinations in tropical maize hybrids," *Front Genet*, vol. 13, Dec. 2022, doi: 10.3389/fgene.2022.1023318.

[19] A. J. de la Vega, I. H. DeLacy, and S. C. Chapman, "Progress over 20 years of sunflower breeding in central Argentina," *Field Crops Res*, vol. 100, no. 1, 2007, doi: 10.1016/j.fcr.2006.05.012.

[20] I. Mackay, A. Horwell, J. Garner, J. White, J. McKee, and H. Philpott, “Reanalyses of the historical series of UK variety trials to quantify the contributions of genetic and environmental factors to trends and variability in yield over time,” *Theoretical and Applied Genetics*, vol. 122, no. 1, 2011, doi: 10.1007/s00122-010-1438-y.

[21] H. P. Piepho and J. Möhring, “Selection in cultivar trials—Is it ignorable?,” *Crop Sci*, vol. 46, no. 1, 2006, doi: 10.2135/cropsci2005.04-0038.

[22] B. Badu-Apraku and M. A. B. Fakorede, *Advances in genetic enhancement of early and extra-early maize for sub-Saharan Africa*. 2017. doi: 10.1007/978-3-319-64852-1.

[23] S. K. Kim, A. Y. Akintunde, and P. Walker, “Responses of maize inbreds during development of Striga hermonthica infestation,” *Maydica*, vol. 44, no. 4, 1999.

[24] B. R. Cullis, A. B. Smith, and N. E. Coombes, “On the design of early generation variety trials with correlated data,” *J Agric Biol Environ Stat*, vol. 11, no. 4, 2006, doi: 10.1198/108571106X154443.

[25] R Core Team., “R: A language and environment for statistical computing. R Foundation for Statistical Computing, Vienna, Austria.,” *Open J Stat*, vol. 13, no. 02, 2023.

[26] a R. Gilmour, B. J. Gogel, B. R. Cullis, and R. Thompson, “ASReml user guide release 3.0,” *VSN International Ltd*, 2009.

[27] A. Tarekegne *et al.*, “Genetic gains in early maturing maize hybrids developed by the International Maize and Wheat Improvement Center in Southern Africa during 2000–2018,” *Front Plant Sci*, vol. 14, 2023, doi: 10.3389/fpls.2023.1321308.

[28] B. Badu-Apraku, G. B. Adu, A. M. Yacoubou, J. Toyinbo, and S. Adewale, “Gains in genetic enhancement of early maturing maize hybrids developed during three breeding periods under striga-infested and striga-free environments,” *Agronomy*, vol. 10, no. 8, 2020, doi: 10.3390/agronomy10081188.

[39] M. Jamil, F. K. Kanampiu, H. Karaya, T. Charnikhova, and H. J. Bouwmeester, “Striga hermonthica parasitism in maize in response to N and P fertilisers,” *Field Crops Res*, vol. 134, 2012, doi: 10.1016/j.fcr.2012.03.015.

[30] S.-K. Kim, “Horizontal resistance: core to a research breakthrough to combat Striga in Africa,” *Integrated Pest Management Reviews*, vol. 1, no. 4, 1996, doi: 10.1007/bf00139766.

Figure Click here to access/download;Figure;Figures.pptx

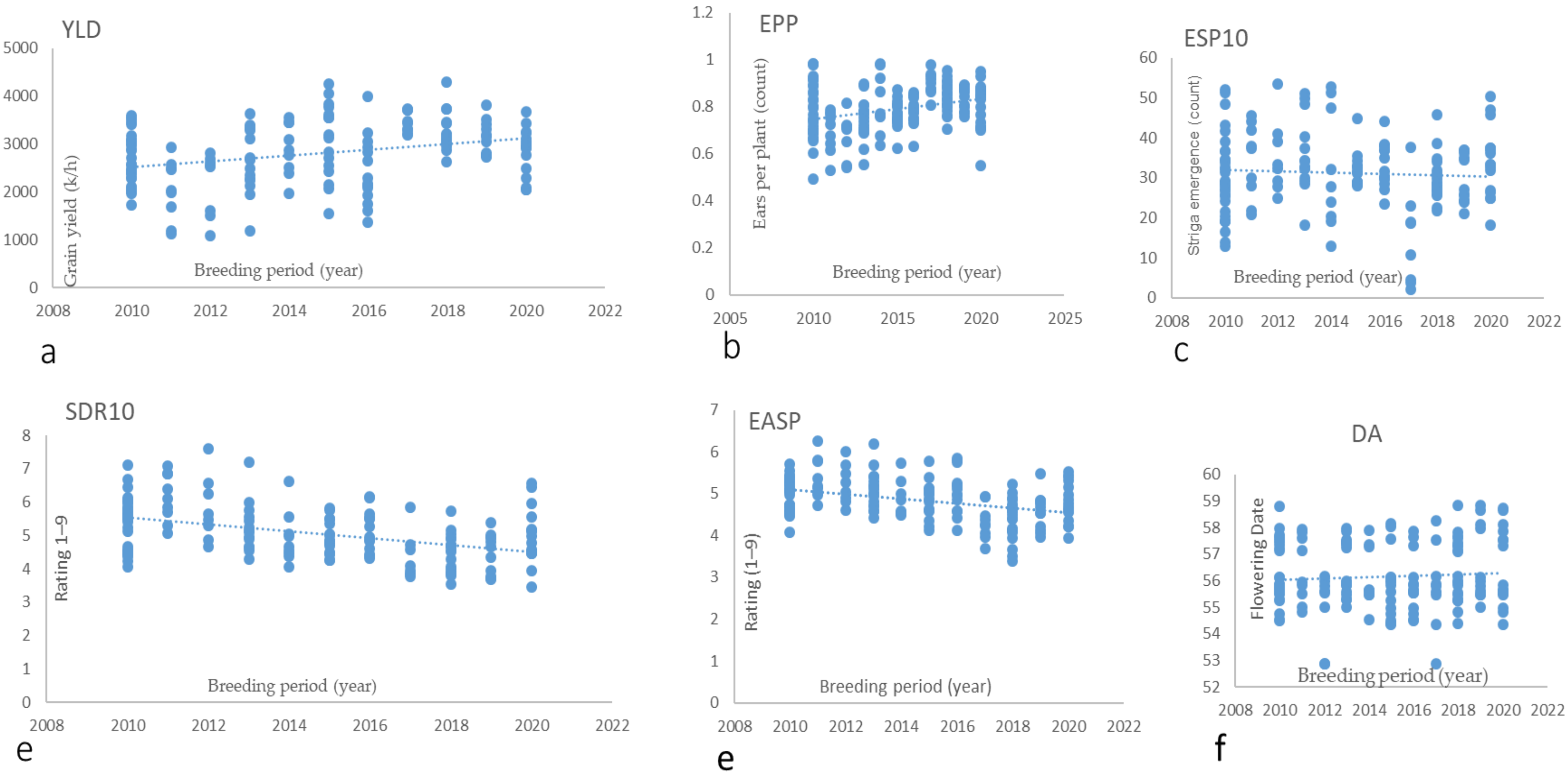


Figure1a–f: Regression line of grain yield and other *Striga* adaptive traits (dependent variable) on year of development (independent variable) of hybrids evaluated in regional trials under STRINF conditions at Abuja and Mokwa, 2010 – 2021.

YLD: grain yield; DA: days to 50% anthesis; EPP: ears per plant; EASP: ear aspect; ESP10: emerged *Striga* plant at 10 WAP; SDR10: *Striga* damage ratings at 10 WAP

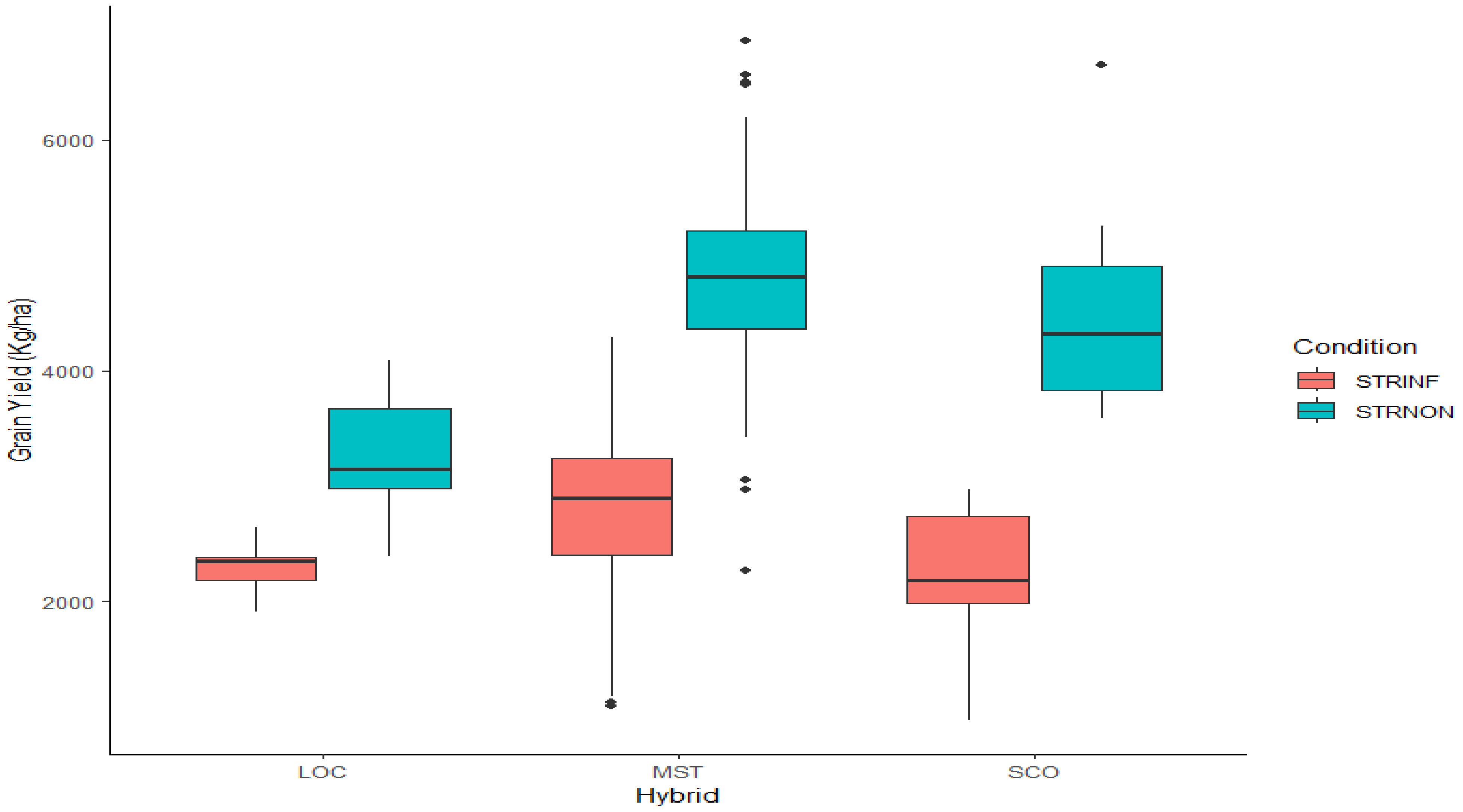


Figure 2: Box plot of the average grain yield of the MST, SCO, and LOC early maturing maize hybrids evaluated in the regional trials under STRINF and STRNON conditions at Abuja and Mokwa, 2010 – 2021.

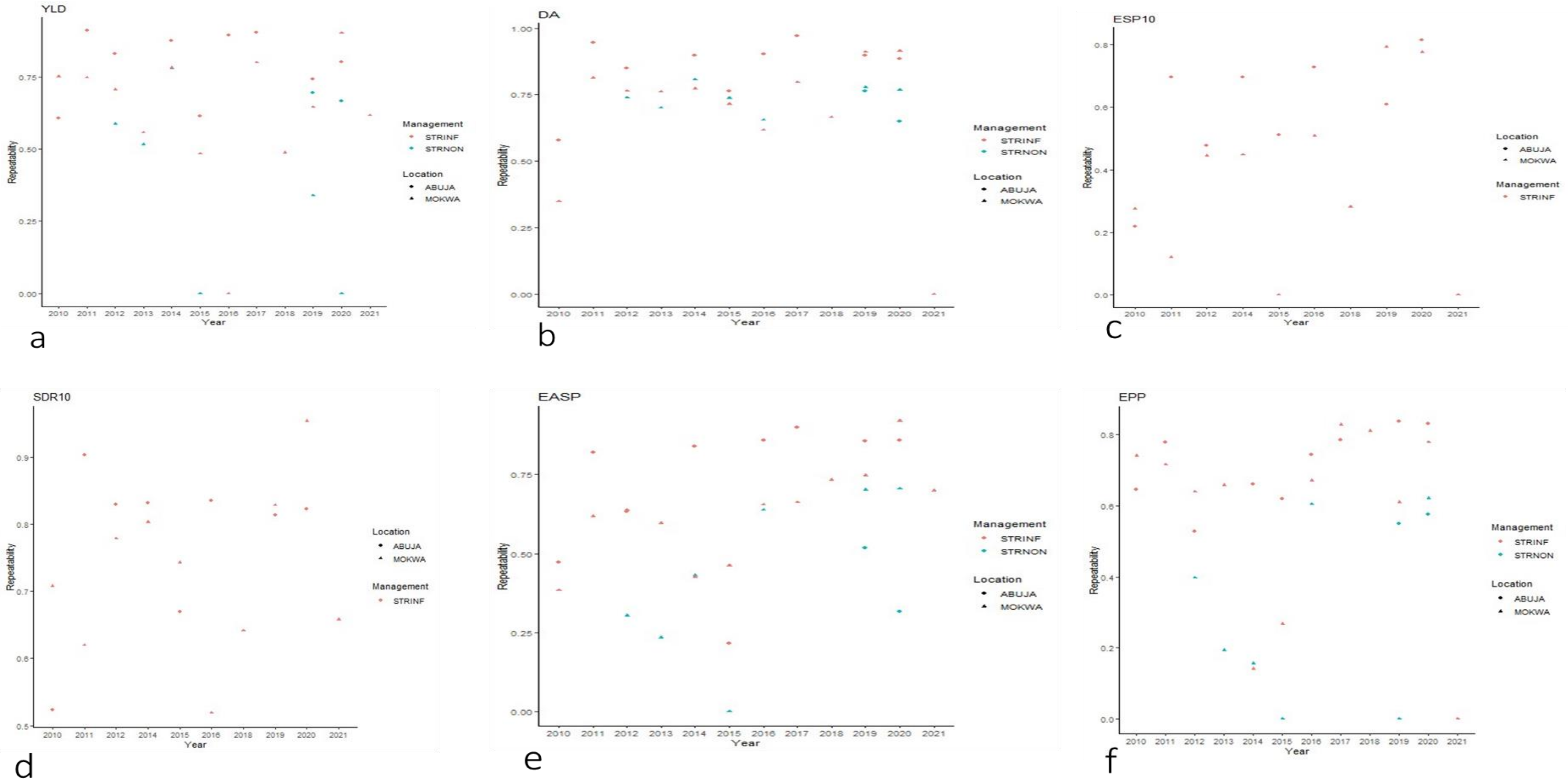


Figure 3a–f: Scattered plot of the repeatability estimates for grain yield and other Striga adaptive traits evaluated under STRINF and STRNON conditions at Abuja and Mokwa 2010 –2021.

YLD: grain yield; DA: days to 50% anthesis; EPP: ears per plant; EASP: ear aspect; ESP10: emerged *Striga* plant at 10 WAP; SDR10: *Striga* damage ratings at 10 WAP

Click here to access/download
**Supporting Information**
Supplemetary imformation.docx

# Repeatability and Genetic Advances in Early Maturing Maize Hybrid Trials Conducted under *Striga*-Infested and Non-Infested Conditions

Adamu Masari Abubakar[1,&2], Idris Ishola Adejumobi[1*], Kayode Rapheal Fowobaje[1], Ibnou Dieng[1], Saminu Zakariya[2], Simon Imoro[1] and Baffour Badu-Apraku[1],

[1]International Institute of Tropical Agriculture (IITA), PMB 5320, Oyo Road, Ibadan, Oyo State, Nigeria; a.abubakar@cgiar.org* (A.A.M.); i.adejumobi@cgiar.org (I.I.A.); k.fowobaje@cgiar.org (K.R.F.); i.dieng@cgiar.org (I.D.); s.imoro@cgiar.org (S.I); b.badu-apraku@cgiar.org (B.B.A.)

[2]Department of Plant Science, Institute for Agricultural Research, Ahmadu Bello University, P.M.B. 104, Zaria, Kaduna State, Nigeria. zacqkz@gmail.com (S.Z.)

* Corresponding author

E-mail: i.adejumobi@cgiar.org (I.I.A.)

## Abstract:

In sub-Saharan Africa (SSA), maize (*Zea mays* L.) is both a cash crop and an significant important staple crop. However, *Striga hermonthica* infection constrains its production and productivity. A total of 159 hybrids in from 21 international trials were

**Formatted:** Font: 18 pt, Not Bold
**Formatted:** Line spacing: Double
**Formatted:** Font: 14 pt, Not Bold
**Formatted:** Centered, Line spacing: Double
**Formatted:** Font: Not Bold
**Formatted:** Font: 14 pt, Not Bold
**Formatted:** Font: 14 pt, Not Bold
**Formatted:** Font: 14 pt
**Formatted:** Font: 14 pt
**Formatted:** Line spacing: Double
**Formatted:** Indent: Left: 0.5", Line spacing: Double
**Formatted:** Font: 18 pt
**Formatted:** Line spacing: Double
**Formatted:** Indent: Left: 0.5", Line spacing: Double
**Formatted:** Line spacing: Double

evaluated under *Striga*-infested (STRINF) and *Striga* non-infested (STRNON) conditions at Mokwa and Abuja, Nigeria, from 2010 to 2021. The data were used to (i) determine the genetic enhancements in grain yield and *Striga* adaptive traits and (ii) assess the repeatability of the trials in the exploration of the hybrid's genotypic potential. Significant annual genetic gains in grain yield of 3.40% and 3.71% with ~~changes~~ increases of 76.87 and 127.02 kg ha$^{-1}$ yr$^{-1}$ were recorded under STRINF and STRNON conditions, respectively. The genetic gains in grain yield were associated with 3.04 and 2.25 % decreases in *Striga* damage ratings at 8 and 10 weeks after planting (WAP), respectively, and 1.27 % in the number of emerged *Striga* plants at 10 WAP. The results indicated that ears per plant and flowering dates had~~ve~~ the highest repeatability estimates while the number of emerged *Striga* plants at 8 and 10 WAP ~~have~~ recorded the lowest repeatability. Generally, substantial progress has been ~~attained~~ achieved ~~and there is~~with a good level of repeatability estimates ~~in~~ for the early maturing maize hybrid trials evaluated under STRINF and STRNON conditions~~, 2010 to 2021. Therefore, the~~Those results have demonstrated that the breeding strategies adopted during the 12-year breeding period ~~were~~ have been effective, ~~indicating~~ and that the potential of the trials in the exploration of the ~~hybrids'~~ genetic potential of the hybrids for commercialization in the SSA for food security and poverty alleviation.

**Keywords**: selection, genetic gains, *Striga* resistance, grain yield, cereal crops.

# ~~1.~~ Introduction

Maize plays ~~a particularly special~~an important role as a staple food in the diets of millions of people in Africa, especially in ensuring food security. Maize production and

**Commented [A1]:** Please refer to table

**Commented [AAM(2R1]:** This is abstract section

**Formatted:** Font: 18 pt

**Formatted:** Indent: Left: 0.5", Line spacing: Double, No bullets or numbering

**Formatted:** Line spacing: Double

productivity are challenged faced by many a myriad constraints of which *Striga hermonthica* (purple witchweed), an obligate hemi-parasitic plant is one of the major factors causing substantial loss to grain yield on farmers' fields in sub-Saharan Africa (SSA) [1] . Even though *S. hermonthica* parasitizes the root systems of several cereals, maize is however the most preferred among the staple cereal crops. Every cropping season, soil-borne *Striga* seeds germinate and attach to maize plant root systems, causing plant toxicity and, yield reduction by siphoning water and nutrients from the host maize plant, and causingresulting in the death of the host plant in extreme cases. The outcome of this association is a significant reduction in the obtainable grain yield. *Striga* parasitism is more common in the Savanna of SSA region and resultings in food insecurity and rural poverty. *Striga*'s The lifespan of *Striga* depends on its ability to siphon nutrients and water from host plants to promote its growth and development. The *Striga* species have 5-7 days to establish themselves on a suitable host, otherwise, if the stored resource in the seeds is are exhausted, the radicles will wither [2] (Mwangangi et al., 2021. Following a successful attachment, *Striga* damages the host plant by penetrating and colonizing its root cells, lowering the host's productivity and function even before it emerges from the ground. *Striga* is a major threat to food security in SSA, where they negatively affect the growth and development of staple cereals crops. According to it causes yield losses of $7 to $10 billion worth and, could result in up to 100% losses in maize production , especially when the infestation is severe during the vegetative growth stage. [3], [4], [5], [6]

Breeding for *Striga* resistance has proven to be the most cost-effective, feasible, and long-term strategy for control by farmers with limited resources and who have an interest in protecting the environment. One of the main objectives of maize breeders in SSA is to develop *Striga* resistant/tolerant and commercially desirable maize hybrids. The scientists

of the International Institute of Tropical Maize Improvement Program (IITA-MIP) initiated *Striga* resistance research on maize in 1982 with most of the germplasm originating from the U.S. Corn Belt. Resistant inbreds and hybrids of IITA were first identified in 1983 from field testing at Mokwa in Nigeria [7]. Since then, breeders of the maize improvement program (MIP) have been working steadily to develop maize varieties/hybrids with elevated levels of *Striga* tolerance to support smallholder farmers in the targeted production environments. In this regard, IITA-MIP in collaboration with the National Agricultural Research Scientist (NARS) through deliberate teamwork, has developed and released several high-yielding multiple stress-tolerant (MST) (i.e., resistant to *Striga* and tolerant to drought and low N) hybrids and open-pollinated varieties (OPVs), many of which are suitable for several agro-ecologies in SSA. For the past two decades, the IITA-MIP has been developing and deploying *Striga*-tolerant hybrids ~~in~~ through regional trials to NARS in West and Central Africa (WCA) for evaluation and commercialization in the sub-region. The hybrids in the regional trials are selected based on successful performance in at least three successive trial evaluations. ~~The regional trials are an important vehicle for the exchange of germplasm among the IITA MIP and the National Maize Programs of WCA.~~ The regional trials allow the extensive testing of promising maize hybrids across a set of target environments. It offers the National Maize Programs of WCA the opportunity to select hybrids/OPVs with superior performance for further testing and release directly as a replacement for old and less-performing varieties or as source materials for their breeding programs. In addition to being dispatched to the NARS partners annually, IITA-MIP ~~every year~~ participates annually in the evaluation of these trials across multiple test environments ~~(stress and non stress)~~. The *Striga* trials are ~~being~~ also conducted annually

at Mokwa and Abuja under *Striga*-infested (STRINF) and non-infested (STRNON) conditions.

Assessment of the breeding program through timely ~~estimating~~ estimation of the genetic gains from selection is important ~~to~~ to~~for~~ ~~determining~~determine~~e~~ the progress that has been made and the effectiveness of the breeding approach used over the years ~~in achieving the required improvements to meet the present and future demands for maize~~. To demonstrate gains in grain yield and other agronomic attributes from selection, several studies comparing the performance of maize cultivars developed over time and evaluated in field trials conducted in common sets of environments ~~have been~~were documented [8], [9], [10], [11], [12], [13], [14], [15]. These studies have been conducted by choosing genotypes ~~that are~~ representative of the breeding eras or cycles and evaluating them simultaneously in common sets of environments over the years. ~~To report~~~~Example of the results~~ A few of the previous studies ~~u findings~~published by [14] ~~reported~~ indicated a yield gain of 3.2% with a mean increase of 93.7 kg $ha^{-1}$ $yr^{-1}$ under Striga-infested environments in a set of 32 late/intermediate maturing maize hybrids developed over three breeding periods. [16] evaluated 54 hybrids ~~(18 each in a breeding period)~~ developed during three breeding periods of genetic enhancement. The authors ~~and~~ reported annual genetic gains from selection in grain yield of 84.72 kg $ha^{-1}$ $yr^{-1}$ (4.05%) and 61 kg $ha^{-1}$ $yr^{-1}$ (1.56%), under multiple-stress and non-stress environments, respectively. These studies necessitate a substantial resource investment. This includes ~~particularly~~ time, difficulty in producing, packaging, and dispatching the seeds for the trials, logistical difficulties when reassembling historical genotypes. Additionally, ~~and~~ genotypes developed years ago may perform differently in most recent years due to ~~the~~ climate change ~~, where~~resulting in new genotypes ~~will~~ being favored. Furthermore, ~~In addition,~~ these studies do not facilitate

monitoring of the effectiveness of breeding strategies to allow the exploration of the weaknesses to make the necessary adjustments. However, the effects of different trial management techniques and varying climatic conditions on genetic trends are usually eliminated to provide the most unbiased estimates of genetic gain [17].

In estimating genetic gains from selection over several breeding periods, numerous methodologies have been documented. Crespo-Herrera et al., 2017) [18]used the factor analytic model approach to estimate genetic gains in wheat. This approach by regressesing the differences in mean grain yield of the highest-yielding varieties on the mean yield of cultivars used as checks. The authors from 8 years of historical data of wheat and reported an annual genetic gain of 1.67% relative to the commercial check and 0.53% relative to the local check. Additionally, [19]used the mixed model analysis to conduct genetic gain in late/intermediate maturity maize in. The authors of regional collaborative late maturing maize hybrid trials data conducted for 8 years and reported yield gain of 11.89 kg $ha^{-1}$ $yr^{-1}$ under managed drought stress, 86.60 kg $ha^{-1}$ $yr^{-1}$ under *Striga* infestation and , 62.65 kg $ha^{-1}$ $yr^{-1}$ under full irrigation, 102.44 kg $ha^{-1}$ $yr^{-1}$ under *Striga*-free conditions., and 53.11 kg $ha^{-1}$ $yr^{-1}$ across rainfed environments. Breeding trials continuously take into accountconsider the dynamics of adding new genotypes and eliminating the subpar inferior ones. As a result, many authors [19], [20], [21], [22]recommended the use of mixed linear model analysis to reliably estimate genetic gains for unbalanced data from multi-environment trials. Recently, One The Consortium of International Agricultural Research Centers (CGIAR) has updated its recommendations, emphasizing the use of historical data sets to analyze genetic gain. The strategy is crucial to enable regular monitoring of breeding programs'the progress of a breeding program and the efficacy of breeding methodologies employed for the development of superior genotypes.

The repeatability of the trial observed over the years may differ from that of other years and/or all years combined. The observed discrepancy could result from differences in climate conditions, trial management, or random chance due to the additive effect of the experimental errors. Sporadic Timely assessment of the efficiency of the methodologies employed in a breeding program through evaluation the estimation of the repeatability and the estimates of the realized genetic gains over time in a breeding program is importantdesirableimportant. It allows the evaluation of the progress, the approach, and the effectiveness of breeding methodologies to be employed for planning new strategies or adopting the most appropriate method being used to be adopted in the breeding program.

Early maturing *Striga* tolerant/resistant hybrids were subsequently evaluated under artificial STRINF and STRNON conditions across several locations and seasons for the past two decades. Therefore, the data is suitable for assessing the genetic improvement and evaluation of the repeatability of the trials. Thus, the objectives of the present study were to; (i) determine the genetic improvement in grain yield and *Striga* adaptive traits associated with the changes and (ii) assess the repeatability of the trials in the exploration of the hybrid's genotypic potentialidentification of the promising hybrids in under both STRINF and STRNON conditions. Information presented in this study would be crucial to guide breeders to assess the effectiveness of the strategies of the IITA MIP breeding programs and correct if necessary and justify the resources investment invested in the a breeding improvement program.

Formatted: Font: 18 pt

## 2. Materials and Methods

Formatted: Indent: Left: 0.5", Line spacing: Double, No bullets or numbering

Formatted: Font: 16 pt

Formatted: Line spacing: Double

### 2.1. Development of the genetic materials

Formatted: Indent: Left: 0.75", Line spacing: Double, No bullets or numbering

Formatted: Line spacing: Double

The early and extra-early maize breeding program of IITA started the inbreds development in 1994. The program aimed at the development of *Striga* resistant inbred lines, hybrids, and OPVs. The eEarly maturing populations TZE-W Pop DT STR C0, TZE-Y Pop DT STR C0, TZE Comp 5-Y C6, and TZE-W Pop × 1368 STR were initially used as the sources for *Striga* resistance and drought tolerance. These populations were valuable sources of genes for *Striga* resistance and drought tolerance and they have been used to develop several outstanding inbreds, hybrids, and OPVs in the early and extra-early breeding program. However, the levels of resistance to *Striga* in these populations were not as high as desired. In 2007, a program was initiated to increase the frequency of favorable alleles for *Striga* resistance and drought tolerance by usingprogram was started aimed at increasing the frequency of favorable alleles for *Striga* resistance and drought tolerance using the $S_1$ family recurrent selection breeding scheme to introgressed genes from the wild maize relative *Zea diploperennis*. This resulted in the development of new generations of outstanding populations that combined improved levels of resistance to *Striga* and tolerance to drought and low soil nitrogen. The approaches that have been utilized in the development of the hybrids used in the present study were have been described in detail by[23]. In summary, the disserved germplasm sources were used to generate the $S_1$ lines and which were advanced to the $S_4$ stages of inbreeding through repeated selfing and selection. The lines were evaluated under artificial *Striga* infestation and induced

Commented [A3]: A program was initiated to increase the frequency of favorable alleles for Striga resistance and drought tolerance by using...

Commented [AAM(4R3]: Corrected, thanks

moisture stress after each inbreeding cycle. At the $S_4$ stage, 250–300 selected lines were crossed to broad-based testers to estimate the general combining abilities of the lines. Based on the performance in the test-crosses, 90–100 $S_4$ lines were advanced to the $S_6$ stage of inbreeding. The $S_6$ lines were used in ~~the~~ development of the hybrids. ~~Every~~ The sets of hybrids developed, ~~they~~ were first evaluated in progeny trials from where successful candidates were advanced to the second testing stage of the progeny trial and then moved to preliminary trials. Based on the genotypic~~es'~~ performance~~s~~, successful candidates were advanced to the regional trials and also evaluated ~~whose~~ and the data were used for this study.

A total of 159, early maturing white MST hybrids (MST1–159) developed by IITA-MIP, 5 farmer-preferred hybrids marketed conventional *Striga*-resistant hybrids/cultivars (LOC 1–5), and 13 hybrids (SCO 1–13) ~~supplied~~ contributed by multi-national seed companies and commercialized in many African countries were used in the present study. The detailed descriptions and the year of first testing of the hybrids in the regional trial ~~of the hybrids~~ are presented in Supplementary Table S1.

## ~~2.2.~~ Management of Field Trials

Formatted: Font: 16 pt

Formatted: Indent: Left: 0.75", Line spacing: Double, No bullets or numbering

In the present study, we used historical data from trials conducted under STRINF and STRNON conditions at Abuja and Mokwa in Nigeria, from 2010 to 2021 growing seasons. The characteristics of the two test environments were presented in Supplementary Table S2. The alpha lattice design was employed for the trials containing more than twenty entries while RCBD was adopted for the trials that had not more than twenty entries. Each trial ~~had~~ consisted of three replications with a plot ~~consisting of~~comprising 2 rows, each 4 m long. The spacing

Formatted: Line spacing: Double

between rows was 75 cm while plants within a row were 4 cm apart. Three seeds were placed into each hole and later thinned to two plants per hill resulting in a population density of 66,666 plants $ha^{-1}$. The ~~same~~ hybrids w~~as~~ere planted in two *Striga-*infested rows and two non-infested rows within each strip, planted exactly opposite each other and separated by a 1.5-meter alley. The artificial *Striga* infestation was achieved by putting about 5000 germinable *Striga* seeds per hill followed by planting maize seeds into the same holes as described in detail by [23]. To subject the maize plants to stress and encourage the production of strigolactones that promote good germination of *Striga* seeds and attachment of *Striga* plants to the roots of maize plants in *Striga*-infested plots, fertilizer application was delayed till about 30 days after planting[24]. The compound fertilizer NPK 15:15:15 was ~~used to apply~~applied at the rate of 30 kg $ha^{-1}$ N, P, and K at 30 and 45 days after planting (DAP). Weeds other than *Striga* were removed ~~by hand~~mannually throughout the growing seasons.

## ~~2.3.~~ Traits Measured

Data were collected on 10 *Striga*-adaptive traits including grain yield (YLD), days to 50% anthesis (DA), days to 50% silking (DS) anthesis-silking interval (ASI), ears per plant (EPP), ear aspect (EASP), emerged *Striga* plant at 8 WAP (ESP8), emerged *Striga* plant at 10 WAP (ESP10), *Striga* damage ratings at 8 WAP (SDR8) and *Striga* damage ratings at 10 WAP (SDR10). The details descriptions of the traits studied were presented in Table 1.

**Table 1: Descriptions of the measured traits of early white maize hybrids evaluated in the regional trials under STRINF and STRNON conditions at Abuja and Mokwa, 2010–2021.**

| Trait | Stage | Unit | Description |
|---|---|---|---|
| Grain yield (YLD) | Harvest | Kg/ha | Computed from the weight of ~~the~~ shelled grain adjusted to 80% shelling percentage and corrected for 15% moisture content |
| Days to 50% anthesis (DA) | Flowering | Days | Days ~~count~~ from planting to 50% pollen shed ~~from planting~~ |
| Days to 50% silking (DS) | Flowering | Days | Days ~~count~~from planting –to 50% silk emergence ~~from planting~~ |
| Anthesis-silking interval (ASI) | Flowering | Days | The time interval between 50% anthesis and silking |
| Ears per plant (EPP) | Post-flowering | Numeric | Calculated by dividing the number of harvested ears by the number of plants harvested per plot |
| Ear aspect (EASP) | Harvest | Scale | Scored on a scale of 1 to 9, with 1 denoting ears that were clean,~~are~~ tidy, uniform, big, and full, and 9 denoting ears with undesirable characteristics |
| Emerged *Striga* plant at 8 and 10 WAP (ESP8 and ESP10) | Post-flowering | Count | The numbers of Striga plants that were counted at 8 and 10 WAP in the Striga-infested plots |
| *Striga* damage ratings at 8 and 10 WAP (SDR8 and SDR10) | Post-flowering | Scale | Scored on per plot basis on a scale of 1 to 9 where 1 = no damage, indicating normal plant growth and high resistance, and 9 = complete collapse or death of the maize plant |

**Formatted:** Line spacing: Double

**Formatted:** Line spacing: Double

**Formatted:** Line spacing: Double

**Formatted:** Line spacing: Double

**Formatted:** Line spacing: Double

**Formatted:** Line spacing: Double

**Formatted:** Font: Italic

**Formatted:** Line spacing: Double

**Formatted:** Font: Italic

**Formatted:** Line spacing: Double

**Formatted:** Line spacing: Double

## ~~2.4.~~ Statistical Analyses

The historical data used in the ~~current~~ analysis of the present study came from trials conducted under Striga-infested (STRINF) and *Striga* non-infested (STRNON) conditions. The analysis ~~procedure~~ followed~~s~~ ~~these~~ the following steps:

~~In t~~The first ~~stage~~step, ~~we conducted~~involved environment-level analyses ~~where~~ with the environment is defined as the combination of trial name, location, year, and condition~~.,~~ W~~we~~e used ~~a~~ the linear mixed model with the hybrids ~~treated~~ considered as random effects to estimate the broad-sense heritability a measure of experimental repeatability[25]. Trials with heritability values less than 0.1 were excluded from the estimated ~~of~~ genetic gains. After excluding the environments with heritability estimates less than 0.1, another linear mixed model (equation 1) was fitted ~~with~~ for the hybrids treated as a fixed effect to estimate the Best Linear Unbiased Estimates (BLUEs), and weight (inverse of squared standard error) for each hybrid ~~to be~~ used for ~~in~~ the second stage of the analyses as follows:~~.~~

$$y_{ikj} = \alpha + h_i + \varphi_k + \rho_j + \varepsilon_{ikj} \qquad (1)$$

where $y_{ikj}$ represents the yield value of the $i^{th}$ hybrid in the $k^{th}$ block and the $j^{th}$ replication. The overall mean is denoted by $\alpha$, the random effect of the $a^{th}$ hybrid is represented by $h_i$ , $\varphi_k$ is the effect of $b^{th}$ replication, $\rho_j$ is the effect of the kth block, and $\varepsilon_{ikj}$ is the random residual effect attributed to the yield value of the $i^{th}$ hybrid in the $k^{th}$ block and in the $j^{th}$ replication that follows the normal distribution $N(0,\ \sigma_e^2)$.

Secondly, we used the model represented by equation 2 [21] to analy~~se~~ze the BLUE values of grain yield and other traits obtained from equation 1 under STRINF and STRNON conditions as proposed by [21]

**Formatted:** Font: 16 pt

**Formatted:** Indent: Left: 0.75", Line spacing: Double, No bullets or numbering

**Formatted:** Line spacing: Double

**Formatted:** Font: Italic

$$Y_{ijklm} = \mu + \zeta_i + \eta_m + \lambda_{jlm} + \beta_{kjlm} + (\zeta\eta)_{im} + \xi_{lm} + \varepsilon_{ijklm} \qquad (2)$$

**Formatted:** Indent: Left: 1", Line spacing: Double

**Formatted:** Line spacing: Double

Where $Y_{ijklm}$ is the BLUE value of yield of the $i^{th}$ hybrid in the $k^{th}$ block, in the $j^{th}$ replication, in the $l^{th}$ location and $m^{th}$ year. The overall mean is represented by $\mu$. The effect of the $i^{th}$ hybrid is denoted by $\zeta_i$ and the effect of the $m^{th}$ year is represented by $\eta_m$; $\lambda_{jlm}$ represents the effect of the $j^{th}$ replication in the $l^{th}$ location and the $m^{th}$ year. Also, $\beta_{kjlm}$ is the effect of the $k^{th}$ block in $j^{th}$ replication in the $l^{th}$ location and $m^{th}$ year. The interaction effect between the $(im)^{th}$ hybrid and year is represented by $(\zeta\eta)_{im}$, while $\xi_{lm}$ is the effect of location $l$ within year $m$; $\varepsilon_{ijklm}$ is the residual, attributable to the combined effects of within-trial error and hybrid x location within-year interaction. Heterogeneity in the residual variances was assumed. Thus, we fitted a distinct residual variance for each condition for every combination of location and year.

Lastly, we used the weighted regression to examine trends (realized genetic gain) in the data ~~in~~ from the second stage of the analyses. The BLUEs of the hybrids and their year of origin in each condition were used as dependent and independent variables in the regression analyses respectively. We determined the percentage of each condition's genetic gain that could be attributed to genetic factors. The process involved calculating the ratio of the second stage's weighted regression slope plus the regression's y-intercept, and the product of the slope and year of the initial testing. The p-value $< 0.05$ for each trait from the summary of the weighted regression model was considered significant. The Best Linear Unbiased Predictions (BLUPs) yield of the hybrids in each environment from the fir st stage was visualized using the Boxplot. ~~Equally~~Similarly, the repeatability estimate of each trait over the years by location and

condition was presented using a scatter plot. All analyses were performed in R [26] using the ASReml package[27].

Formatted: Font: 18 pt

# 3. Results

Formatted: Indent: Left: 0.5", Line spacing: Double, No bullets or numbering

## 3.1 Genetic gains in grain yield and *Striga* adaptive traits

Formatted: Font: 16 pt

Formatted: Line spacing: Double

During the 12 years of development of the early maturing hybrids and genetic improvements, significant annual gains in grain yield of 3.40 and 3.71 % with changes of 76.87 and 127.02 kg ha$^{-1}$ yr$^{-1}$ were recorded under STRINF and STRNON conditions, respectively. The significant increases in grain yield were associated with significant increases of 1.84 and 1.79 % in ears per plant and significant decreases of 1.31 and 1.75 % in the ear aspect under STRINF and STRNON conditions, respectively. Under the STRINF condition, significant genetic gains of -3.04 and -2.25 % in *Striga* damage rating at 8 and 10 WAP, respectively, and -1.27 % in the number of

emerged *Striga* plants at 10WAP were obtained (Table 2). However, there were no significant gains in anthesis silking interval under STRINF environments and days to anthesis and silking under STRINF and STRNON conditions (Table 2). Additionally, the regression line considering the hybrid's first year of evaluation (independent variable) and traits (dependent variable) showed a positive linear relationship for grain yield and EPP and a negative linear relationship for ESP10, SDR10, and EASP. The breeding period under both conditions did not affect the number of days to flowering and ASI across STRINF and STRNON conditions (Figure 1a-1f).

**Formatted:** Font: Bold

**Table 2: Percent genetic gains in grain yield and other *Striga*-adaptive traits of maize hybrids evaluated under STRINF and STRNON conditions in Nigeria between 2010 –2021.**

| Trait | Management | % Genetic Gain (yearr$^{-1}$) | slope | slope.sd | intercept |
|---|---|---|---|---|---|
| YLD | STRINF | 3.40** | 76.87 | 13.32 | -152175.53 |
| | STRNON | 3.71** | 127.02 | 21.65 | -252000.76 |
| DA | STRINF | 0.12ns | 0.17 | 0.03 | -283.37 |
| | STRNON | 0.11ns | 0.06 | 0.04 | -63.30 |
| DS | STRINF | 0.10ns | 0.05 | 0.03 | -52.78 |
| | STRNON | 0.11ns | 0.06 | 0.05 | -72.47 |
| ASI | STRINF | -0.60ns | -0.01 | 0.02 | 32.56 |
| | STRNON | -5.62** | -0.13 | 0.02 | 272.06 |
| EASP | STRINF | -1.31** | -0.07 | 0.01 | 145.94 |
| | STRNON | -1.75** | -0.08 | 0.02 | 167.23 |
| EPP | STRINF | 1.84** | 0.01 | 0.00 | -25.00 |
| | STRNON | 1.79** | 0.01 | 0.00 | -26.62 |

**Formatted:** Line spacing: Double

**Formatted:** Line spacing: Double

**Formatted:** Line spacing: Double

**Formatted:** Line spacing: Double

**Formatted:** Line spacing: Double

**Formatted:** Line spacing: Double

| | | | | | |
|---|---|---|---|---|---|
| ESP8 | STRINF | -0.97ns | -0.31 | 0.22 | 653.83 |
| ESP10 | STRINF | -1.27* | -0.45 | 0.20 | 948.38 |
| SDR8 | STRINF | -3.04** | -0.16 | 0.02 | 328.14 |
| SDR10 | STRINF | -2.25** | -0.13 | 0.02 | 274.04 |

Sd:standard deviation; ** : Significant; ns : Not Significant; YLD: grain yield; DA: days to 50% anthesis; DS: days to 50% silking; ASI: anthesis-silking interval; EPP: ears per plant; EASP: ear aspect; ESP8: emerged *Striga* plant at 8 WAP; ESP10: emerged *Striga* plant at 10 WAP; SDR8: *Striga* damage ratings at 8 WAP; SDR10: *Striga* damage ratings at 10 WAP

Formatted: Line spacing: Double
Formatted: Line spacing: Double
Formatted: Line spacing: Double
Formatted: Line spacing: Double
Commented [A5]: Provide a clear legend below the table to define all abbreviated terms.
Commented [AAM(6R5]: Clear legend provided
Formatted: Line spacing: Double
Formatted: Font: 16 pt

## 3.2 Grain yield performance of early maturing maize hybrids evaluated in the regional trials across STRINF and STRNON conditions, 2010 –2021.

In the present study, three types of hybrids were evaluated, MST, SCO and LOC. The LOC cultivars comprised five hybrids, among them three common cultivars were evaluated over the period of 12 years. Additionally, the MST hybrids were evaluated for 2-6 years making the data well connected to allow an estimate of realized genetic gains. We assessed the yield gains under STRINF and STRNON conditions by comparing the MST hybrids' average grain yield performance to the SCO and LOC hybrids (Figure 2). The results showed that, under STRINF, the mean grain yield of MST hybrids ranged from 1091 kg ha$^{-1}$ for the hybrid developed in 2012 to 4287 kg ha$^{-1}$ for the hybrid developed in 2018. Similarly, the mean grain yield for SCO hybrids varied from 965 kg ha$^{-1}$ to 2964 kg ha$^{-1}$ and LOC hybrids varied from 1914 kg ha$^{-1}$ to 2644 kg ha$^{-1}$ (Supplementary Table

S1). ~~Contrarily~~On another hand, ~~the~~ mean grain yield of the hybrids evaluated under STRNON conditions ranged from 2268 kg $ha^{-1}$ to 6861 kg $ha^{-1}$ for the MST hybrids and from 3590 kg $ha^{-1}$ to 6644 kg $ha^{-1}$ for SCO hybrids while 2392 kg $ha^{-1}$ to 4088 kg $ha^{-1}$ were recorded for LOC hybrids. Generally, the MST hybrids had higher mean grain yields than the SCO and the LOC hybrids under STRINF and STRNON conditions, (Figure 2).

## 3.3 Repeatability estimates of the STRINF and STRNON trials

The results of the repeatability estimates of the trials conducted in the STRINF and STRNON conditions from 2010 to 2021 are shown in Table 3 and Figure 3a–f. Grain yield had repeatability estimates ranging from 0.00 for the a trial conducted under STRINF condition at Mokwa in 2016 to 0.91 for the STRINF trial evaluated at Abuja during the 2017 growing season. Among the trials evaluated in Abuja and Mokwa under STRINF and STRNON conditions, only three trials (about 8 %) possessed had repeatability estimates of less than 30% for grain yield, about 92% of the trials had repeatability greater than 30 % for grain yield (Figure 3). Similarly, days to anthesis had the repeatability estimates varied varying from 0.34 at Mokwa under STRINF condition in 2010 to 0.97 at Abuja in 2017 under STRINF condition. Days to silking had had repeatability estimates were observed in days to silking and variedvaried from 0.59 under STRINF condition at Mokwa in 2010 to 0.95 at Abuja in 2017 under STRINF condition. Anthesis silking interval, ear aspect and ears per plant have had repeatability ranging from 0.00 at Mokwa under STRNON condition in 2020 to 0.81 at Abuja under STRINF condition in 2016 while, repeatability estimates for ear aspect -varied from 0.00 at Mokwa under STRNON condition in 2015 to 0.92 at Mokwa under STRINF condition in 2020 and ears per plant had repeatability varying from 0.00 at Mokwa under STRNON condition in 2015 to 0.84 at Abuja under STRINF condition in 2019 (Table 3, figure 3). Furthermore, repeatability estimates under SRTINF environments varied from 0.10 in 2010 to 0.82 in 2020 at Abuja for ESP8 and from 0.00 in 2015 to 0.82 in 2020 at Abuja for ESP10 while 0.40 in 2010 at Abuja to 0.93 in 2020 at Mokwa for SDR8 and from 0.52 in 2016 to 0.95 at Mokwa in 2020 for SDR10 (Table 3, Figure 3).

**Formatted:** Font: 16 pt

**Formatted:** Line spacing: Double

**Commented [A7]:** Not attached. No figure or table showing performance of varieties (MST, SCO and LOC). Please attach

**Commented [AAM(8R7]:** Figures were submitted separately to the PLOS ONE webpage

**Table 3. Repeatability estimate of grain yield and other *Striga*-adaptive traits evaluated under STRINF and STRNON conditions at Mokwa and Abuja, ~~between~~ 2010 –2021**

| Trait | | Repeatability | Year | Location | Management |
|---|---|---|---|---|---|
| YLD | minimum | 0.00 | 2016 | Mokwa | STRINF |
| | maximum | 0.91 | 2017 | Abuja | STRINF |
| DA | minimum | 0.34 | 2010 | Mokwa | STRINF |
| | maximum | 0.97 | 2017 | Abuja | STRINF |
| DS | minimum | 0.59 | 2010 | Mokwa | STRINF |
| | maximum | 0.95 | 2017 | Abuja | STRINF |
| ASI | minimum | 0.00 | 2020 | Mokwa | STRNON |
| | maximum | 0.81 | 2016 | Abuja | STRINF |
| EASP | minimum | 0.00 | 2015 | Mokwa | STRNON |
| | maximum | 0.92 | 2020 | Mokwa | STRINF |
| EPP | minimum | 0.00 | 2015 | Mokwa | STRNON |
| | maximum | 0.84 | 2019 | Abuja | STRINF |
| ESP8 | minimum | 0.10 | 2010 | Abuja | STRINF |
| | maximum | 0.82 | 2020 | Abuja | STRINF |
| ESP10 | minimum | 0.00 | 2015 | Abuja | STRINF |
| | maximum | 0.82 | 2020 | Abuja | STRINF |
| SDR8 | minimum | 0.40 | 2010 | Abuja | STRINF |
| | maximum | 0.93 | 2020 | Mokwa | STRINF |
| SDR10 | minimum | 0.52 | 2016 | Mokwa | STRINF |

**Formatted:** Font: Bold

**Formatted:** Line spacing: Double

**Formatted:** Line spacing: Double

**Formatted:** Line spacing: Double

**Formatted:** Line spacing: Double

**Formatted:** Line spacing: Double

**Formatted:** Line spacing: Double

**Formatted:** Line spacing: Double

**Formatted:** Line spacing: Double

**Formatted:** Line spacing: Double

**Formatted:** Line spacing: Double

| | | | | |
|---|---|---|---|---|
| maximum | 0.95 | 2020 | Mokwa | STRINF |

YLD: grain yield; DA: days to 50% anthesis; DS: days to 50% silking; ASI: anthesis-silking interval; EPP: ears per plant; EASP: ear aspect; ESP8: emerged *Striga* plant at 8 WAP; ESP10: emerged *Striga* plant at 10 WAP; SDR8: *Striga* damage ratings at 8 WAP; SDR10: *Striga* damage ratings at 10 WAP

# ~~4.~~ Discussion

In addressing the challenges of chronic food insecurity, climate change, environmental sustainability, and improving the economic well-being of farmers in SSA, we used 12 years of historical data to explore three aspects:~~.~~ i) Accelerated genetic gains in grain yield and *Striga* resistance indicator traits ~~in~~ of early maturing tropical maize hybrids~~,~~ ~~to enable plant breeders to justify~~to explore the most effective use of the genetic variability ~~in~~ of early maturing tropical maize populations of IITA. ii) Compare the *performance* of *Striga*-tolerant hybrids developed in the breeding program of IITA to marketed farmer-preferred cultivars and hybrids developed by multinational seed companies and released in many African countries~~, thus~~ in an effort to justify~~ing~~ the program's resource allocation. iii) Assess the repeatability of the trials in the exploration of the ~~hybrids'~~ genotypic potential of the hybrids ~~in both~~under STRINF and STRNON conditions~~, hence, this allows plant breeders~~ in an effort to develop maize hybrids that could withstand the devastating effects of *Striga* infestation in *Striga*-prone environments.

**Formatted:** Line spacing: Double

**Formatted:** Font: 16 pt

**Formatted:** Normal, Line spacing: Double, No bullets or numbering

**Formatted:** Line spacing: Double

**Formatted:** Font: Italic

**Formatted:** Font: Italic

These, in turn, support rural development and poverty alleviation.

## Realized genetic gains in grain yield and othe *Striga* adaptive traits

Towards the efforts to make appropriate adjustments in the IITA-MIP , there is need for the investigation of the genotypice potential of the program and/or introduce and introgression of genetic variability into the breeding populations, as well as accelerate the rate of genetic advancement in aof the breeding program. In theThe results of the present study, revealed that significant progress was attainedhad been made during the past 12 years of genetic enhancements with annual gains in grain yield reaching 3.40 % $yr^{-1}$ and 3.71 % $yr^{-1}$ under STRINF and STRNON conditions, respectively. The gains are were associated with relative changes of 76.87 kg $ha^{-1}$ $yr^{-1}$ and 127.02 kg $ha^{-1}$ $yr^{-1}$ under STRINF and STRNON conditions, respectively. The progress attained in the present study was considerably higher compared to the annual genetic gains in grain yield of 2.91 % $yr^{-1}$ and 1.33 % $yr^{-1}$ archieved under low and high N environments, respectively, [10], 1.93 % $yr^{-1}$ under drought and well-watered conditions[15]-, and 0.74 % $yr^{-1}$ and 1.47 % $yr^{-1}$ under drought and full irrigation, respectively [19]. Additionally, [28] assessed the genetic gains in grain yield and other traits of the early maturing maize hybrids developed during the period 2000–2018 by International Maize and Wheat Improvement Center (CIMMYT) Southern African breeding program and reported annual yield gains of 2.21%, 2.13%, 1.64% and 1.78%, under low N, managed drought, random stress and optimum

Formatted: Font: 16 pt

Formatted: Font: 10 pt

conditions. However, (Badu-Apraku et al., 2020) reported genetic gains of 4.82 % $yr^{-1}$ and 1.24 % $yr^{-1}$ under STRINF and STRNON conditions, respectively in early maturing maize hybrids, ~~and~~ while [19]reported genetic gains of 2.58 % $yr^{-1}$ and 2.30 % $yr^{-1}$ under STRINF and STRNON conditions, respectively, in late-maturing maize hybrids. This implied that under *Striga* infestation, the hybrids responded to selection more favorably than under drought or low N ~~stresses~~conditions. This ~~might~~ could be attributed to the fact that ~~be because~~under artificial *Striga* infestated condition~~ion~~ the trials ~~are~~ were conducted ~~in~~ under more controlled environments ~~than under~~compared to drought and low N stress conditions, allowing for better accuracy in the selection process. ~~In addition~~Additionally, the *Striga* resistance may be more oligogenic than polygenic which is ~~mostly found in~~more commonly observed under drought and low N tolerance. Since the artificial *Striga* infestation process utilized for the development of inbred lines and the evaluation of hybrids has remained unchanged for the p~~l~~ast two decades, it could have continuously resulted in high levels of precision, which ~~had a~~might have major impact on the ~~achieving~~ achievement of a higher rate of genetic gain in grain yield under STRINF. Alternatively, it could be because more traits ~~are observed~~were studied during ~~a~~ the *Striga* infestation trials than during ~~a~~ the drought and~~or~~ low N trials, which could have aided breeders in precisely selecting the most promising hybrids. Furthermore, the low yield improvements observed under drought or low N stresses may be related to the adoption of *Striga*-resistant populations enhanced through $S_1$ recurrent selection without the infusion of tropical donor lines possessing superior genes for drought or low N tolerance as parents of the MST hybrids of IITA-MIP[19]. The significant increases in grain yield were associated with significant increases of 1.84 % $yr^{-1}$ and 1.79 % $yr^{-1}$ in ears per plant and significant decreases of 1.31 % $yr^{-1}$ and 1.75 % $yr^{-1}$ in the ear aspect under STRINF and

STRNON conditions, respectively. Additionally, the increases in grain yield under STRINF conditions were accompanied by a significant decrease of 3.04 % $yr^{-1}$, 2.25 % $yr^{-1}$, and 1.27 % $yr^{-1}$ in *Striga* damage ratings at 8 and 10 WAP and the number of emerged *Striga* plants at 10 WAP, respectively. Similar results were obtained by (Badu-Apraku et al., 2023; Menkir et al., 2022). Interestingly, the 12 years breeding period did not affect the flowering period of the early-maturing maize hybrids.

## Comparative performance of the hybrids

The success of the a breeding program depends on the establishment of the right selection criteria and the capacity to select hybrids that are superior in performance and/or competitive in grain yields under stressful conditions and across diverse environments. The comparative performance of MST hybrids to LOC and SCO hybrids across both STRINF and STRNON conditions showed revealed a clear distinction between the hybrids of the three groups. The MST hybrids performed exceptionally well in both STRINF and STRNON conditions. The result confirmed that MST with improved levels of *Striga* resistance outperformed those of SCO and LOC hybrids in contrasting environments. Our findings suggested that the breeding strategies, including recurrent selection, backcrossing, hybridization, evaluation, and selection, used employed to develop the MST hybrids during the 12-year breeding period were highly effective. This implied that MST hybrids possessed favorable genes that madeke them display better performers than the SCO and LOC hybrids. Therefore, the accumulation of new favorable alleles through rapid breeding cycles is was one of the possible scenarios that significantly increases the rate of genetic gains.

**Formatted:** Font: 16 pt

## Assesment of repeatability

Evaluating the repeatability of the STRINF and STRNON trials for assessing and exploring the ~~hybrids'~~ genotypic potential of the hybrids was ~~a~~ the focus of the present study. Unreliable or inconsistent repeatability estimates of the trials could have hindered the progress of accelerated genetic gains, as researchers could have wasted precious time and resources building on flawed or irreproducible findings. Reliable methodologies and experimental designs might have contributed to the overall quality of the scientific experiments. Rigorous methods help ensure that the results are a true reflection of the phenomenon under investigation, rather than being influenced by confounding variables or errors. In ~~summary~~a nutshell, repeatability ~~is~~ was essential in the assessment of the trials because it ensured~~s~~ the accuracy, consistency, trustworthiness, and validity of the trials. When the random error ~~is~~ was restricted, the variations observed in the trial ~~would~~ resulted solely in significant genotypic differences, environment, and genotype × environment (G×E) interactions. Nevertheless, the overall observed variation in the experiment was considerably influenced by genotype, environment, and G×E ~~G*E~~ interactions, determining the repeatability of the trial, ~~taking into account~~considering the combined effects of genotype, environment, G×E~~G*E~~ interactions, and random errors. Therefore, the disparities in the repeatability estimates of the trials conducted over a long period may be mostly due to the experimental procedures used/random error. We estimated the repeatability of the STRINF and STRNON trials conducted between 2010 and 2021 for each of the measured traits.

The highest repeatability was observed ~~in~~ for ears per plant and flowering dates (Figure 3), indicating that these traits would not be ~~largely~~ influenced largely by the

**Formatted:** Font: 16 pt

**Commented [A9]:** Are you sure? Please refer to your table

environment and the~~,~~ G×E ~~G*E~~ interactions. This suggested that flowering dates and ears per plant could be used to evaluate the effectiveness of the STRINF and STRNON trials in the exploration of the genotypic potential of the hybrids. However, the lowest average repeatability was observed ~~in~~ with the emerged *Striga* plant at 8 and 10 WAP. Thus, the emerged *Striga* plant at 8 and 10 WAP should not be used to determine the efficiency of the STRINF and STRNON trials in the exploration of the ~~hybrids'~~ genotypic potential of the hybrids. This is not surprising because the maize hybrids had different levels of stimulations ~~of~~ by the strigolactones, which ~~may lead~~have led to uneven germination of *Striga* seeds and their attachments to the host[30] . Additionally, a combination of factors encountered during trial evaluation at ~~each~~ the different locations ~~concerning~~ including distinct soil properties, nutrient contents, and pH levels, could have affected the emergence of the *Striga* plant [31]. Generally, a good level of repeatability was observed in the early maturing maize trials conducted under STRINF and STRNON conditions between the 2010 and 2021 growing seasons, indicating the potential of the trials in the identification of promising hybrids for commercialization in the SSA for increased food security and poverty alleviation.

**Commented [A10]:** Please check values in table 3

**Commented [A11R10]:** What of yield (0.00)?, EPP?

**Commented [AAM(12R10]:** Checked and corrected

**Formatted:** Font: 16 pt

# ~~5.~~ Conclusion

**Formatted:** Indent: Left: 0.5", Line spacing: Double, No bullets or numbering

The present research study used 12 years of historical data to analyze analyse the genetic gains in grain yield and associated changes in yield-related traits under STRINF and STRNON conditions and to estimate the repeatability of the trialsof the studied traits. The results showed significant progress in grain yield, with AAannual genetic gains of 3.40% yr$^{-1}$ and 3.71 % yr$^{-1}$ were obtained under STRINF and STRNON conditions, respectively. The flowering dates and ears per plant had the highest repeatability estimates. Ththeose traits have the lowest multi-dimensionalities, in estimated repeatability independent of environment and were not influenced and as such suitable for assessing the trials repeatabilityby environment, and G×E interactions. However, The emerged *Striga* plant at 8 and 10 WAP were identified as unsuitable had the highest multi dimensionality, suggesting that these traits for should not be used to determininge the efficiency of the trials particularly in exploring the genotypic potential of the hybrids. Significant genetic gains had been made and a good level of repeatability was observed in the early maturing maize trials., This indicates thating the quality of the STRINF and STRNON trials was adequate in an for identification exploration of outstanding hybrids for commercialization in the SSA to mitigate for food insecurity and enhance poverty alleviation.

The MST hybrids, with improved *Striga* resistance, outperformed the LOC and SCO hybrids in under both STRINF and STRNON conditions. The breeding strategies used during the 12-year breeding period were effective, suggesting that MST hybrids possessed favourable genes that contributed to their better performance. The flowering dates and ears per plant possess had the highest repeatability estimates., theese traits have the lowest multi-dimensionalities in estimated repeatability and were not largely influenced by environment, and G×E G*E interactions. However, the emerged *Striga* plant at 8 and 10 WAP had the highest multi-dimensionality, suggesting that these traits should not be used

**Formatted:** Line spacing: Double

**Commented [A13]:** Conclusion should address the objectives of study

**Commented [AAM(14R13]:** Modified to address the objectives

to determine the efficiency of the trials in exploring the hybrids' genotypic potential of the hybrids. ignificant genetic gains had been made and that a good level of repeatability was observed in early maturing maize trials, indicating the quality of the STRINF and STRNON trials in an exploration of outstanding hybrids for commercialization in the SSA for food security and poverty alleviation.

Commented [A15]: Conclusion should address the objectives of study

Formatted: Font: 16 pt

Formatted: Indent: Left: 1", Line spacing: Double

## Data availability statement

Formatted: Line spacing: Double

The raw data analyzed in this manuscript were available in IITA CKAN resporotoryrepository online at: https://doi.org/10.25502/8a4f-h291/d

## List of figures

Formatted: Indent: Left: 0", Line spacing: Double

Figure1a–f. Regression line of grain yield and other Striga adaptive traits (dependent variable) on year of development (independent variable) of hybrids evaluated in regional trials under STRINF conditions at Abuja and Mokwa, 2010 – 2021.

Figure 2. Box plot of the average grain yield of the MST, SCO, and LOC early maturing maize hybrids evaluated in the regional trials under STRINF and STRNON conditions at Abuja and Mokwa, 2010 – 2021.

Figure 3a–f. Scattered plot of the repeatability estimates for grain yield and other Striga adaptive traits evaluated under STRINF and STRNON conditions at Abuja and Mokwa 2010 –2021.

Formatted: Font: Bold

Formatted: Font: Bold

Formatted: Font: Bold

Formatted: Font: Bold

Formatted: Font: 16 pt

Formatted: Line spacing: Double

## Supporting Information

Table S1: Mean grain yield and other Striga adaptive traits, year of first testing in the regional trial of the multiple stress tolerant hybrids under STRINF condition in Nigeria, 2010 –2021; Table S2: Description of test locations used for evaluation of early maize hybrids under STRINF condition in Nigeria, 2010 –2021

## ~~Funding~~

~~This research was supported by the Bill & Melinda Gates Foundation [OPP1134248] under the Accelerated Genetic Gains (AGG) and Stress Tolerant Maize for Africa (STMA) Projects.~~

## Acknowledgments

The authors are grateful to the IITA-MIP, EBS and Biometric Unit technical, field, and administrative staff for providing technical, field, and administrative assistance.

Formatted: Font: 16 pt

Formatted: Font: 16 pt

## Conflict of interest

The authors declare that there is no conflict of interest.

# Reference


[1] B. Badu-Apraku, G. B. Adu, A. M. Yacoubou, J. Toyinbo, and S. Adewale, “Gains in genetic enhancement of early maturing maize hybrids developed during three breeding periods under striga-infested and striga-free environments,” *Agronomy*, vol. 10, no. 8, 2020, doi: 10.3390/agronomy10081188.

[2] I. M. Mwangangi, L. Büchi, S. M. Haefele, L. Bastiaans, S. Runo, and J. Rodenburg, “Combining host plant defence with targeted nutrition: key to durable control of hemiparasitic Striga in cereals in sub-Saharan Africa?,” 2021. doi: 10.1111/nph.17271.

[3] I. C. Akaogu *et al.*, “Inheritance of Striga hermonthica adaptive traits in an early-maturing white maize inbred line containing resistance genes from Zea diploperennis,” *Plant Breeding*, vol. 138, no. 5, 2019, doi: 10.1111/pbr.12707.

[4] G. Ejeta, “The striga scourge in africa: A growing pandemic. In: Ejeta G, Gressel J, editors. Integrating New Technologies for Striga Control: Towards Ending the Witch-Hunt,” *World Scientific Publishing Company*, 2007.

[5] S. K. Kim, V. O. Adetimirin, C. Thé, and R. Dossou, “Yield losses in maize due to Striga hermonthica in West and Central Africa,” *Int J Pest Manag*, vol. 48, no. 3, 2002, doi: 10.1080/09670870110117408.

[6] B. A. Kountche *et al.*, “Suicidal germination as a control strategy for Striga hermonthica (Benth.) in smallholder farms of sub-Saharan Africa,” *Plants People Planet*, vol. 1, no. 2, 2019, doi: 10.1002/ppp3.32.


Formatted: Font: 16 pt, Bold

Formatted: Justified, Line spacing: 1.5 lines

Formatted: Font: Palatino Linotype

Formatted: Font: Palatino Linotype, 11 pt

Formatted: Font: Palatino Linotype

[7] S. K. Kim, IITA, ICRISAT, and IDRC, "Breeding maize for striga tolerance and the development of a field infestation technique," *Combating Striga in Africa*, 1988.

[8] R. O. Abdulmalik *et al.*, "Genetic gains in grain yield of a maize population improved through marker assisted recurrent selection under stress and non-stress conditions in West Africa," *Front Plant Sci*, vol. 8, 2017, doi: 10.3389/fpls.2017.00841.

[9] B. Badu-Apraku, M. A. B. Fakorede, and A. M. Abubakar, "Accelerated Genetic Gains in Early-Maturing Maize Hybrids following Three Periods of Genetic Enhancement for Grain Yield under Low and High Soil-Nitrogen Environments," *Plants*, vol. 11, no. 9, May 2022, doi: 10.3390/plants11091208.

[10] B. Badu-Apraku, M. A. B. Fakorede, and A. M. Abubakar, "Accelerated Genetic Gains in Early-Maturing Maize Hybrids following Three Periods of Genetic Enhancement for Grain Yield under Low and High Soil-Nitrogen Environments," *Plants*, vol. 11, no. 9, May 2022, doi: 10.3390/plants11091208.

[11] D. N. Duvick, "Genetic progress in yield of United States maize (Zea mays L.)," 2005.

[12] L. Echarte, L. Nagore, J. Di, M. Cambareri, M. Robles, and A. Della, "Grain Yield Determination and Resource Use Efficiency in Maize Hybrids Released in Different Decades," in *Agricultural Chemistry*, 2013. doi: 10.5772/55287.

[13] B. Masuka *et al.*, "Gains in maize genetic improvement in eastern and southern Africa: I. CIMMYT hybrid breeding pipeline," *Crop Sci*, vol. 57, no. 1, 2017, doi: 10.2135/cropsci2016.05.0343.

[14] A. Menkir and S. Meseka, "Genetic improvement in resistance to striga in tropical maize hybrids," *Crop Sci*, vol. 59, no. 6, 2019, doi: 10.2135/cropsci2018.12.0749.

[15] M. Oyekunle *et al.*, "Gains in grain yield of released maize (Zea mays L.) cultivars under drought and well-watered conditions," *Exp Agric*, vol. 55, no. 6, pp. 934–944, Dec. 2019, doi: 10.1017/S0014479719000048.

[16] B. Badu-Apraku, A. M. Abubakar, G. B. Adu, A.-M. Yacoubou, S. Adewale, and I. I. Adejumobi, “Enhancing Genetic Gains in Grain Yield and Efficiency of Testing Sites of Early-Maturing Maize Hybrids under Contrasting Environments,” *Genes (Basel)*, vol. 14, no. 10, p. 1900, Sep. 2023, doi: 10.3390/genes14101900.

[17] B. M. Prasanna *et al.*, “Genetic trends in CIMMYT’s tropical maize breeding pipelines,” *Sci Rep*, vol. 12, no. 1, Dec. 2022, doi: 10.1038/s41598-022-24536-4.

[18] L. A. Crespo-Herrera *et al.*, “Genetic yield gains in CIMMYT’S international elite spring wheat yield trials by modeling the genotype × environment interaction,” *Crop Sci*, vol. 57, no. 2, pp. 789–801, Mar. 2017, doi: 10.2135/cropsci2016.06.0553.

[19] A. Menkir *et al.*, “Estimating genetic gains for tolerance to stress combinations in tropical maize hybrids,” *Front Genet*, vol. 13, Dec. 2022, doi: 10.3389/fgene.2022.1023318.

[20] A. J. de la Vega, I. H. DeLacy, and S. C. Chapman, “Progress over 20 years of sunflower breeding in central Argentina,” *Field Crops Res*, vol. 100, no. 1, 2007, doi: 10.1016/j.fcr.2006.05.012.

[21] I. Mackay, A. Horwell, J. Garner, J. White, J. McKee, and H. Philpott, “Reanalyses of the historical series of UK variety trials to quantify the contributions of genetic and environmental factors to trends and variability in yield over time,” *Theoretical and Applied Genetics*, vol. 122, no. 1, 2011, doi: 10.1007/s00122-010-1438-y.

[22] H. P. Piepho and J. Möhring, “Selection in cultivar trials—Is it ignorable?,” *Crop Sci*, vol. 46, no. 1, 2006, doi: 10.2135/cropsci2005.04-0038.

[23] B. Badu-Apraku and M. A. B. Fakorede, *Advances in genetic enhancement of early and extra-early maize for sub-Saharan Africa.* 2017. doi: 10.1007/978-3-319-64852-1.

[24] S. K. Kim, A. Y. Akintunde, and P. Walker, “Responses of maize inbreds during development of Striga hermonthica infestation,” *Maydica*, vol. 44, no. 4, 1999.

[25] B. R. Cullis, A. B. Smith, and N. E. Coombes, "On the design of early generation variety trials with correlated data," *J Agric Biol Environ Stat*, vol. 11, no. 4, 2006, doi: 10.1198/108571106X154443.

[26] R Core Team., "R: A language and environment for statistical computing. R Foundation for Statistical Computing, Vienna, Austria.," *Open J Stat*, vol. 13, no. 02, 2023.

[27] a R. Gilmour, B. J. Gogel, B. R. Cullis, and R. Thompson, "ASReml user guide release 3.0," *VSN International Ltd*, 2009.

[28] A. Tarekegne *et al.*, "Genetic gains in early maturing maize hybrids developed by the International Maize and Wheat Improvement Center in Southern Africa during 2000–2018," *Front Plant Sci*, vol. 14, 2023, doi: 10.3389/fpls.2023.1321308.

[29] B. Badu-Apraku, G. B. Adu, A. M. Yacoubou, J. Toyinbo, and S. Adewale, "Gains in genetic enhancement of early maturing maize hybrids developed during three breeding periods under striga-infested and striga-free environments," *Agronomy*, vol. 10, no. 8, 2020, doi: 10.3390/agronomy10081188.

[30] M. Jamil, F. K. Kanampiu, H. Karaya, T. Charnikhova, and H. J. Bouwmeester, "Striga hermonthica parasitism in maize in response to N and P fertilisers," *Field Crops Res*, vol. 134, 2012, doi: 10.1016/j.fcr.2012.03.015.

[31] S.-K. Kim, "Horizontal resistance: core to a research breakthrough to combat Striga in Africa," *Integrated Pest Management Reviews*, vol. 1, no. 4, 1996, doi: 10.1007/bf00139766.

Formatted: Font: Palatino Linotype, 11 pt

Formatted: Line spacing: Double

**REBUTTAL TO REVIEWERS' COMMENTS ON THE MANUSCRIPT TITLED " REPEATABILITY AND GENETIC ADVANCES IN EARLY MATURING MAIZE HYBRID TRIALS CONDUCTED UNDER STRIGA-INFESTED AND NON-INFESTED CONDITIONS"**

**Response to Editor (Journal requirements)**

1. Please ensure that your manuscript meets PLOS ONE's style requirements, including those for file naming.

   **Response:** The manuscript has been modified to meet PLOS ONE's style requirements.

2. Please note that funding information should not appear in any section or other areas of your manuscript. We will only publish funding information present in the Funding Statement section of the online submission form. Please remove any funding-related text from the manuscript.

   **Response:** The funding-related text has been removed from the manuscript

3. We note that the grant information you provided in the 'Funding Information' and 'Financial Disclosure' sections do not match. When you resubmit, please ensure that you provide the correct grant numbers for the awards you received for your study in the 'Funding Information' section.

   **Response:** The funding information will be corrected upon resubmission of the revised manuscript.

4. Thank you for stating the following financial disclosure: "This research was supported by the Bill & Melinda Gates Foundation under the Accelerated Genetic Gains (AGG) and Stress Tolerant Maize for Africa (STMA) Projects."
   Please state what role the funders took in the study. If the funders had no role, please state: "The funders had no role in study design, data collection and analysis, decision to publish, or preparation of the manuscript." If this statement is not correct you must amend it as needed. Please include this amended Role of Funder statement in your cover letter; we will change the online submission form on your behalf.

   **Response:** The funder was the main donor of the AGG and STMA projects and also the payer of the article processing charge for the manuscript.

5. Thank you for uploading your study's underlying data set. Unfortunately, the repository you have noted in your Data Availability statement does not qualify as an acceptable data repository according to PLOS's standards. At this time, please upload the minimal data set necessary to replicate your study's findings to a stable, public repository (such as figshare or Dryad) and provide us with the relevant URLs, DOIs, or accession numbers

that may be used to access these data. For a list of recommended repositories and additional information on PLOS standards for data deposition, please see https://journals.plos.org/plosone/s/recommended-repositories

**Response:** The acceptable data repository according to PLOS's standards with DOIs have now been provided in the data availability statement of the manuscript

6. When completing the data availability statement of the submission form, you indicated that you will make your data available on acceptance. We strongly recommend all authors decide on a data sharing plan before acceptance, as the process can be lengthy and hold up publication timelines. Please note that, though access restrictions are acceptable now, your entire data will need to be made freely accessible if your manuscript is accepted for publication. This policy applies to all data except where public deposition would breach compliance with the protocol approved by your research ethics board. If you are unable to adhere to our open data policy, please kindly revise your statement to explain your reasoning and we will seek the editor's input on an exemption. Please be assured that, once you have provided your new statement, the assessment of your exemption will not hold up the peer review process.

   **Response:** The data have now been made available.

7. We note you have included a table to which you do not refer in the text of your manuscript. Please ensure that you refer to Table 1 in your text; if accepted, production will need this reference to link the reader to the Table.

   **Response:** We have made appropriate reference to Table 1 in the revised version of the manuscript

8. Please include captions for your Supporting Information files at the end of your manuscript, and update any in-text citations to match accordingly. Please see our Supporting Information guidelines for more information: http://journals.plos.org/plosone/s/supporting-information.

   **Response:** Captions for Supporting Information files have now been provided at the end of the manuscript

9. Please review your reference list to ensure that it is complete and correct. If you have cited papers that have been retracted, please include the rationale for doing so in the manuscript text, or remove these references and replace them with relevant current references. Any changes to the reference list should be mentioned in the rebuttal letter that accompanies your revised manuscript. If you need to cite a retracted article, indicate the article's retracted status in the References list and also include a citation and full reference for the retraction notice.

**Response:** The list of references has been reviewed to ensure that it is complete and correct. We observed that a reference was not cited appropriately which was taken care of. In addition, duplicate references have also been resolved in the revised version of the manuscript.

Responses to Reviewers' Comments

Reviewer #1: The quality of the manuscript is technically sound, and data addressed all objectives. However, some concerns are:

1. Results should be reported in the past tense.

**Response:** The results section of the manuscript has been reported in the past tense as requested.

2. Some minor grammatical errors need correction; a few have already been pointed out.

**Response:** We have revised the manuscript for grammatical modifications and sentence chronology to improve readability in the revised version of the manuscript as requested.

3. Figures addressing Objective 2 are missing.

**Response:** We apologize for this however all figures relevant to the work were submitted separately to the PLOS ONE webpage. We hope the editor can resolve this issue in the subsequent interactive review.

4. Please refer to Table 3 and recast your results on repeatability values for the traits.

**Response:** As requested, we have recast the results relating to Table 3 to ensure accurate interpretation for readers.

5. Indicate in the Materials and Methods section the method used for assessing the significance of values.

**Response:** The method used for assessing the significance of the values have been included in the Materials and Methods section as requested in the revised manuscript.

6. Title can be rephrased as "Repeatability and genetic gain"

**Response:** We appreciate your suggestion to improve the quality of the manuscript. The co-authors have several similar publications with titles including the word “genetic gains”. We would like to use the word “genetic advances” as a replacement for “genetic advancement” to provide a sense of uniqueness to the manuscript title.

7. Restructure your conclusions to address the objectives.
**Response:** We have restructured the conclusion section to focus on the objectives in the revised manuscript as requested.

Reviewer #2: The manuscript is well written except for few MINOR REVISIONS to be made before acceptance:

(1) The 'Introduction' section is too long and need to be shortened with good flow of the required information
**Response:** Thank you. We have revised the Introduction as requested.

(2) Correct as 'Materials and Methods'
**Response:** Material and Method were corrected as 'Materials and Methods' as requested.

(3) Present the coordinates of the test locations
**Response:** Coordinates of the test locations have been referenced in the revised manuscript as requested.

(4) Present the agro-climatic pattern of the different locations and years
**Response:** Thank you for your comment. The agro-climatic patterns of the two locations have been included as a supplementary file to be submitted alongside the revised manuscript.

(5) The discussion to be presented with suitable sub-headings for easy understanding to the readers
**Response:** Discussion has now been presented with suitable sub-headings as requested.
