# Supplementary material for: Repeatability and genetic advances in early maturing maize hybrid trials conducted under Striga-infested and non-infested conditions
Source: PLoS One. 2025 Apr 3;20(4):e0319353. doi: 10.1371/journal.pone.0319353 (PMC11967925; doi:10.1371/journal.pone.0319353)
Supplement: Attachment — Submitted filename: GG MS-final.docx [file pone.0319353.s002.docx]

**REPEATABILITY AND GENETIC ADVANCEMENT IN EARLY MATURING MAIZE HYBRID TRIALS CONDUCTED UNDER *STRIGA*-INFESTED AND NON-INFESTED CONDITIONS**

**Adamu Masari Abubakar^1&2^,** **Idris Ishola Adejumobi^1*^, Kayode Rapheal Fowobaje^1^, Ibnou Dieng^1^, Saminu Zakariya^2^, Simon Imoro^1^ and Baffour Badu-Apraku^1^,**

^1^International Institute of Tropical Agriculture (IITA), PMB 5320, Oyo Road, Ibadan, Oyo State, Nigeria; [a.abubakar@cgiar.org](mailto:a.abubakar@cgiar.org)* (A.A.M.); [i.adejumobi@cgiar.org](mailto:i.adejumobi@cgiar.org) (I.I.A.); [k.fawobaje@cgiar.org](mailto:k.fawobaje@cgiar.org) (K.R.F.); [i.dieng@cgiar.org](mailto:i.dieng@cgiar.org) (I.D.); [s.imoro@cgiar.org](mailto:s.imoro@cgiar.org) (S.I); [b.badu-apraku@cgiar.org](mailto:b.badu-apraku@cgiar.org) (B.B.A.)

^2^Department of Plant Science, Institute for Agricultural Research, Ahmadu Bello University, P.M.B. 104, Zaria, Kaduna State, Nigeria. [zacqkz@gmail.com](mailto:zacqkz@gmail.com) (S.Z.)

**Abstract:** In sub-Saharan Africa (SSA), maize (Zea mays L.) is both a cash crop and a significant staple. However, Striga infection constrains its production. A total of 159 hybrids in 21 international trials were evaluated under *Striga*-infested (STRINF) and non-infested (STRNON) conditions at Mokwa and Abuja, Nigeria, from 2010 to 2021. The data were used to (i) determine the genetic enhancements in grain yield and Striga adaptive traits and (ii) assess the repeatability of the trials in the exploration of the hybrid's genotypic potential. Significant annual gains in grain yield of 3.40% and 3.71% with changes of 76.87 and 127.02 kg ­ha^−1^ ­yr^−1^ were recorded under STRINF and STRNON conditions, respectively. The gains were associated with 3.04 and 2.25 % decreases in Striga damage rating at 8 and 10 weeks after planting (WAP), respectively, and 1.27 % in the number of emerged Striga plants at 10 WAP. The results indicated that ears per plant and flowering dates have the highest repeatability while emerged Striga plants at 8 and 10 WAP have the lowest repeatability. Generally, substantial progress has been attained and there is a good level of repeatability estimates in early maturing maize hybrid trials evaluated under STRINF and STRNON conditions, 2010 to 2021. Therefore, the breeding strategies adopted during the 12-year breeding period were effective, indicating the potential of the trials in the exploration of the hybrids' potential for commercialization in the SSA for food security and poverty alleviation.

**Keywords**: selection, genetic gains, *Striga* resistance, grain yield, cereal crops.

1. **Introduction**

Maize plays a particularly important role as a staple food in the diets of millions of people in Africa, especially in ensuring food security. Maize production and productivity are challenged by many constraints of which *Striga hermonthica* (purple witchweed), an obligate hemi-parasitic plant is one of the factors causing substantial loss to grain yield on farmers’ fields in sub-Saharan Africa (SSA) (Badu-Apraku et al., 2020). Even though *S. hermonthica* parasitizes the root systems of cereals, maize is however the most preferred among the cereal crops. Every cropping season, soil-borne *Striga* seeds germinate and attach to maize plant root systems, causing plant toxicity, yield reduction by siphoning water and nutrients from the host plant, and causing the death of the host plant in extreme cases. The outcome of this association is a significant reduction in the obtainable grain yield. *Striga* parasitism is more common in the Savanna region and results in food insecurity and rural poverty. *Striga*'s lifespan depends on its ability to siphon nutrients and water from host plants to promote its growth and development. The species have 5-7 days to establish themselves on a suitable host, otherwise, if the stored resource in the seeds is exhausted, the radicles will wither (Mwangangi et al., 2021)(Mwangangi et al., 2021) (Mwangangi et al., 2021). Following a successful attachment, *Striga* damages the host plant by penetrating and colonizing its root cells, lowering the host's productivity and function even before it emerges from the ground. *Striga* is a major threat to food security in SSA, where they negatively affect the growth of staple cereals crops. According to (Akaogu et al., 2019; Ejeta, 2007; Kim et al., 2002; Kountche et al., 2019),(Akaogu et al., 2019; Ejeta, 2007; Kim et al., 2002; Kountche et al., 2019),it causes yield losses of $7 to $10 billion worth and, could result in up to 100% losses in maize production, especially when the infestation is severe during the vegetative growth stage.

Breeding for *Striga* resistance has proven to be the most cost-effective, feasible, and long-term strategy for farmers with limited resources and who have an interest in protecting the environment. One of the main objectives of maize breeders in SSA is to develop *Striga*-resistant/tolerant and commercially desirable maize hybrids. The scientists of the International Institute of Tropical Maize Improvement Program (IITA-MIP) initiated *Striga* resistance research on maize in 1982 with most of the germplasm originating from the U.S. Corn Belt. Resistant inbreds and hybrids of IITA were first identified in 1983 from field testing at Mokwa in Nigeria(Kim et al., 1988). Since then, breeders of the maize improvement program (MIP) have been working steadily to develop maize varieties/hybrids with elevated levels of *Striga* tolerance to support smallholder farmers in the targeted production environments. In this regard, IITA-MIP in collaboration with the National Agricultural Research Scientist (NARS) through deliberate teamwork, has developed and released several high-yielding multiple stress-tolerant (MST) (i.e., resistant to *Striga* and tolerant to drought and low N) hybrids and open-pollinated varieties (OPVs), many of which are suitable for several agro-ecologies in SSA. For the past two decades, the IITA-MIP has been developing and deploying *Striga*-tolerant hybrids in regional trials to NARS in West and Central Africa (WCA) for evaluation and commercialization in the region. The hybrids in the regional trials are selected based on successful performance in at least three successive trial evaluations. The regional trials are an important vehicle for the exchange of germplasm among the IITA-MIP and the National Maize Programs of WCA. The regional trials allow the extensive testing of promising maize hybrids across a set of target environments. It offers the National Maize Programs of WCA the opportunity to select hybrids/OPVs with superior performance for further testing and release directly as a replacement for old and less-performing varieties or as source materials for their breeding programs. In addition to being dispatched to the NARS partners, IITA-MIP every year participates in the evaluation of these trials across multiple test environments (stress and non-stress). The *Striga* trials are being conducted at Mokwa and Abuja under *Striga*-infested (STRINF) and non-infested (STRNON) conditions.

Assessment of the breeding program through timely estimating genetic gains is important to determine the progress that has been made and the effectiveness of the breeding approach used over the years in achieving the required improvements to meet the present and future demands for maize. To demonstrate gains in grain yield and other agronomic attributes from selection, several studies comparing the performance of maize cultivars developed over time and evaluated in field trials conducted in common sets of environments have been documented(Abdulmalik et al., 2017; Badu-Apraku et al., 2022a, 2023a; Duvick, 2005; Echarte et al., 2013; Masuka et al., 2017; Menkir & Meseka, 2019; Oyekunle et al., 2019a) These studies have been conducted by choosing genotypes that are representative of the breeding era or cycle and evaluating them simultaneously in common sets of environments over the years. To report a few of the previous findings, (Menkir & Meseka, 2019) reported a yield gain of 3.2% with a mean increase of 93.7 kg ha^-1^ yr^-1^ under Striga-infested environments in a set of 32 late/intermediate maturing maize hybrids developed over three breeding periods. Badu-Apraku et al. (2023)evaluated 54 hybrids (18 each in a breeding period) developed during three periods of genetic enhancement and reported annual genetic gains from selection in grain yield of 84.72 kg ha^−1^ yr^-1^ (4.05%) and 61 kg ha^−1^ yr^-1^ (1.56%), under multiple-stress and non-stress environments, respectively. These studies necessitate a substantial resource investment particularly time, difficulty in producing, packaging, and dispatching the seeds, logistical difficulties when reassembling historical genotypes and genotypes developed years ago may perform differently in most recent years due to the climate change, where new genotypes will be favored. In addition, these studies do not facilitate monitoring of the effectiveness of breeding strategies to allow the exploration of weaknesses to make the necessary adjustments. However, the effects of different trial management techniques and varying climatic conditions on genetic trends are usually eliminated to provide the most unbiased estimates of genetic gain (Prasanna et al., 2022)

In estimating gains from selection over several breeding periods, numerous methodologies have been documented. (Crespo-Herrera et al., 2017) used the factor analytic model approach to estimate genetic gains by regressing the difference in mean grain yield of the highest-yielding varieties on the mean yield cultivars used as checks from 8 years of historical data of wheat and reported an annual genetic gain of 1.67% relative to the commercial check and 0.53% relative to the local check. Additionally, (Menkir et al., 2022a) used mixed model analysis in regional collaborative late maturing maize hybrid trials data conducted for 8 years and reported yield gain of 11.89 kg ha^-1^ yr^-1^ under managed drought stress, 86.60 kg ha^-1^ yr^-1^ under *Striga* infestation, 62.65 kg ha^-1^ yr^-1^ under full irrigation, 102.44 kg ha^-1^ yr^-1^ under *Striga*-free conditions, and 53.11 kg ha^-1^ yr^-1^ across rainfed environments. Breeding trials continuously take into account the dynamics of adding new genotypes and eliminating the subpar ones. As a result, many authors (de la Vega et al., 2007; Mackay et al., 2011; Menkir et al., 2022b; Piepho & Möhring, 2006) recommended the use of mixed linear model analysis to reliably estimate genetic gains for unbalanced data from multi-environment trials. Recently, One Consortium of International Agricultural Research Centers (CGIAR) has updated its recommendations, emphasizing the use of historical data sets to analyze genetic gain. The strategy is crucial to enable regular monitoring of breeding programs' progress and the efficacy of breeding methodologies for the development of superior genotypes.

The repeatability of the trial observed over the years may differ from that of other years and/or all years combined. The observed discrepancy could result from differences in climate conditions, trial management, or random chance due to the additive effect of the experimental errors. Sporadic assessment of the efficiency of the methodologies through evaluation of the repeatability and the estimates of the realized genetic gains over time in a breeding program is important. It allows evaluation of the progress, the approach, and the effectiveness of breeding methodologies for planning new strategies or adopting the method being used in the breeding program.

Early maturing *Striga*-tolerant/resistant hybrids were subsequently evaluated under artificial STRINF and STRNON conditions across several locations and seasons for the past two decades. Therefore, the data is suitable for assessing the genetic improvement and evaluation of the repeatability of the trials. Thus, the objectives of the present study were to; (i) determine the genetic improvement in grain yield and *Striga* adaptive traits associated with the changes and (ii) assess the repeatability of the trials in the exploration of the hybrid's genotypic potential in both STRINF and STRNON conditions. Information presented in this study would be crucial to guide breeders to assess the effectiveness of the breeding programs and correct if necessary and justify the resources investment in a breeding program.

2. **Material and Method**
   2.1. **Development of the genetic materials**

The early and extra-early breeding program of IITA started the inbreds development in 1994. The program aimed at the development of *Striga* resistant inbred lines, hybrids, and OPVs. Early maturing populations TZE-W Pop DT STR C0, TZE-Y Pop DT STR C0, TZE Comp 5-Y C6, and TZE-W Pop × 1368 STR were initially used as the sources for *Striga* resistance. These populations were valuable sources of genes for *Striga* resistance and drought tolerance and they have been used to develop several outstanding inbreds, hybrids, and OPVs in the early and extra-early breeding program. However, the levels of resistance to *Striga* in these populations were not as high as desired. In 2007, a program was started aimed at increasing the frequency of favorable alleles for *Striga* resistance and drought tolerance using the S_1_ family recurrent selection to introgressed genes from wild relative *Zea diploperennis*. This resulted in the development of new generations of outstanding populations that combined improved levels of resistance to *Striga* and tolerance to drought and low soil nitrogen. The approaches that have been utilized in the development of the hybrids used in the present study were described in detail by(Badu-Apraku & and Fakorede, 2017). In summary, the disserved germplasm sources were used to generate the S_1_ lines and advanced to the S_4_ stages of inbreeding through selfing. The lines were evaluated under artificial *Striga* infestation and induced moisture stress after each inbreeding cycle. At the S_4_ stage, 250–300 selected lines were crossed to broad-based testers to estimate the general combining abilities of the lines. Based on the performance in the test-crosses, 90–100 S_4_ lines were advanced to the S_6_ stage of inbreeding. The S_6_ lines were used in the development of the hybrids. Every set of hybrids developed, they were first evaluated in progeny trials from where successful candidates were advanced to the second testing stage of the progeny trial and then moved to preliminary trials. Based on the genotypes’ performances, successful candidates were advanced to the regional trials whose data were used for this study.

A total of 159, early maturing white MST hybrids (MST1–159) developed by IITA-MIP, 5 farmer-preferred hybrids marketed conventional *Striga*-resistant hybrids/cultivars (LOC 1–5), and 13 hybrids (SCO 1–13) supplied by multi-national seed companies and commercialized in many African countries were used in the present study. The detailed descriptions and the year of first testing in the regional trial of the hybrids are presented in Supplementary Table S1.

2.2. **Management of Field Trials**

In the present study, we used historical data from trials conducted under STRINF and STRNON conditions at Abuja and Mokwa in Nigeria, from 2010 to 2021 growing seasons. The characteristics of the two test environments and management of the trials under STRINF and STRNON conditions were described in details by (Badu-Apraku et al., 2023b). The alpha lattice design was employed for the trials containing more than twenty entries while RCBD was adopted for the trials that had not more than twenty entries. Each trial had three replications with a plot consisting of 2 rows, each 4 m long. The spacing between rows was 75 cm while plants within a row were 4 cm apart. Three seeds were placed into each hole and later thinned to two plants per hill resulting in a population density of 66,666 plants ha^-1^. The same hybrid was planted in two infested rows and two non-infested rows within each strip, planted exactly opposite each other and separated by a 1.5-meter alley. The artificial infestation was achieved by putting about 5000 germinable *Striga* seeds per hill followed by planting maize seeds into the same holes as described in detail by (Badu-Apraku & Fakorede, 2017). To subject the maize plants to stress and encourage the production of strigolactones that promote good germination of *Striga* seeds and attachment of *Striga* plants to the roots of maize plants in *Striga*-infested plots, fertilizer application was delayed till about 30 days after planting(S. K. Kim et al., 1999). The compound fertilizer NPK 15:15:15 was used to apply 30 kg ha^−1^ N, P, and K at 30 and 45 days after planting (DAP). Weeds other than *Striga* were removed by hand throughout the seasons.

2.3. **Traits Measured**

Table 1: Descriptions of the measured traits of early white maize hybrids evaluated in the regional trials under STRINF and STRNON conditionts at Abuja and Mokwa, 2010–2021.

| Trait | Stage | Unit | Description |
| --- | --- | --- | --- |
| Grain yield (YLD) | Harvest | Kg/ha | Computed from the weight of the shelled grain adjusted to 80% shelling percentage and corrected for 15% moisture content |
| Days to 50% anthesis (DA) | Flowering | Days | Day count to 50% pollen shed from planting |
| Days to 50% silking (DS) | Flowering | Days | Day count to 50% silk emergence from planting |
| Anthesis-silking interval (ASI) | Flowering | Days | The time interval between 50% anthesis and silking |
| Ears per plant (EPP) | Post-flowering | Numeric | Calculated by dividing the number of harvested ears by the number of plants harvested per plot |
| Ear aspect (EASP) | Harvest | Scale | Scored on a scale of 1 to 9, with 1 denoting ears that are tidy, uniform, big, and full, and 9 denoting ears with negative characteristics |
| Emerged Striga plant at 8 and 10 WAP (ESP8 and ESP10) | Post-flowering | Count | The numbers of Striga plants that were counted at 8 and 10 WAP in the Striga-infested plots |
| Striga damage ratings at 8 and 10 WAP (SDR8 and SDR10) | Post-flowering | Scale | Scored on per plot basis on a scale of 1 to 9 where 1 = no damage, indicating normal plant growth and high resistance, and 9 = complete collapse or death of the maize plant |

2.4. **Statistical Analyses**

The historical data used in the current analysis came from trials conducted under Striga-infested (STRINF) and non-infested (STRNON) conditions. The analysis procedure follows these steps:

In the first stage, we conducted environment-level analyses where the environment is defined as the combination of trial name, location, year, and condition, we used a linear mixed model with the hybrids treated as random effects to estimate the broad-sense heritability a measure of experimental repeatability (Cullis et al., 2006). Trials with heritability values less than 0.1 were excluded from the estimate of genetic gains. After excluding the environment with heritability less than 0.1, another linear mixed model (equation 1) was fitted with the hybrids treated as a fixed effect to estimate the Best Linear Unbiased Estimates (BLUEs), and weight (inverse of squared standard error) for each hybrid to be used in the second stage of the analyses.

$$y_{ikj}= \alpha+ h_{i}+ \varphi_{k}+ \rho_{j}+ \varepsilon_{ikj} (1)$$

where $y_{ikj}$ represents the yield value of the $i$^th^ hybrid in the $k$^th^ block and the $j$^th^ replication. The overall mean is denoted by α, the random effect of the $a$^th^ hybrid is represented by $h_{i}$ , $\varphi_{k}$ is the effect of b^th^ replication, $\rho_{j}$ is the effect of the kth block, and $\varepsilon_{ikj}$ is the random residual effect attributed to the yield value of the $i$^th^ hybrid in the $k$^th^ block and in the $j$^th^ replication that follows the normal distribution $N\left( 0, \sigma_{e}^{2} \right).$

Secondly, we used the model represented by equation 2 (Mackay et al., 2011) to analyze the BLUE values of grain yield and other traits obtained from equation 1 under STRINF and STRNON conditions.

.(Mackay et al.,, 2011) $Y_{ijklm}= \mu+ \zeta_{i}+ \eta_{m}+ \lambda_{jlm}+ \beta_{kjlm}+ {(\zeta\eta)}_{im}+ \xi_{lm}+ \varepsilon_{ijklm} (2)$

$Y_{ijklm}$ is the BLUE value of yield of the $i$^th^ hybrid in the $k$^th^ block, in the $j$^th^ replication, in the $l$^th^ location and $m$^th^ year. The overall mean is represented by $\mu$. The effect of the $i$^th^ hybrid is denoted by $\zeta_{i}$ and the effect of the $m$^th^ year is represented by $\eta_{m}$; $\lambda_{jlm}$ represents the effect of the $j$^th^ replication in the $l$^th^ location and the $m$^th^ year. Also, $\beta_{kjlm}$ is the effect of the $k$^th^ block in $j$^th^ replication in the $l$^th^ location and $m$^th^ year. The interaction effect between the $(im)$^th^ hybrid and year is represented by $({\zeta\eta)}_{im}$, while $\xi_{lm}$ is the effect of location $l$ within year $m$; $\varepsilon_{ijklm}$ is the residual, attributable to the combined effects of within-trial error and hybrid x location within-year interaction. Heterogeneity in the residual variances was assumed. Thus, we fitted a distinct residual variance for each condition for every combination of location and year.

Lastly, we used weighted regression to examine trends (realized genetic gain) in the data in from the second stage of the analyses. The BLUEs of the hybrids and their year of origin in each condition were used as dependent and independent variables in the regression respectively. We determined the percentage of each condition's genetic gain that could be attributed to genetic factors. The process involved calculating the ratio of the second stage's weighted regression slope plus the regression's y-intercept, and the product of the slope and year of the initial testing. The Best Linear Unbiased Predictions (BLUPs) yield of the hybrids in each environment from the first stage was visualized using the Boxplot. Equally, the repeatability (heritability) estimate of each trait over the years by location and condition was presented using a scatter plot. All analyses were performed in R (R Core Team., 2023) using the ASReml package (Gilmour et al., 2009).

3. **Results**

**3.1 Genetic gains in grain yield and *Striga* adaptive traits**

During the 12 years of early maturing hybrids’ genetic improvements, significant annual gains in grain yield of 3.40 and 3.71 % with changes of 76.87 and 127.02 kg ­ha^−1^ ­yr^−1^ were recorded under STRINF and STRNON conditions, respectively. The significant increases in grain yield were associated with significant increases of 1.84 and 1.79 % in ears per plant and significant decreases of 1.31 and 1.75 % in the ear aspect under STRINF and STRNON conditions, respectively. Under the STRINF condition, significant genetic gains of -3.04 and -2.25 % in *Striga* damage rating at 8 and 10 WAP, respectively, and -1.27 % in the number of emerged *Striga* plants at 10WAP were obtained (Table 2). However, there were no significant gains in anthesis silking interval under STRINF environments and days to anthesis and silking under STRINF and STRNON conditions (Table 2). Additionally, the regression line considering the hybrid’s first year of evaluation (independent variable) and traits (dependent variable) showed a positive linear relationship for grain yield and EPP and a negative linear relationship for ESP10, SDR10, and EASP. The breeding period under both conditions did not affect the number of days to flowering and ASI across STRINF and STRNON conditions (Figure 1a-1f).

Table 2: Percent genetic gains in grain yield and other *Striga*-adaptive traits of maize hybrids evaluated under STRINF and STRNON conditions in Nigeria between 2010 –2021.

| **Trait** | **Management** | **% Genetic Gain (yearr^−1^)** | **slope** | **slope.sd** | **intercept** |
| --- | --- | --- | --- | --- | --- |
| YLD | STRINF | 3.40** | 76.87 | 13.32 | -152175.53 |
|  | STRNON | 3.71** | 127.02 | 21.65 | -252000.76 |
| DA | STRINF | 0.12ns | 0.17 | 0.03 | -283.37 |
|  | STRNON | 0.11ns | 0.06 | 0.04 | -63.30 |
| DS | STRINF | 0.10ns | 0.05 | 0.03 | -52.78 |
|  | STRNON | 0.11ns | 0.06 | 0.05 | -72.47 |
| ASI | STRINF | -0.60ns | -0.01 | 0.02 | 32.56 |
|  | STRNON | -5.62** | -0.13 | 0.02 | 272.06 |
| EASP | STRINF | -1.31** | -0.07 | 0.01 | 145.94 |
|  | STRNON | -1.75** | -0.08 | 0.02 | 167.23 |
| EPP | STRINF | 1.84** | 0.01 | 0.00 | -25.00 |
|  | STRNON | 1.79** | 0.01 | 0.00 | -26.62 |
| ESP8 | STRINF | -0.97ns | -0.31 | 0.22 | 653.83 |
| ESP10 | STRINF | -1.27* | -0.45 | 0.20 | 948.38 |
| SDR8 | STRINF | -3.04** | -0.16 | 0.02 | 328.14 |
| SDR10 | STRINF | -2.25** | -0.13 | 0.02 | 274.04 |

Sd:standard deviation; ** : Significant; ns : Not Significant

**3.2 Grain yield performance of early maturing maize hybrids evaluated in the regional trials across STRINF and STRNON conditions, 2010 –2021.**

In the present study, three types of hybrids were evaluated, MST, SCO and LOC. The LOC cultivars comprised five hybrids, among them three common cultivars were evaluated over the period of 12 years. Additionally, the MST hybrids were evaluated for 2-6 years making the data well connected to allow an estimate of realized genetic gains. We assessed the yield gains under STRINF and STRNON conditions by comparing the MST hybrids' average grain yield performance to the SCO and LOC hybrids (Figure 2). The results showed that, under STRINF, the mean grain yield of MST hybrids ranged from 1091 kg ha^−1^ for the hybrid developed in 2012 to 4287 kg ha^−1^ for the hybrid developed in 2018. Similarly, the mean grain yield for SCO hybrids varied from 965 kg ha^−1^ to 2964 kg ha^−1^ and LOC hybrids varied from 1914 kg ha^−1^ to 2644 kg ha^−1^. Contrarily, the mean grain yield under STRNON conditions ranged from 2268 kg ha^−1^ to 6861 kg ha^−1^ for the MST hybrids and from 3590 kg ha^−1^ to 6644 kg ha^−1^ for SCO hybrids while 2392 kg ha^−1^ to 4088 kg ha^−1^ were recorded for LOC hybrids. Generally, the MST hybrids had higher mean grain yields than SCO and the LOC hybrids under STRINF and STRNON conditions, (Figure 2).

**3.3 Repeatability estimates of the STRINF and STRNON trials**

The results of the repeatability estimates of the trials conducted in the STRINF and STRNON conditions from 2010 to 2021 are shown in Table 3 and Figure 3a−f. Grain yield had repeatability estimates ranging from 0.00 for the trial conducted under STRINF conditions at Mokwa in 2016 to 0.91 for the STRINF trial evaluated at Abuja during the 2017 growing season. Among the trials evaluated in Abuja and Mokwa under STRINF and STRNON conditions, only three trials (about 8 %) possessed repeatability estimates less than 30% for grain yield, about 92% of the trials had repeatability greater than 30 % for grain yield (Figure 3). MST, SCOSimilarly, days to anthesis had the repeatability estimates varied from 0.34 at Mokwa under STRINF in 2010 to 0.97 at Abuja in 2017 under STRINF conditions. High repeatability estimates were observed in days to silking and varied from 0.59 under STRINF at Mokwa in 2010 to 0.95 at Abuja in 2017 under STRINF. Anthesis silking interval, ear aspect and ears per plant have repeatability ranging from 0.00 at Mokwa under STRNON in 2020 to 0.81 at Abuja under STRINF in 2016, 0.00 at Mokwa under STRNON in 2015 to 0.92 at Mokwa under STRINF in 2020 and 0.00 at Mokwa under STRNON in 2015 to 0.84 at Abuja under STRINF in 2019, respectively (Table 3, figure 3). Furthermore, repeatability estimates under SRTINF environments varied from 0.10 in 2010 to 0.82 in 2020 at Abuja for ESP8 and from 0.00 in 2015 to 0.82 in 2020 at Abuja for ESP10 while 0.40 in 2010 at Abuja to 0.93 in 2020 at Mokwa for SDR8 and from 0.52 in 2016 to 0.95 at Mokwa in 2020 for SDR10 (Table 3, Figure 3).

Table 3. Repeatability estimate of grain yield and other *Striga*-adaptive traits evaluated under STRINF and STRNON conditions at Mokwa and Abuja between 2010 –2021

| **Trait** | **Repeatability** | | **Year** | **Location** | **Management** |
| --- | --- | --- | --- | --- | --- |
| YLD | minimum | 0.00 | 2016 | Mokwa | STRINF |
|  | maximum | 0.91 | 2017 | Abuja | STRINF |
| DA | minimum | 0.34 | 2010 | Mokwa | STRINF |
|  | maximum | 0.97 | 2017 | Abuja | STRINF |
| DS | minimum | 0.59 | 2010 | Mokwa | STRINF |
|  | maximum | 0.95 | 2017 | Abuja | STRINF |
| ASI | minimum | 0.00 | 2020 | Mokwa | STRNON |
|  | maximum | 0.81 | 2016 | Abuja | STRINF |
| EASP | minimum | 0.00 | 2015 | Mokwa | STRNON |
|  | maximum | 0.92 | 2020 | Mokwa | STRINF |
| EPP | minimum | 0.00 | 2015 | Mokwa | STRNON |
|  | maximum | 0.84 | 2019 | Abuja | STRINF |
| ESP8 | minimum | 0.10 | 2010 | Abuja | STRINF |
|  | maximum | 0.82 | 2020 | Abuja | STRINF |
| ESP10 | minimum | 0.00 | 2015 | Abuja | STRINF |
|  | maximum | 0.82 | 2020 | Abuja | STRINF |
| SDR8 | minimum | 0.40 | 2010 | Abuja | STRINF |
|  | maximum | 0.93 | 2020 | Mokwa | STRINF |
| SDR10 | minimum | 0.52 | 2016 | Mokwa | STRINF |
|  | maximum | 0.95 | 2020 | Mokwa | STRINF |

4. **Discussion**

In addressing the challenges of chronic food insecurity, climate change, environmental sustainability, and improving the economic well-being of farmers in SSA, we used 12 years of historical data to explore three aspects. i) Accelerated gains in grain yield and *Striga* resistance indicator traits in early maturing tropical maize hybrids, to enable plant breeders to justify the most effective use of the genetic variability in early maturing tropical maize populations of IITA. ii) Compare the performance of Striga-tolerant hybrids developed in the breeding program to marketed farmer-preferred cultivars and hybrids developed by multinational seed companies and released in many African countries, thus justifying the program's resource allocation. iii) Assess the repeatability of the trials in the exploration of the hybrids’ genotypic potential in both STRINF and STRNON conditions, hence, this allows plant breeders to develop maize hybrids that could withstand the devastating effects of Striga infestation in Striga-prone environments. These, in turn, support rural development and poverty alleviation.

To make the necessary adjustments in the investigation of genotype potential and/or introduce variability into the breeding populations, it is essential to ascertain the rate of genetic advancement in a breeding program during a specific period of genetic enhancement.  In the present study, significant progress was attained during 12 years of genetic enhancements with annual gains in grain yield reaching 3.40 % yr^−1^ and 3.71 % yr^−1^ under STRINF and STRNON conditions, respectively. The gains are associated with relative changes of 76.87 kg ­ha^−1^ ­yr^−1^ and 127.02 kg ­ha^−1^ ­yr^−1^, respectively. The progress attained in the present study was considerably higher compared to annual genetic gains in grain yield of 2.91 % yr^−1^ and 1.33 % yr^−1^ under low and high N environments, respectively, (Badu-Apraku et al., 2022b), 1.93 % yr^−1^ under drought and well-watered conditions (Oyekunle et al., 2019b), and 0.74 % yr^−1^ and 1.47 % yr^−1^ under drought and full irrigation, respectively (Menkir et al., 2022a). Additionally, (Beyene & Prasanna, n.d.) estimated genetic gains of 2.17 % yr^−1^ and 2.12 % yr^−1^ under low N and drought stress, respectively, using the historical data in late maturing hybrids and 2.6 % under drought in early maturing hybrids. However, (Badu-Apraku et al., (2020) reported genetic gains of 4.82 % yr^−1^ and 1.24 % yr^−1^ under STRINF and STRNON conditions, respectively in early maturing maize hybrids, and (Menkir et al., (2022) reported 2.58 % yr^−1^ and 2.30 % yr^−1^ under STRINF and STRNON conditions, respectively, in late-maturing maize hybrids. This implied that under Striga infestation, hybrids respond to selection more favorably than under drought or low N stresses. This might be because artificial Striga infestation trials are conducted in more controlled environments than under drought and low N stress conditions, allowing for better accuracy in the selection process. In addition, the *Striga* resistance may be more oligogenic than polygenic which is mostly found in drought and low N tolerance. Since the artificial Striga infestation process utilized for the development of inbred lines and the evaluation of hybrids remained unchanged for the last two decades, it could continuously result in high levels of precision, which had a major impact on achieving a higher rate of genetic gain in grain yield under STRINF. Alternatively, it could be because more traits are observed during a Striga infestation trial than during a drought or low N trial, which could aid breeders in precisely selecting the most promising hybrids. Furthermore, the low yield improvements observed under drought or low N stresses may be related to the adoption of Striga-resistant populations enhanced through S_1_ recurrent selection without the infusion of tropical donor lines possessing superior genes for drought or low N tolerance as parents of the MST hybrids (Menkir et al., 2022b) . The significant increases in grain yield were associated with significant increases of 1.84 % yr^−1^ and 1.79 % yr^−1^ in ears per plant and significant decreases of 1.31 % yr^−1^ and 1.75 % yr^−1^ in the ear aspect under STRINF and STRNON conditions, respectively. Additionally, the increases in grain yield under STRINF conditions were accompanied by a significant decrease of 3.04 % yr^−1^, 2.25 % yr^−1^, and 1.27 % yr^−1^ in Striga damage ratings at 8 and 10 WAP and the number of emerged Striga plants at 10 WAP, respectively. Similar results were obtained by (Badu-Apraku et al., 2023a; Menkir et al., 2022b) Interestingly, the 12 years breeding period did not affect the flowering period of the early-maturing maize hybrids.

The success of the breeding program depends on the establishment of the right selection criteria and the capacity to select hybrids that are superior in performance and/or competitive in grain yields under stressful conditions and across diverse environments. The comparative performance of MST hybrids to LOC and SCO hybrids across both STRINF and STRNON conditions showed a clear distinction between the hybrids of the three groups. The MST hybrids performed exceptionally well in both STRINF and STRNON conditions. The result confirmed that MST with improved levels of Striga resistance outperformed those of SCO and LOC hybrids in contrasting environments. Our findings suggested that the breeding strategies, including recurrent selection, backcrossing, hybridization, evaluation, and selection, used to develop the MST hybrids during the 12-year breeding period were highly effective. This implied that MST hybrids possess favorable genes that make them better performers than the SCO and LOC hybrids. Therefore, the accumulation of new favorable alleles through rapid breeding cycles is one of the possible scenarios that significantly increases the rate of gains.

Evaluating the repeatability of the STRINF and STRNON trials for assessing and exploring the hybrids' genotypic potential was a focus of the present study. Unreliable or inconsistent repeatability estimates of the trials could hinder the progress of accelerated genetic gains, as researchers could waste precious time and resources building on flawed or irreproducible findings. Reliable methodologies and experimental designs contribute to the overall quality of scientific experiments. Rigorous methods help ensure that the results are a true reflection of the phenomenon under investigation, rather than being influenced by confounding variables or errors. In summary, repeatability is essential in the assessment of the trial because it ensures the accuracy, consistency, trustworthiness, and validity of the trial. When the random error is restricted, the variations observed in the trial would result solely in genotypic differences, environment, and genotype*environment (G*E) interaction. Nonetheless, the overall observed variation in the experiment was considerably influenced by genotype, environment, and G*E interaction, determining the repeatability of the trial, taking into account the combined effects of genotype, environment, G*E interaction, and random error. Therefore, the disparities in the repeatability estimates of the trials conducted over a long period may be mostly due to the experimental procedures used/random error. We estimated the repeatability of the STRINF and STRNON trials conducted between 2010 and 2021 for each of the measured traits. The highest repeatability was observed in ears per plant and flowering dates, indicating that these traits would not be largely influenced by the environment, G*E interaction. This suggested that flowering dates and ears per plant could be used to evaluate the effectiveness of the STRINF and STRNON trials in the exploration of the genotypic potential of the hybrids. However, the lowest repeatability was observed in the emerged *Striga* plant at 8 and 10 WAP. Thus, the emerged *Striga* plant at 8 and 10 WAP should not be used to determine the efficiency of the STRINF and STRNON trials in the exploration of the hybrids’ genotypic potential. This is not surprising because maize hybrids had different stimulations of strigolactone, which may lead to uneven germination of S*triga* seeds and their attachment to the host (Jamil et al., 2012). Additionally, a combination of factors encountered during trial evaluation at each location concerning distinct soil properties, nutrient contents, and pH levels, could affect the emergence of the *Striga* plant (S.-K. Kim, 1996). Generally, a good level of repeatability was observed in early maturing maize trials conducted under STRINF and STRNON conditions between the 2010 and 2021 growing seasons, indicating the potential of the trials in the identification of promising hybrids for commercialization in the SSA for food security and poverty alleviation.

5. **Conclusion**

The research used 12 years of historical data to analyze the genetic gains in grain yield and changes in yield-related traits under STRINF and STRNON conditions and the repeatability of the trials. The results showed significant progress in grain yield, with annual gains of 3.40% yr^−1^ and 3.71 % yr^−1^ obtained under STRINF and STRNON conditions, respectively. The MST hybrids, with improved *Striga* resistance, outperformed the LOC and SCO hybrids in both STRINF and STRNON conditions. The breeding strategies used during the 12-year breeding period were effective, suggesting that MST hybrids possessed favorable genes that contributed to their better performance. The flowering dates and ears per plant possess the highest repeatability estimates, these traits have the lowest multi-dimensionalities in estimated repeatability and were not largely influenced by environment, and G*E interaction. However, the emerged *Striga* plant at 8 and 10 WAP had the highest multi-dimensionality, suggesting that these traits should not be used to determine the efficiency of the trials in exploring the hybrids' genotypic potential. Generally, a good level of repeatability was observed in early maturing maize trials, indicating the quality of the STRINF and STRNON trials in an exploration of outstanding hybrids for commercialization in the SSA for food security and poverty alleviation.

**Data availability statement**

The raw data used/analyzed in this manuscript were available in IITA CKAN

**Funding**

This research was supported by the Bill & Melinda Gates Foundation [OPP1134248] under the Accelerated Genetic Gains (AGG) and Stress Tolerant Maize for Africa (STMA) Projects.

**Acknowledgments**

The authors are grateful to the IITA-MIP, EBS and Biometric Unit technical, field, and administrative staff for providing technical, field, and administrative assistance.

**Conflict of interest**

The authors declare that there is no conflict of interest.

Reference

Abdulmalik, R. O., Menkir, A., Meseka, S. K., Unachukwu, N., Ado, S. G., Olarewaju, J. D., Aba, D. A., Hearne, S., Crossa, J., & Gedil, M. (2017). Genetic gains in grain yield of a maize population improved through marker assisted recurrent selection under stress and non-stress conditions in West Africa. *Frontiers in Plant Science*, *8*. https://doi.org/10.3389/fpls.2017.00841

Akaogu, I. C., Badu-Apraku, B., Tongoona, P., Ceballos, H., Gracen, V., Offei, S. K., & Dzidzienyo, D. (2019). Inheritance of Striga hermonthica adaptive traits in an early-maturing white maize inbred line containing resistance genes from Zea diploperennis. *Plant Breeding*, *138*(5). https://doi.org/10.1111/pbr.12707

Badu-Apraku, B., Abubakar, A. M., Adu, G. B., Yacoubou, A.-M., Adewale, S., & Adejumobi, I. I. (2023a). Enhancing Genetic Gains in Grain Yield and Efficiency of Testing Sites of Early-Maturing Maize Hybrids under Contrasting Environments. *Genes*, *14*(10), 1900. https://doi.org/10.3390/genes14101900

Badu-Apraku, B., Abubakar, A. M., Adu, G. B., Yacoubou, A.-M., Adewale, S., & Adejumobi, I. I. (2023b). Enhancing Genetic Gains in Grain Yield and Efficiency of Testing Sites of Early-Maturing Maize Hybrids under Contrasting Environments. *Genes*, *14*(10), 1900. https://doi.org/10.3390/genes14101900

Badu-Apraku, B., Adu, G. B., Yacoubou, A. M., Toyinbo, J., & Adewale, S. (2020). Gains in genetic enhancement of early maturing maize hybrids developed during three breeding periods under striga-infested and striga-free environments. *Agronomy*, *10*(8). https://doi.org/10.3390/agronomy10081188

Badu-Apraku, B., & Fakorede, M. A. B. (2017). Advances in genetic enhancement of early and extra-early maize for sub-Saharan Africa. In *Advances in Genetic Enhancement of Early and Extra-Early Maize for Sub-Saharan Africa*. https://doi.org/10.1007/978-3-319-64852-1

Badu-Apraku, B., Fakorede, M. A. B., & Abubakar, A. M. (2022a). Accelerated Genetic Gains in Early-Maturing Maize Hybrids following Three Periods of Genetic Enhancement for Grain Yield under Low and High Soil-Nitrogen Environments. *Plants*, *11*(9). https://doi.org/10.3390/plants11091208

Badu-Apraku, B., Fakorede, M. A. B., & Abubakar, A. M. (2022b). Accelerated Genetic Gains in Early-Maturing Maize Hybrids following Three Periods of Genetic Enhancement for Grain Yield under Low and High Soil-Nitrogen Environments. *Plants*, *11*(9). https://doi.org/10.3390/plants11091208

Beyene, Y., & Prasanna, B. M. (n.d.). *Genetic Gains in CIMMYT Maize Breeding Program in Africa with CIMMYT-GMP colleagues in Africa*.

Crespo-Herrera, L. A., Crossa, J., Huerta-Espino, J., Autrique, E., Mondal, S., Velu, G., Vargas, M., Braun, H. J., & Singh, R. P. (2017). Genetic yield gains in CIMMYT’S international elite spring wheat yield trials by modeling the genotype × environment interaction. *Crop Science*, *57*(2), 789–801. https://doi.org/10.2135/cropsci2016.06.0553

Cullis, B. R., Smith, A. B., & Coombes, N. E. (2006). On the design of early generation variety trials with correlated data. *Journal of Agricultural, Biological, and Environmental Statistics*, *11*(4). https://doi.org/10.1198/108571106X154443

de la Vega, A. J., DeLacy, I. H., & Chapman, S. C. (2007). Progress over 20 years of sunflower breeding in central Argentina. *Field Crops Research*, *100*(1). https://doi.org/10.1016/j.fcr.2006.05.012

Duvick, D. N. (2005). Genetic progress in yield of United States maize (Zea mays L.). In *Maydica* (Vol. 50, Issues 3–4).

Echarte, L., Nagore, L., Di, J., Cambareri, M., Robles, M., & Della, A. (2013). Grain Yield Determination and Resource Use Efficiency in Maize Hybrids Released in Different Decades. In *Agricultural Chemistry*. https://doi.org/10.5772/55287

Ejeta, G. (2007). The striga scourge in africa: A growing pandemic. In: Ejeta G, Gressel J, editors. Integrating New Technologies for Striga Control: Towards Ending the Witch-Hunt. *World Scientific Publishing Company*.

Gilmour, a R., Gogel, B. J., Cullis, B. R., & Thompson, R. (2009). ASReml user guide release 3.0. *VSN International Ltd*.

Jamil, M., Kanampiu, F. K., Karaya, H., Charnikhova, T., & Bouwmeester, H. J. (2012). Striga hermonthica parasitism in maize in response to N and P fertilisers. *Field Crops Research*, *134*. https://doi.org/10.1016/j.fcr.2012.03.015

Kim, S.-K. (1996). Horizontal resistance: core to a research breakthrough to combat Striga in Africa. *Integrated Pest Management Reviews*, *1*(4). https://doi.org/10.1007/bf00139766

Kim, S. K., Adetimirin, V. O., Thé, C., & Dossou, R. (2002). Yield losses in maize due to Striga hermonthica in West and Central Africa. *International Journal of Pest Management*, *48*(3). https://doi.org/10.1080/09670870110117408

Kim, S. K., Akintunde, A. Y., & Walker, P. (1999). Responses of maize inbreds during development of Striga hermonthica infestation. *Maydica*, *44*(4).

Kim, S. K., IITA, ICRISAT, & IDRC. (1988). Breeding maize for striga tolerance and the development of a field infestation technique. *Combating Striga in Africa*.

Kountche, B. A., Jamil, M., Yonli, D., Nikiema, M. P., Blanco-Ania, D., Asami, T., Zwanenburg, B., & Al-Babili, S. (2019). Suicidal germination as a control strategy for Striga hermonthica (Benth.) in smallholder farms of sub-Saharan Africa. *Plants People Planet*, *1*(2). https://doi.org/10.1002/ppp3.32

Mackay, I., Horwell, A., Garner, J., White, J., McKee, J., & Philpott, H. (2011). Reanalyses of the historical series of UK variety trials to quantify the contributions of genetic and environmental factors to trends and variability in yield over time. *Theoretical and Applied Genetics*, *122*(1). https://doi.org/10.1007/s00122-010-1438-y

Masuka, B., Atlin, G. N., Olsen, M., Magorokosho, C., Labuschagne, M., Crossa, J., Bänziger, M., Pixley, K. V., Vivek, B. S., Von Biljon, A., Macrobert, J., Alvarado, G., Prasanna, B. M., Makumbi, D., Tarekegne, A., Das, B., Zaman-Allah, M., & Cairns, J. E. (2017). Gains in maize genetic improvement in eastern and southern Africa: I. CIMMYT hybrid breeding pipeline. *Crop Science*, *57*(1). https://doi.org/10.2135/cropsci2016.05.0343

Menkir, A., Dieng, I., Meseka, S., Bossey, B., Mengesha, W., Muhyideen, O., Riberio, P. F., Coulibaly, M., Yacoubou, A. M., Bankole, F. A., Adu, G. B., & Ojo, T. (2022a). Estimating genetic gains for tolerance to stress combinations in tropical maize hybrids. *Frontiers in Genetics*, *13*. https://doi.org/10.3389/fgene.2022.1023318

Menkir, A., Dieng, I., Meseka, S., Bossey, B., Mengesha, W., Muhyideen, O., Riberio, P. F., Coulibaly, M., Yacoubou, A. M., Bankole, F. A., Adu, G. B., & Ojo, T. (2022b). Estimating genetic gains for tolerance to stress combinations in tropical maize hybrids. *Frontiers in Genetics*, *13*. https://doi.org/10.3389/fgene.2022.1023318

Menkir, A., & Meseka, S. (2019). Genetic improvement in resistance to striga in tropical maize hybrids. *Crop Science*, *59*(6). https://doi.org/10.2135/cropsci2018.12.0749

Mwangangi, I. M., Büchi, L., Haefele, S. M., Bastiaans, L., Runo, S., & Rodenburg, J. (2021). Combining host plant defence with targeted nutrition: key to durable control of hemiparasitic Striga in cereals in sub-Saharan Africa? In *New Phytologist* (Vol. 230, Issue 6). https://doi.org/10.1111/nph.17271

Oyekunle, M., Ado, S. G., Usman, I. S., Abdulmalik, R. O., Ahmed, H. O., Hassan, L. B., & Yahaya, M. A. (2019a). Gains in grain yield of released maize (Zea mays L.) cultivars under drought and well-watered conditions. *Experimental Agriculture*, *55*(6), 934–944. https://doi.org/10.1017/S0014479719000048

Oyekunle, M., Ado, S. G., Usman, I. S., Abdulmalik, R. O., Ahmed, H. O., Hassan, L. B., & Yahaya, M. A. (2019b). Gains in grain yield of released maize (Zea mays L.) cultivars under drought and well-watered conditions. *Experimental Agriculture*, *55*(6), 934–944. https://doi.org/10.1017/S0014479719000048

Piepho, H. P., & Möhring, J. (2006). Selection in cultivar trials—Is it ignorable? *Crop Science*, *46*(1). https://doi.org/10.2135/cropsci2005.04-0038

Prasanna, B. M., Burgueño, J., Beyene, Y., Makumbi, D., Asea, G., Woyengo, V., Tarekegne, A., Magorokosho, C., Wegary, D., Ndhlela, T., Zaman-Allah, M., Matova, P. M., Mwansa, K., Mashingaidze, K., Fato, P., Teklewold, A., Vivek, B. S., Zaidi, P. H., Vinayan, M. T., … Cairns, J. E. (2022). Genetic trends in CIMMYT’s tropical maize breeding pipelines. *Scientific Reports*, *12*(1). https://doi.org/10.1038/s41598-022-24536-4

R Core Team. (2023). R: A language and environment for statistical computing. R Foundation for Statistical Computing, Vienna, Austria. *Open Journal of Statistics*, *13*(02).
