# Supplementary material for: Repeatability and genetic advances in early maturing maize hybrid trials conducted under Striga-infested and non-infested conditions
Source: PLoS One. 2025 Apr 3;20(4):e0319353. doi: 10.1371/journal.pone.0319353 (PMC11967925; doi:10.1371/journal.pone.0319353)
Supplement: Attachment — Submitted filename: Response to Reviewers.docx [file pone.0319353.s003.docx]

**REBUTTAL TO REVIEWERS’ COMMENTS ON THE MANUSCRIPT TITLED “ REPEATABILITY AND GENETIC ADVANCES IN EARLY MATURING MAIZE HYBRID TRIALS CONDUCTED UNDER STRIGA-INFESTED AND NON-INFESTED CONDITIONS”**

**Response to Editor (Journal requirements)**

1. Please ensure that your manuscript meets PLOS ONE's style requirements, including those for file naming.

**Response:** The manuscript has been modified to meet PLOS ONE's style requirements.

2. Please note that funding information should not appear in any section or other areas of your manuscript. We will only publish funding information present in the Funding Statement section of the online submission form. Please remove any funding-related text from the manuscript.

**Response:** The funding-related text has been removed from the manuscript

3. We note that the grant information you provided in the ‘Funding Information’ and ‘Financial Disclosure’ sections do not match. When you resubmit, please ensure that you provide the correct grant numbers for the awards you received for your study in the ‘Funding Information’ section.

**Response:** The funding information will be corrected upon resubmission of the revised manuscript.

4. Thank you for stating the following financial disclosure: “This research was supported by the Bill & Melinda Gates Foundation under the Accelerated Genetic Gains (AGG) and Stress Tolerant Maize for Africa (STMA) Projects.”

Please state what role the funders took in the study. If the funders had no role, please state: "The funders had no role in study design, data collection and analysis, decision to publish, or preparation of the manuscript." If this statement is not correct you must amend it as needed. Please include this amended Role of Funder statement in your cover letter; we will change the online submission form on your behalf.

**Response:** The funder was the main donor of the AGG and STMA projects and also the payer of the article processing charge for the manuscript.

5. Thank you for uploading your study's underlying data set. Unfortunately, the repository you have noted in your Data Availability statement does not qualify as an acceptable data repository according to PLOS's standards. At this time, please upload the minimal data set necessary to replicate your study's findings to a stable, public repository (such as figshare or Dryad) and provide us with the relevant URLs, DOIs, or accession numbers that may be used to access these data. For a list of recommended repositories and additional information on PLOS standards for data deposition, please see <https://journals.plos.org/plosone/s/recommended-repositories>

**Response:** The acceptable data repository according to PLOS's standards with DOIs have now been provided in the data availability statement of the manuscript

6. When completing the data availability statement of the submission form, you indicated that you will make your data available on acceptance. We strongly recommend all authors decide on a data sharing plan before acceptance, as the process can be lengthy and hold up publication timelines. Please note that, though access restrictions are acceptable now, your entire data will need to be made freely accessible if your manuscript is accepted for publication. This policy applies to all data except where public deposition would breach compliance with the protocol approved by your research ethics board. If you are unable to adhere to our open data policy, please kindly revise your statement to explain your reasoning and we will seek the editor's input on an exemption. Please be assured that, once you have provided your new statement, the assessment of your exemption will not hold up the peer review process.

**Response:** The data have now been made available.

7. We note you have included a table to which you do not refer in the text of your manuscript. Please ensure that you refer to Table 1 in your text; if accepted, production will need this reference to link the reader to the Table.

**Response:** We have made appropriate reference to Table 1 in the revised version of the manuscript

8. Please include captions for your Supporting Information files at the end of your manuscript, and update any in-text citations to match accordingly. Please see our Supporting Information guidelines for more information: <http://journals.plos.org/plosone/s/supporting-information>.

**Response:** Captions for Supporting Information files have now been provided at the end of the manuscript

9. Please review your reference list to ensure that it is complete and correct. If you have cited papers that have been retracted, please include the rationale for doing so in the manuscript text, or remove these references and replace them with relevant current references. Any changes to the reference list should be mentioned in the rebuttal letter that accompanies your revised manuscript. If you need to cite a retracted article, indicate the article’s retracted status in the References list and also include a citation and full reference for the retraction notice.

**Response:** The list of references has been reviewed to ensure that it is complete and correct. We observed that a reference was not cited appropriately which was taken care of. In addition, duplicate references have also been resolved in the revised version of the manuscript.

Responses to Reviewers' Comments

Reviewer #1: The quality of the manuscript is technically sound, and data addressed all objectives. However, some concerns are:

1. Results should be reported in the past tense.

**Response:** The results section of the manuscript has been reported in the past tense as requested.

2. Some minor grammatical errors need correction; a few have already been pointed out.

**Response:** We have revised the manuscript for grammatical modifications and sentence chronology to improve readability in the revised version of the manuscript as requested.

3. Figures addressing Objective 2 are missing.

**Response:** We apologize for this however all figures relevant to the work were submitted separately to the PLOS ONE webpage. We hope the editor can resolve this issue in the subsequent interactive review.

4. Please refer to Table 3 and recast your results on repeatability values for the traits.

**Response:** As requested, we have recast the results relating to Table 3 to ensure accurate interpretation for readers.

5. Indicate in the Materials and Methods section the method used for assessing the significance of values.

**Response:** The method used for assessing the significance of the values have been included in the Materials and Methods section as requested in the revised manuscript.

6. Title can be rephrased as "Repeatability and genetic gain"

**Response:** We appreciate your suggestion to improve the quality of the manuscript. The co-authors have several similar publications with titles including the word “genetic gains”. We would like to use the word “genetic advances” as a replacement for “genetic advancement” to provide a sense of uniqueness to the manuscript title.

7. Restructure your conclusions to address the objectives.

**Response:** We have restructured the conclusion section to focus on the objectives in the revised manuscript as requested.

Reviewer #2: The manuscript is well written except for few MINOR REVISIONS to be made before acceptance:

(1) The 'Introduction' section is too long and need to be shortened with good flow of the required information

**Response:** Thank you. We have revised the Introduction as requested.

(2) Correct as 'Materials and Methods'

**Response:** Material and Method were corrected as 'Materials and Methods' as requested.

(3) Present the coordinates of the test locations

**Response:** Coordinates of the test locations have been referenced in the revised manuscript as requested.

(4) Present the agro-climatic pattern of the different locations and years

**Response:** Thank you for your comment. The agro-climatic patterns of the two locations have been included as a supplementary file to be submitted alongside the revised manuscript.

(5) The discussion to be presented with suitable sub-headings for easy understanding to the readers

**Response:** Discussion has now been presented with suitable sub-headings as requested.
