# Supplementary material for: Repeatability and genetic advances in early maturing maize hybrid trials conducted under Striga-infested and non-infested conditions
Source: PLoS One. 2025 Apr 3;20(4):e0319353. doi: 10.1371/journal.pone.0319353 (PMC11967925; doi:10.1371/journal.pone.0319353)
Supplement: Attachment — Submitted filename: Response_to_Reviewer.docx [file pone.0319353.s005.docx]

**REBUTTAL TO REVIEWERS’ COMMENTS ON THE MANUSCRIPT TITLED “REPEATABILITY AND GENETIC ADVANCES IN EARLY MATURING MAIZE HYBRID TRIALS CONDUCTED UNDER STRIGA-INFESTED AND NON-INFESTED CONDITIONS”**

**Response to Reviewers' Comments**

Reviewer #1

**Comment 1**

Please refer to Table 3 and recast your result in abstract and result section. "The results indicated that the number of emerged Striga plants at 8 and 10 WAP recorded the lowest repeatability". What of ASI with 0.81?

**Response 1**

Thank you for your suggestion to improve the quality of this manuscript. The result in Table 3 presented the ranges of the repeatability estimates. Still, Fig 3 indicated the consistency in the repeatability estimates and therefore, the lowest repeatability here referred in terms of consistency and that has now been addressed clearly.

**Comment 2**

Table 2, correct spelling of Genetic gain (yearr) to "year"

**Response 2**

This has been effected in the revised version of the manuscript. Thanks

**Comment 3**

Annual genetic gains of 3.40% yr−1 and 3.71 % yr−1 were obtained under STRINF and STRNON conditions, respectively"for what trait??????". Please indicate clearly.

**Response 3**

The traits have been included in the sentence as requested. Thanks.

**Comment 4**

Recast the last paragraph in conclusion as indicated in the attached manuscript.

**Response 4**

Modified as suggested. We appreciate your observation.
